# mPR-Specific Actions Influence Maintenance of the Blood–Brain Barrier (BBB)

**DOI:** 10.3390/ijms23179684

**Published:** 2022-08-26

**Authors:** Johnathan Abou-Fadel, Xiaoting Jiang, Akhil Padarti, Dinesh G. Goswami, Mark Smith, Brian Grajeda, Muaz Bhalli, Alexander Le, Wendy E. Walker, Jun Zhang

**Affiliations:** 1 Department of Molecular and Translational Medicine (MTM), Texas Tech University Health Science Center El Paso, El Paso, TX 79905, USA; 2 Department of Biological Sciences, University of Texas at El Paso, El Paso, TX 79902, USA

**Keywords:** cerebral cavernous malformations (CCMs), CCM signaling complex (CSC), classic nuclear progesterone receptors (nPRs), nonclassic membrane progesterone receptors (mPRs), CSC-mPRs-PRG (CmP) signaling network, blood–brain barrier (BBB), endothelial cells (ECs), biomarkers

## Abstract

Cerebral cavernous malformations (CCMs) are characterized by abnormally dilated intracranial microvascular sinusoids that result in increased susceptibility to hemorrhagic stroke. It has been demonstrated that three CCM proteins (CCM1, CCM2, and CCM3) form the CCM signaling complex (CSC) to mediate angiogenic signaling. Disruption of the CSC will result in hemorrhagic CCMs, a consequence of compromised blood–brain barrier (BBB) integrity. Due to their characteristically incomplete penetrance, the majority of *CCM* mutation carriers (presumed CCM patients) are largely asymptomatic, but when symptoms occur, the disease has typically reached a clinical stage of focal hemorrhage with irreversible brain damage. We recently reported that the CSC couples both classic (nuclear; nPRs) and nonclassic (membrane; mPRs) progesterone (PRG)-receptors-mediated signaling within the CSC-mPRs-PRG (CmP) signaling network in nPR(−) breast cancer cells. In this report, we demonstrate that depletion of any of the three *CCM* genes or treatment with mPR-specific PRG actions (PRG/mifepristone) results in the disruption of the CmP signaling network, leading to increased permeability in the nPR(−) endothelial cells (ECs) monolayer in vitro. Finally, utilizing our in vivo hemizygous *Ccm* mutant mice models, we demonstrate that depletion of any of the three *CCM* genes, in combination with mPR-specific PRG actions, is also capable of leading to defective homeostasis of PRG in vivo and subsequent BBB disruption, allowing us to identify a specific panel of etiological blood biomarkers associated with BBB disruption. To our knowledge, this is the first report detailing the etiology to predict the occurrence of a disrupted BBB, an indication of early hemorrhagic events.

## 1. Introduction

Cerebral cavernous malformations (CCM) are characterized by abnormally dilated intracranial microvascular sinusoids that result in increased susceptibility to hemorrhagic stroke. At least one of three genes, *KRIT1* (*CCM1*), *MGC4607* (*CCM2*), and *PDCD10* (*CCM3*), is disrupted in most familial CCM cases. It has been demonstrated that both CCM1 and CCM3 proteins bind to CCM2 protein to form the CCM signaling complex (CSC) to mediate angiogenic signaling [1,2,3]. The recent identification of multiple *CCM2* isoforms with distinctive functions [3] and extensive efforts on the comparative omics of the CSC [2,4] make the ongoing investigation of CSC signaling cascades more exciting. Currently, the invasive neurosurgery removal of CCM lesions is the only option for treatment, despite the recurrence of worse symptoms frequently occurring after surgery. Furthermore, as an autosomal dominant disorder with incomplete penetrance, many *CCM* gene mutation carriers are commonly asymptomatic, but when symptoms occur, the disease has typically reached the stage of focal hemorrhage with irreversible brain damage, while the underlying “trigger” for this seemingly “random” occurrence of hemorrhagic CCMs remains elusive [5]. However, our recent reports revealed that the CSC modulates progesterone (PRG)-mediated actions between classic nuclear PRG receptors (nPRs) and nonclassic membrane PRG/progestin and adipoQ receptors (mPRs/PAQRs) within the CSC-mPRs-PRG-nPRs (CmPn) signaling network in two nPR(+) (T47D, MCF7) breast cancer cell lines, and within the CSC-mPRs-PRG (CmP) signaling network in two nPR(−) triple-negative breast cancer cells (TNBCs: MDA-MB-231, MDA-MB-468) [5,6,7,8], shedding light on this mystery. We discovered that a well-known progesterone blocker, mifepristone (MIF, a major contraception drug), inhibits PRG actions only via classic-PRG-receptors-mediated signaling (nPRs) in nPR(+) cells. In contrast, MIF can also act as a PRG agonist alone, and when combined with PRG, sex steroid signaling via nonclassic PRG receptors (mPRs) is synergistically enhanced to form a feedback loop in an mPR-specific fashion (only through mPRs). In this feedback regulation network, the CSC stability is disrupted by the negative effects of mPR-specific actions (MIF alone or combined (PRG/MIF)) via mPRs signaling or the positive effects of nPRs-specific PRG actions (only through nPRs) through classic nPRs signaling [5,6,7,8]. Our discovery reveals that the balance between classic and nonclassic PRG signaling (nPRs/mPRs) impacts the CSC function, and identifies the CSC as an important mediator of nPRs and mPRs crosstalk in nPR(+) cells. Our observations are further supported by a previous finding that PRG can act simultaneously on both nPRs and mPRs, and that activation of mPRs-mediated signaling can potentiate expression of the hormone-activated nPR-2 isoform [9]. All of these data demonstrated the importance of the CSC for coupling both nPRs and mPRs by mediating crosstalk between them, and elucidated that the CSC can be regulated by feedback mechanisms involving PRG and its receptors (nPRs/mPRs) [5,6,7,8]. Furthermore, accumulated evidence has demonstrated that excessive PRG exposure, such as patients under hormone therapy during menopause/post-menopause, or females taking hormonal contraceptives/experiencing pregnancy, may be at an increased risk for hemorrhagic events. Significantly increased PRG levels during early pregnancy has been reported [10], and the increase in size of CCM lesions [11,12] and increased cases of hemorrhagic CCMs during pregnancy have also been observed [13]. Increased risk for acute CCM bleedings [13,14,15], or formation of a de novo CCM lesion [15], has also previously been reported during pregnancies. Finally, our data also demonstrated that a common contraceptive, mifepristone (MIF), competes with PRG for the same binding sites on nPRs as an antagonist for nPR-mediated signaling [6]. In contrast, MIF binds to mPRs allosterically with PRG, and works synergistically with PRG in mPR-mediated signaling as an agonist [6]. Therefore, we have defined the two-sex-steroids-combined treatment (MIF + PRG) as mPR-specific PRG actions [6,7,8]. Together, these results suggest that pregnancy-/contraceptive-associated increased levels of mPR-specific PRG actions lead to increased risks of hemorrhage in CCM patients.

In this report, we investigated the underlying mechanisms of the novel CSC-mPR-PRG (CmP)-mediated signaling network in mainly nPR(−) ECs in vitro, as well as investigating the integrity of the blood–brain barrier (BBB) utilizing *Ccm (1–3)* mutant mice in vivo, to explore the effects of a disrupted CmP network under mPR-specific PRG actions. We also successfully identified a panel of serum etiological biomarkers associated with a disrupted CmP signaling network to predict the occurrence of a disrupted BBB in *Ccm* mutant mice, an early indication for possible hemorrhagic events, which could have enormous clinical applications in stroke prevention.

## 2. Results

### 2.1. CCM2 Is the Cornerstone for the Stability and Functionality of the CSC in Vascular ECs

Our recently defined CmPn/CmP signaling networks not only emphasize the balanced modulation of the CSC (CCM signaling complex) stability through either the positive effects of classic nuclear progesterone (PRG) receptors (nPRs) or negative effects of nonclassic membrane PRG receptors (mPRs/PAQRs), but also demonstrate the existence of feedback regulations in this relationship. The unique role of the CSC was further demonstrated in the crosstalk between nPRs/mPRs-specific signaling in breast cancer cells [7,8,16]. This intricate feedback regulatory cascade is within either the CmPn or CmP signaling network, under mPR-specific PRG actions (PRG-mediated signaling only through mPRs) in nPR(+) or nPR(−) breast cancer cells, respectively. mPR-specific PRG actions can be realized through the balanced efforts between nPRs and mPRs in nPR(+) cells, where feedback regulation can be managed at both transcriptional and translational levels. However, mPR-specific PRG actions are the only signaling pathway that can be utilized by the PRG in nPR(−) cells.

**Figure 1:**
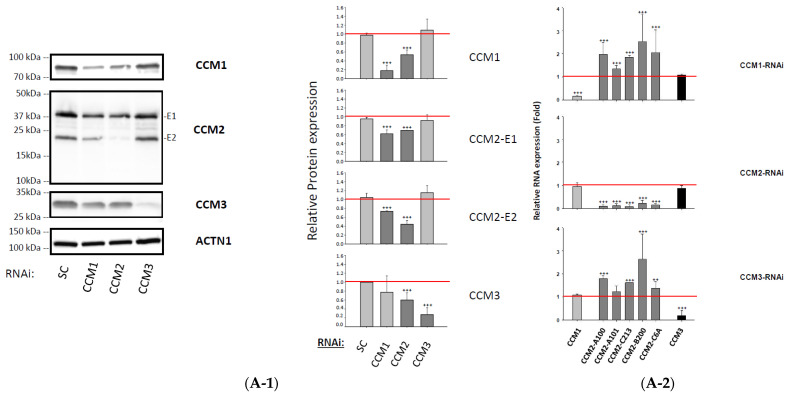

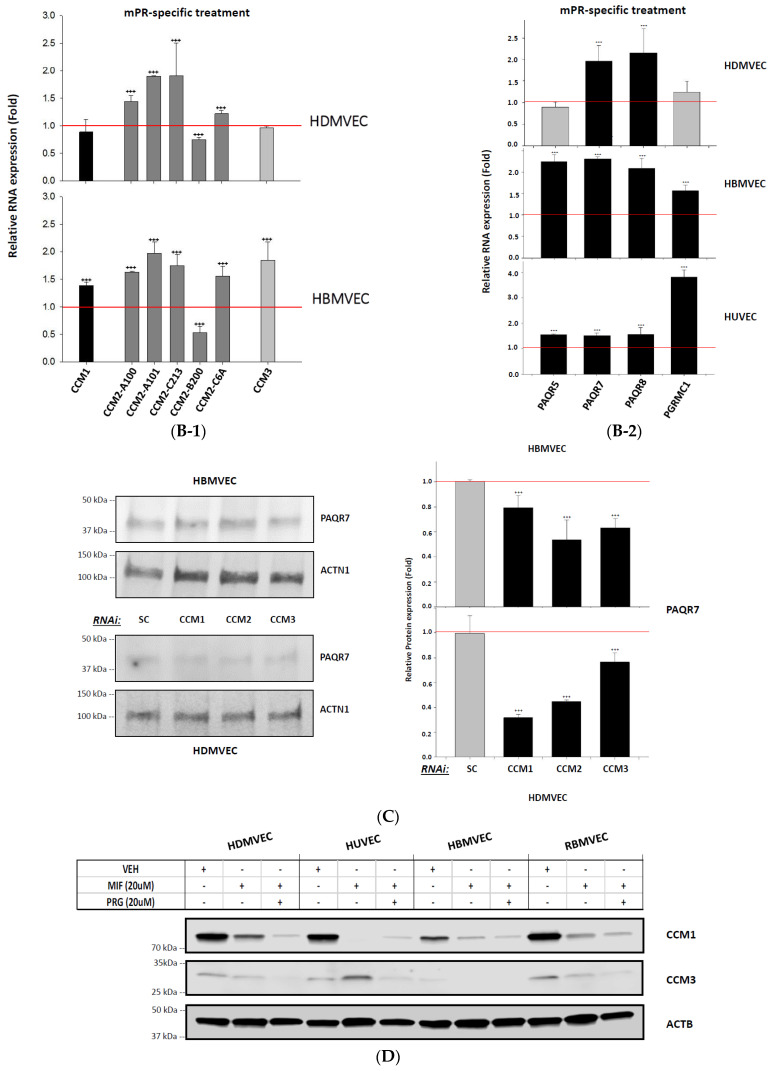
Relationships among key players (CCMs and mPRs) within the CmP network in nPR(−) endothelial cells (ECs). (**A**) Silencing of CCM2 decreases the expression levels of both CCM 1/3 proteins in human brain microvascular endothelial cells (HBMVEC). (**A-1**) After silencing all three CCMs (1, 2, or 3) for 48 h, the expression levels of all three CCM proteins were efficiently targeted and silenced; however, significantly decreased expression of both CCM1/3 proteins was observed in CCM2-KD HBMVEC cells (left upper and lower panels). The relative expression levels of CCMs (1, 2, or 3) proteins were measured through quantification of band intensities and normalized against α-actinin (ACTN1) followed by SC controls (red line), and illustrated with bar plots where light gray bars represent no change and dark gray bars display decreased relative protein levels (right panel) (*n =* 3). (**A-2**) Significantly increased RNA levels of CCM2 isoforms in both CCM1-KD and CCM3-KD in HBMVEC cells were observed. The relative transcription expression changes in CCM1, CCM3, and 5 isoforms of CCM2 in CCMs KD HBMVEC cells were measured by RT-qPCR (Fold) and illustrated in the bar plots, where light gray bars represent the relative RNA levels of CCM1, dark gray bars display relative RNA levels of CCM2 isoforms, and black bars for the relative RNA levels of CCM3 (*n =* 3). (**B**) Impacts of mPR-specific actions on the RNA expression of CCM2 isoforms and mPRs in human microvascular endothelial cells. (**B-1**) Under mPR-specific PRG actions (PRG + MIF) for 48 h, enhanced RNA expression levels of most *CCM2* isoforms were observed in both human dermal microvascular endothelial cells (HDMVEC) and human brain microvascular endothelial cells (HBMVEC) cells, while increased RNA expression levels of *CCM1*/*3* were only observed in HBMVECs (*n =* 3). (**B-2**) Significantly increased RNA expression of *PAQR5/7/8* and *PGRMC1* was observed under mPR-specific PRG actions in HBMVECs and human umbilical vein endothelial cells (HUVECs) for 48 h, while only increased RNA expression of *PAQR7/8* was observed in HDMVECs, suggesting RNA expression of most *mPRs* can be dramatically enhanced under mPR-specific PRG actions (*n =* 3). (**C**) Impacts of mPR-specific actions on the protein expression of mPRα (PAQR7) in HDMVECs. After silencing all three *CCM* genes for 48 h, decreased protein expression levels of PAQR7 were observed for all 3 *Ccms*-KD conditions (*n* = 3). (**D**) Impacts of mPR-specific actions on protein expression levels of CCM1/3 in human microvascular endothelial cells (HDMVECs, HUVECs, and HBMVECs) and rat brain microvascular endothelial cells (RBMVECs), compared to mifepristone only (MIF, 20 µM) or vehicle controls (VEH). The relative RNA expression levels were measured through RT-qPCR from at least three different experiments (triplicates per experiment) and normalized to housekeeping gene (ACTB) and scramble control. The relative protein expression levels were measured through quantification of band intensities of targeted proteins by Western blots, subtracted from the surrounding background and normalized against control housekeeping proteins followed by vehicle controls. In all bar plots, the red line is the control baseline for fold change measurements (−/+). ++, +++ above bar indicates *p* ≤ 0.001 for paired *t*-test.

**Figure 2:**
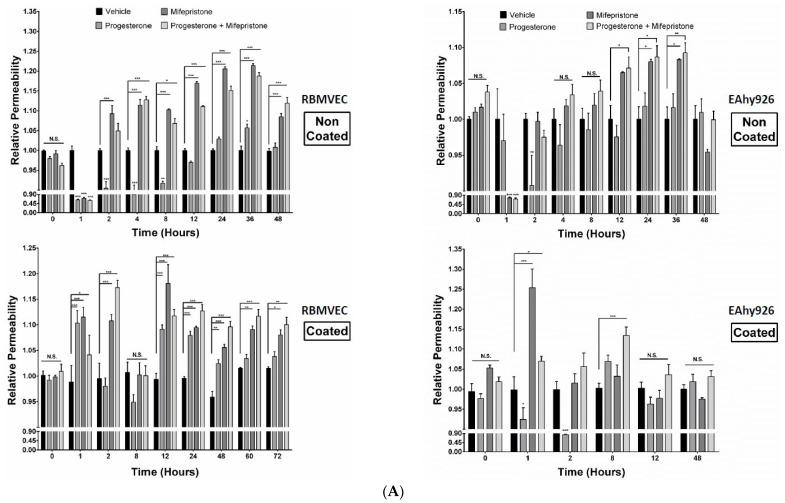

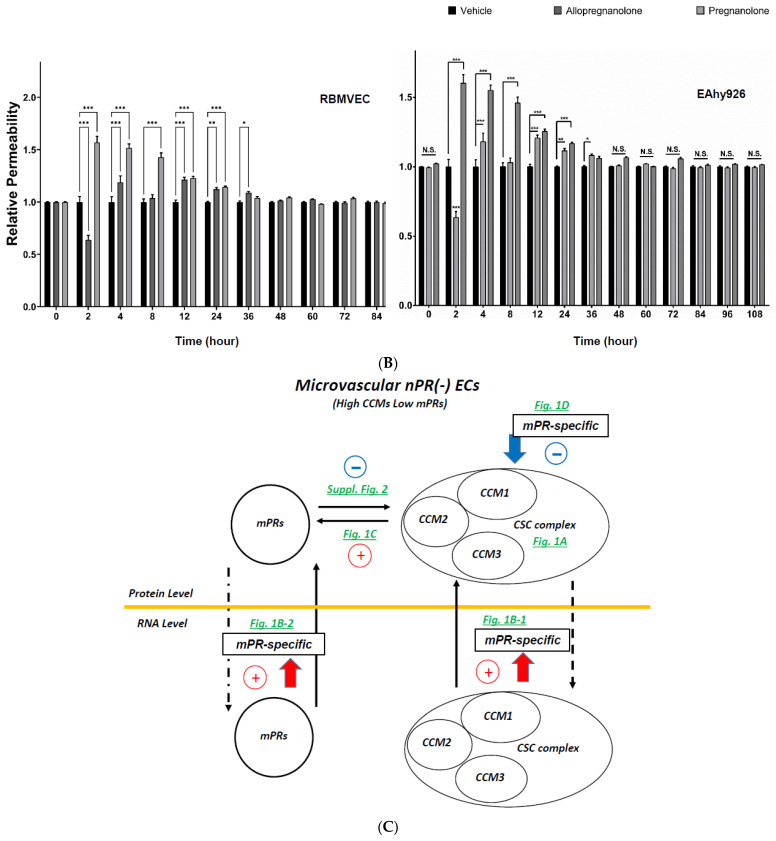
mPR-specific actions on nPR(+/−) ECs increases microvascular permeability in vitro. Two different EC lines, nPR(+) EAhy926 ECs, derived from HUVECs, and nPR(−) RBMVECs, were used to measure in vitro permeability with the passage of FITC-conjugated dextran under vehicle and various steroid treatments. (**A**). *Impact of sex-steroid-induced mPR-specific actions on the permeability of both nPR(+/−) ECs. *Both nPR(+/−) ECs were under sex steroid treatments (PRG (20 µM), MIF (20 µM), and PRG + MIF, (20 µM each)) plated on either uncoated (top panels) or collagen-I coated wells (bottom panels). Although increased levels of permeability were initially observed in both ECs (on Collagen-I coated wells, bottom panels), the permeability of nPR(+) EAhy926 ECs was back to normal after 12 h (bottom right panel), while the permeability was continuously enhanced among all sex hormone treatments for RBMVECs (bottom left panel) on Collagen-I coated wells. Interestingly, permeability remained continuously enhanced among all sex hormone treatments for RBMVECs, when cultured in the absence of collagen-I (upper left panel), while the permeability of nPR(+) EAhy926 ECs did not return to normal until after 48 h (upper right panel), suggesting crosstalk between integrin and PRG-receptors-mediated signaling cascades in nPR(+) EAhy926 ECs, but not in nPR(−) RBMVECs. Four treatments are Vehicle, PRG, MIF, and PRG + MIF sequentially. (**B**). *Impact of neurosteroids-induced mPR-specific actions on the permeability of both nPR(+/−) ECs.*Both nPR(+/−) ECs treated with two common neurosteroids synthesized from PRG (or PRG metabolites), Allopregnanolone (3a-hydroxy-5a-pregnan-20-one, ALLO, 20 µM) and Pregnanolone (3a-hydroxy-5b-pregnan-20-one, P5, 20 µM), were plated on collagen-I coated wells, and the EC permeability was continuously monitored and measured as aforementioned. (**C**). The summarized feedback regulatory mechanism within the CmP signaling network under mPR-specific PRG actions for nPR(−) ECs. Yellow line separates transcriptional and translational levels. The + symbols represent enhancement, and symbols represent inhibition of the expression of targeted genes/proteins. Red-colored symbols/lines represent positive effects of mPR-specific PRG treatment (PRG + MIF), and blue-colored symbols/lines represent negative effects of treatment. Dark-green-colored letters indicate the direct supporting data generated from this work. Arrow indicates effect direction, solid line is the direct impact, and dotted line is indirect effects. The fluorescence intensity of FITC-dextran was measured using a 96-multiwell fluorescent plate reader. In all bar plots, *, **, and *** above any bar graph indicate *p* ≤ 0.05, 0.01, and 0.001, respectively, using two-way ANOVA with Holm–Sidak’s multiple comparison correction.

Similarly, our recent works also demonstrated that CCM2 is the cornerstone for the stability and functionality of the CSC in both breast cancer cells and zebrafish Ccm1/2 mutant strains [16]. These results suggest the existence of a feedback regulatory loop influencing the expression of *CCM2*, based on cellular levels of CCM1 and CCM3 at the translational level [5,7,8,16], which was further supported by data from 293T cells (data not shown). In this report, we examined human brain microvascular endothelial cells (HBMVECs) with identical results (Figure 1(A-1,A-2)), further validating our previous notion that CCM2 is the cornerstone for CSC stability across multiple in vitro and in vivo models.

### 2.2. mPR-Specific Actions on the Performance of the CCM Signaling Complex (CSC) in nPR(−) ECs

The vast majority of vascular ECs are nPR(−) and mPRs(+) (Supplementary Figure S1A–C) and observations of rapid, nonclassic actions of PRG on ECs have been previously reported [17]. Therefore, several relevant nPR(−) microvascular ECs, including human brain microvascular endothelial cells (HBMVECs), human dermal microvascular endothelial cells (HDMVECs), and rat brain microvascular endothelial cells (RBMVECs), were utilized along with a well-known nPR(+) EC line, human umbilical vein endothelial cells (HUVECs), to investigate angiogenic signaling modulated by the CmP signaling network under mPR-specific PRG actions. Interestingly, low protein expression of mPRs/PAQRs was found in these ECs; therefore, no change in PAQR7 protein expression was detected among these EC lines under mPR-specific PRG actions (Supplementary Figure S2). The RNA expression patterns of both *mPRs* and *CCMs* genes were examined under mPR-specific PRG actions demonstrating that increased RNA expression patterns of most *CCM2* isoforms were observed in nPR(−) HBMVECs and HDMVECs (Figure 1(B-1,B-2)). Similarly, increased RNA expression of most *mPRs* genes (Figure 1C) was observed among nPR(−) HBMVECs and HDMVECs, and nPR(+) HUVECs. Likewise, silencing all three *CCM (1, 2,* and *3)* genes led to significantly decreased protein expression of PAQR7 (mPRα) in both nPR(−) HBMVECs and HDMVECs (Figure 1C). Finally, decreased expression levels of CCM1 and CCM3 proteins were observed under mPR-specific PRG actions among nPR(−) HBMVECs, HDMVECs, and RBMVECs as well as nPR(+) HUVECs, reinforcing that PRG works with its agonist, MIF, synergistically to inhibit the protein expression of CCM1/3 (Figure 1D), as seen previously in nPR(+/−) breast cancer cells [5,7,8,16]. However, mPR-specific inhibition of protein expression of CCM1/3 in nPR (+/−) ECs (Figure 1D) is more dramatic than what was observed in either nPR(+/−) breast cancer cells [5,7,8,16], suggesting that mPR-specific PRG actions have a stronger effect on the stability of the CSC in microvascular ECs. Finally, similar to our observations in nPR(+/−) breast cancer cells [5,7,8,16], mPR-specific PRG actions can affect the CSC stability on both transcriptional and translational levels, demonstrating multiple layers of this intricate feedback regulation, where CCM2 plays a key role as the cornerstone for the CSC (Figure 1).

### 2.3. Differential Permeabilities between nPR(+/−) EC Monolayers under mPR-Specific PRG Actions In Vitro

We next wanted to test whether mPR-specific PRG actions would affect the in vitro permeability of two different EC lines, nPR(+) Eahy926 ECs, derived from HUVECs, and nPR(−) RBMVECs. Although increased levels of permeability were initially observed in both ECs (on Collagen-I coated wells (Figure 2A, bottom panels), the permeability of nPR(+) Eahy926 ECs was back to normal after 12 h (Figure 2, bottom right panel), while being continuously enhanced among all sex hormone treatments for nPR(−) RBMVECs (Figure 2A, bottom left panel) on Collagen-I coated wells, indicating that the permeability of nPR(−) ECs is more susceptible to steroid hormones via the CmP axis (Figure 2A, bottom panels) through the CSC-modulated β1-integrin signaling [18,19,20]. Interestingly, permeability remained continuously enhanced among all sex hormone treatments for nPR(−) RBMVECs, when cultured in the absence of collagen-I (Figure 2A, upper left panel), while the permeability of Eahy926 ECs returned to normal after 48 h (Figure 2A, upper right panel), further supporting the important role of β1-integrin-mediated signaling within the CSC-mPRs-PRG-nPRs (CmPn) signaling network in nPR(+) Eahy926 ECs, but not with the CmP signaling network in nPR(−) RBMVECs.

Some common neurosteroids are synthesized from PRG (or named as PRG metabolites), such as allopregnanolone (3a-hydroxy-5a-pregnan-20-one, ALLO) and pregnanolone (3a-hydroxy-5b-pregnan-20-one, P5). Like PRG, neurosteroids can suppress inflammation, reduce apoptosis, and promote neurogenesis and myelination in the nervous system [21], with many potential therapeutic applications [22]. When increased reactive oxygen species production occurs followed by neuroinflammation, levels of several common neurosteroids, such as PRG (P4), ALLO, and P5, can be drastically increased in response to oxidative damage [23], further reaffirming their neuroprotective roles. In fact, ALLO can elicit its marked anxiolytic, antistress, and antidepressant effects at very low (nanomolar) concentrations [24], and the neuroprotective effects of ALLO are mediated through mPRs [21]; therefore, neurosteroids can evoke mPR-specific actions. We then investigated the permeability of two nPR(+/−) ECs (using the early used parameters) under exposure to two aforementioned neurosteroids (ALLO, 20 µM; P5, 20 µM), respectively, in parallel with the vehicle control. The binding affinity data showed that as a neurosteroid, PRG has a 5–100-fold higher affinity to mPRs than its own two metabolites (ALLO and P5) [25], which is reflected by the significantly shortened negative effects of neurosteroids on the permeability of microvascular ECs in general (Figure 2B), compared to mPR-specific sex steroid treatments (Figure 2A, lower panel). These data indicate that neurosteroids also exert their cellular responses via mPR-specific actions, and the duration of steroid-induced mPR actions (BBB perturbation) is determined by their corresponding binding affinities to mPRs, regardless of the existence of **n**PRs (Figure 2B). Therefore, our data indicate neurosteroids also evoke mPR-specific PRG actions (Figure 2B). In sum, our in vitro findings of neurosteroid-induced mPR-specific PRG actions leading to the increased permeability of nPR(−) ECs support a common regulatory mechanism of mPRs-specific actions on the maintenance of the BBB, independent of **n**PRs.

To illustrate the molecular network of this CmP signaling network among ECs, the network was schematically summarized, where key feedback regulatory mechanisms at both the transcriptional and translational levels are illustrated with supporting data from corresponding experiments (Figure 2C). From our illustration, we can see that mPRs-specific PRG actions appear to have a negative effect on the CSC at the proteomic level; however, the CSC also exhibits signs of dual roles in the feedback regulation of certain mPRs’ expression at the same level. From this schematic diagram, it is clearly shown that the transcriptional levels of both *CCMs/mPRs* genes have been enhanced under feedback regulation to compensate for the destabilization of the CmP signaling network under mPR-specific PRG actions at the post-translational level in microvascular ECs (Figure 2C), in concordance with our previous observations in nPR(+/−) breast cancer cell lines [6,7,8].

### 2.4. Subcellular Compartmentation of Key Factors of the CmP Network in nPR(−) RBMVECs under mPR-Specific PRG Actions

In our previous works, we firstly reported that mPRs are nuclear proteins and have abilities for nucleocytoplasmic shuttling in nPR(+/−) breast cancer cells [6,7,8], contradicting their initial roles as membrane-bound proteins. Immunofluorescence (IF) imaging confirmed our previous observations that CCM1 is capable of cytoplasmic-nuclear trafficking [26,27] allowing us to use CCM1 staining as a positive control when measuring expression ratios to assess nuclear/cytosol localization techniques for key CmP players. As predicted, we found that CCM1 is predominantly located in the cytosol, but begins to show early distinct nucleocytoplasmic-shuttling capabilities with definitive nuclear predominance by 24 h under mPR-specific PRG actions, followed by a steady decline back towards the cytosol by 96 h (Supplementary Figure S3A). Similar to CCM1, the majority of CCM3 resides in the cytoplasm initially; however, under mPR-specific PRG actions, CCM3 accumulates in the nucleus starting at 24 h and shuttles back to starting conditions by 72–96 h (Supplementary Figure S3B). Together, these results confirm the nucleocytoplasmic-shuttling capabilities of CCM1 and CCM3 in RBMVECs under mPR-specific PRG actions. Ironically, contrary to their nomenclature, the majority of PAQR8 resides in the nucleus initially, and remains localized primarily to the nucleus throughout the experiment (Supplementary Figure S3C), suggesting a dominant nuclear localization of mPRs in nPR(−) RBMVECs under mPR-specific PRG actions, validating our previous observations in nPR(+/−) breast cancer cells [6,7,8].

Based on these results, we hypothesized that the stability of the CSC in vascular ECs will be the first to be challenged under mPR-specific PRG actions involving the CmP signaling network. These findings translate to the working hypothesis that under mPR-specific PRG actions, leaky vasculatures would be among the first group of anomalies detected in animal models. We then performed subsequent in vivo analysis to test this hypothesis.

### 2.5. Perturbation of the CmP Signaling Network under mPR-Specific PRG Actions Is Sufficient for BBB Disruption in Ccm-Deficient Mice

It has been proven that falling below the threshold of haploinsufficiency for CCMs in microvascular ECs is an essential first step for the pathogenesis of CCM lesions in vivo, generated from both zebrafish [26,28] and mice *Ccms* mutant models [29], while the genetic “two-hit” mechanism is just the most extreme end (null) at the spectrum of haploinsufficiency. Approximately 60% of patients with CCMs have the sporadic form of the disease [30], which is difficult to be explained by the current dominant “two-hit” model [31] even though clonal expansion data provided some degree of support for this “two-hit” model [32]. Therefore, there must be a “trigger” to initiate the hemorrhagic events of CCM lesions which is also suggested by our in vitro data. To further test and validate the effects of mPR-specific PRG actions on the performance and maintenance of permeability of ECs, next, we plan to measure the stability of the BBB in WT and hemizygous *Ccm1*, *Ccm2*, and *Ccm3* mutant mice strains (the same genetic composition as human familial CCM patients) in vivo.

**Figure 3:**
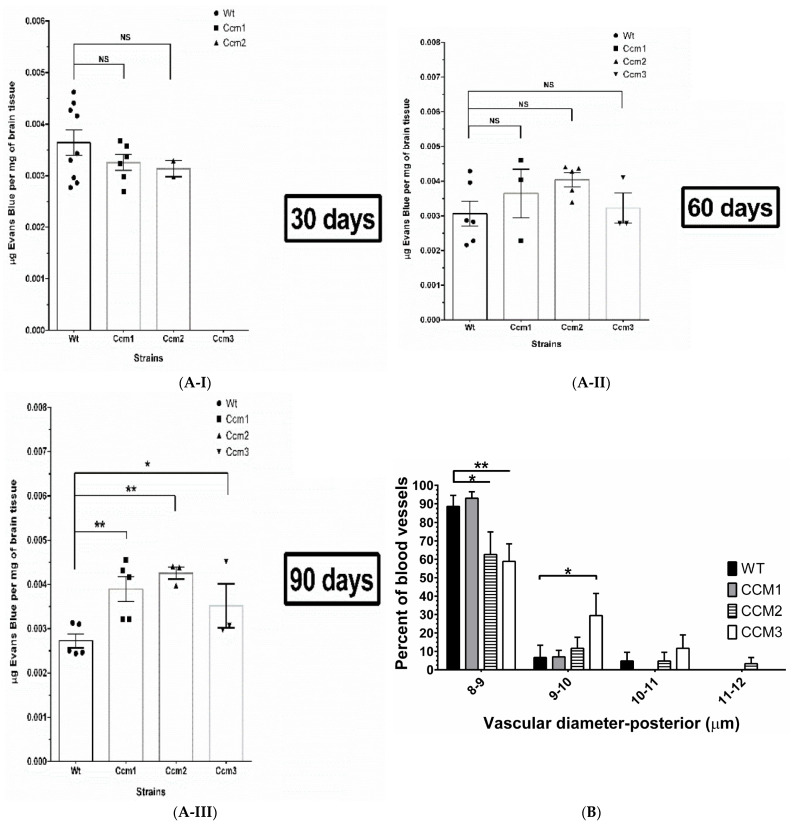

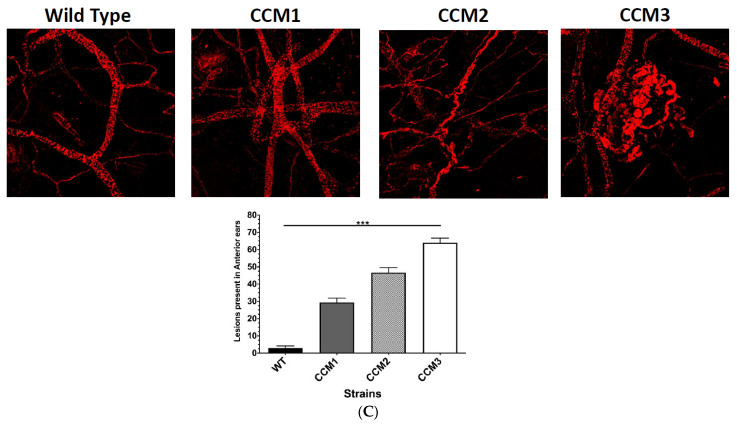
mPR-specific actions on nPR(−) ECs are sufficient for blood–brain barrier (BBB) disruption and formation of subcutaneous lesions in vivo. Hemizygous *Ccms* (1, 2, and 3) mutants and WT (C57 BL/6 J) mice were injected with mPR-specific PRG treatment (a cocktail of PRG + MIF, 100 mg/kg body weight) in peanut oil (vehicle), 5 days a week for 30, 60, and 90 days, respectively. (**A**) BBB permeability was significantly increased only in our 90-day treatment groups for all 3 *Ccm* mutants, demonstrating that increased microvascular permeability in the brain is associated with a combination of chronic exposure to mPR-specific PRG actions and *Ccm* deficiency. (**B**) Subcutaneous vessel diameters in posterior sections of ears were classified into four subgroups based on the range of the vessel size (diameters): group-I (8–9 µM), group-II (9–10 µM), group-III (10–11 µM), and group-IV (11–12 µM). Significantly decreased percentage of vessels was found in *Ccm2*/*Ccm3* within group-I (8–9 µM), compared to WT in the 90-day treatment group, suggesting that more vessels in *Ccm2*/*Ccm3* mutants are distributed in larger diameter groups under mPR-specific PRG actions. Indeed, the significantly increased percentage of larger vessels in *Ccm3* mutant was found in group-II (9–10 µM) compared to WT, and *Ccm2* is the only mutant to display vessels in the largest size group, group-IV (11–12 µM) under mPR-specific PRG actions. (**C**) Subcutaneous vessel lesions in the anterior side of mice ears in all *Ccm* (1, 2, and 3) mutant strains can be visually distinguished in the 90-day treatment groups compared to WT, with *Ccm3* mutant having the largest number of CCM lesions. For Evans blue assays, statistical analysis was generated using one-way ANOVA with either Kruskal–Wallis test or uncorrected Fisher’s LSD test where appropriate. All treated mice, upon completion of the last injection, were injected with Evan’s blue dye (EBD) (500 µG/25 G mouse) which was allowed to circulate for 3 h. Fluorescence data were then measured from the homogenized brain tissue and converted to µg/mL based on standard curves generated in the extraction buffer, and normalized based off the tissue weight (µg Evans blue/mg tissue) followed by controls. In ear tissues, statistical significance was performed using unpaired Student’s *t*-test (*, ** and *** above graphs indicate *p* ≤ 0.05, 0.01, and 0.001, respectively).

Using Evans blue dye (EBD), we quantitatively measured the permeability of blood vessels in the brain which is the major site for CCM pathology and BBB evaluation [33]. To test the presence of compromised permeability of blood vessels in the brain, resulting from vehicle injection, we initially examined EBD data between naïve (untreated with no-injections, N) and vehicle (injection of vehicle, peanut oil) groups among WT, *Ccm1*, and *Ccm2* hemizygous mutant mice; a similar intensity of EBD signals among these control groups was seen (Supplementary Figure S4A), indicating no apparent influence of vehicle injection on the permeability of blood vessels in the brain. We then measured the permeability of blood vessels in the brain among all mouse strains, after treatment with mPR-specific PRG actions for 30, 60, and 90 days, respectively. A similar intensity of EBD signals among *Ccm* (1, 2, and 3) mutants and WT mice for both 30- and 60-day treatment groups was seen, identical to the naïve and vehicle control groups, suggesting no influence of genotypes on the permeability of blood vessels yet (Figure 3(A-I,A-II)). However, significantly increased permeability of the BBB was observed in all *Ccms* mutant mice in the 90-day treatment group compared to WT mice, indicating that the BBB was initially resistant to the disrupting pressure from mPR-specific PRG actions, but eventually collapsed for all *Ccm* mutants due to chronic exposure to mPR-specific PRG actions over a 90-day treatment period (Figure 3(A-III)).

*Gender differences of disrupted permeability within blood vessels under mPR-specific PRG actions*. To better understand the underlying mechanisms of the negative effects of mPR-specific PRG actions on the permeability of blood vessels, WT and *Ccms* mutant mice data were further stratified into male and female groups. Significantly increased BBB permeability was only observed in female *Ccm1* mutants for our 30-day treatment group, suggesting the vulnerability of the BBB to mPR-specific PRG actions in female *Ccm1* mutants (upper right panel, Supplementary Figure S4B). However, increased BBB permeability was only observed in male *Ccm2* mutant mice in our 90-day treatment group (lower left panel, Supplementary Figure S4B), demonstrating that the BBB is able to initially resist the disrupting pressure from mPR-specific PRG actions in males and is more susceptible for disruption earlier in females, as demonstrated by increased leakage in the 30-day treatment group. It must be mentioned that after gender stratification, and our three different treatment groups, our observed results are from a limited sample size, but we anticipate these differences to remain significant with an increased sample size. Overall, EBD data suggest that the BBB integrity and permeability among females is more susceptible to mPR-specific PRG actions.

### 2.6. Perturbation of the CmP Signaling Network under mPR-Specific PRG Actions Leads to Disrupted Angiogenesis in Ccm-Deficient Mice

*Disrupted angiogenesis of blood vessels was further validated in ear vessels*. The negative effects of mPR-specific PRG actions on blood vessels was further extended utilizing a mouse ear angiogenesis assay. Microvessels in mouse ears were evaluated by measuring four different parameters: (1). vascular density, (2). vascular length density, (3). vascular diameter, and (4). number of vessel lesions present in each leaflet. No obvious differences between all four vessel parameters were detected among untreated and vehicle-treated mice, suggesting morphologically equivalent vascular conditions among all untreated/vehicle strains (Supplementary Figure S4C–F). Furthermore, under mPR-specific PRG actions, no differences in vascular density (Supplementary Figure S4C) or vascular length density (Supplementary Figure S4D) were identified between *Ccms* mutants and WT mice, suggesting no defects in these vascular parameters. However, an apparent shift in the distribution of vascular diameters from smaller to larger (Figure 3B) as well as an increased number of vessel lesions (Figure 3C) in *Ccms* mutant mice was observed, compared to WT, indicating vascular defects in these two parameters in *Ccms* hemizygous mice were induced under chronic exposure to mPR-specific PRG actions.

**Figure 4:**
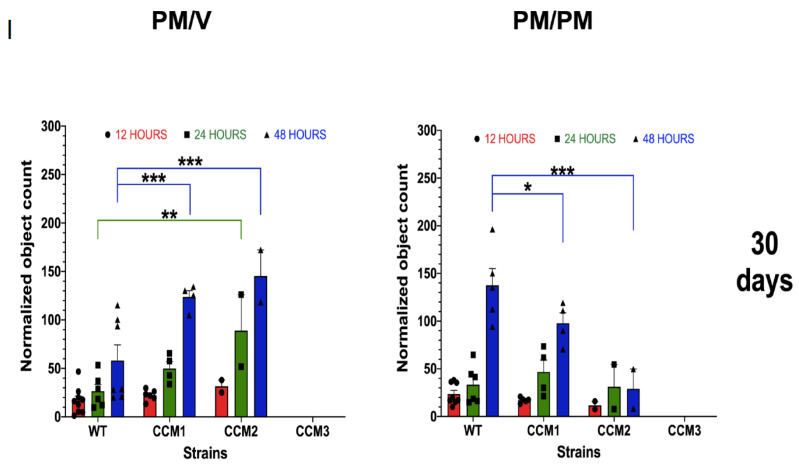

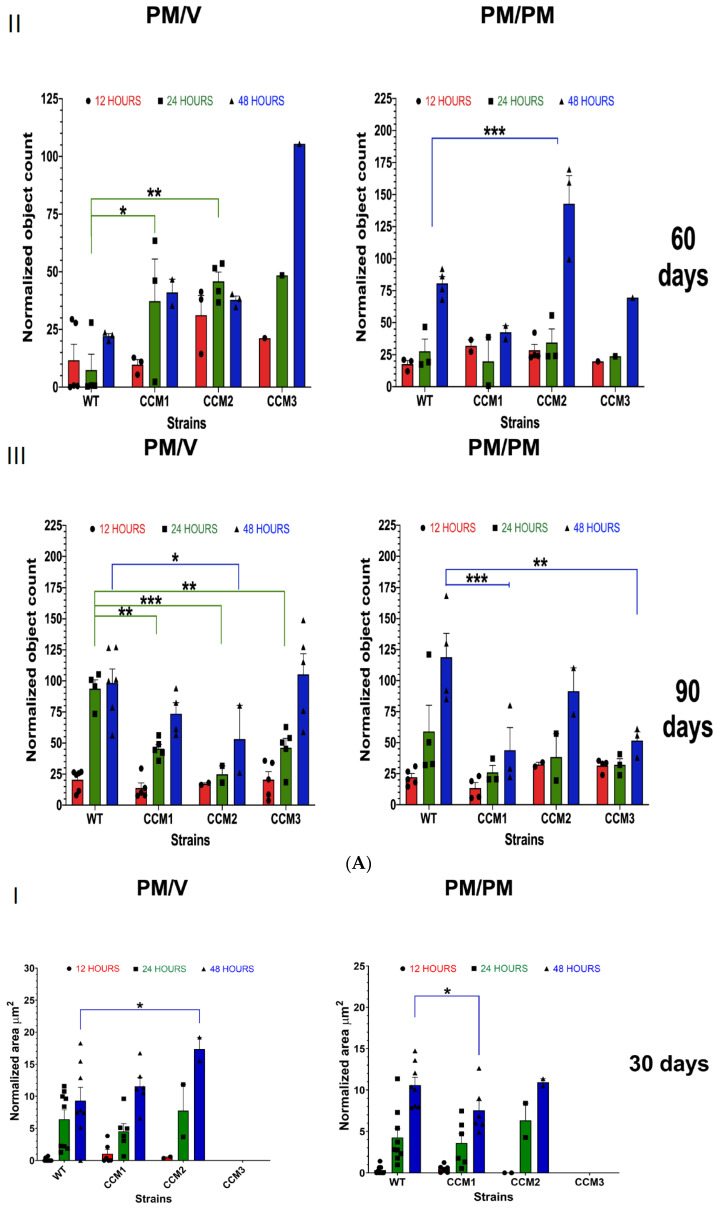

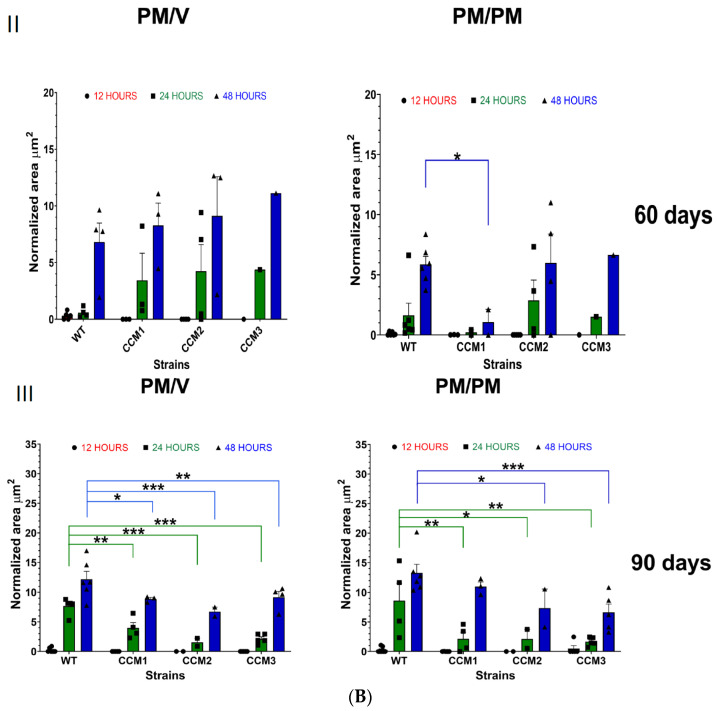
Overall ex vivo angiogenic performance of nPR(−) ECs derived from dorsal aortae of *Ccms* mice under mPR-specific PRG actions. After euthanizing, dorsal aortae were immediately removed from *Ccm*1^+/−^, *Ccm2*^+/−^, and *Ccm3*^+/−^ hemizygous mutant and WT (C57 BL/6 J) mice treated for 30, 60, or 90 days and were divided in half and placed in matrigel media supplemented with vehicle (V, DMSO + EtOH left panels) or mPR-specific PRG treatment (PM, progesterone + mifepristone, 20 µM each, right panels). Images were acquired in 2 h intervals on a Nikon Biostation CT for 72 h. After acquisition, quantification of cell numbers (measured by object counts using the NIKON elements software) was conducted on images taken at 12 (red), 24 (green), and 48 h (blue) timepoints. Quantification of de novo ECs generated from ex vivo angiogenesis (**A**). (**A-I**) *Ccm1* mice displayed significantly increased cell counts at 48 h, while *Ccm2* mice displayed significantly increased cell counts at 24 and 48 h when compared to WT among all 30-day PM in vivo treatment groups in vehicle matrigel media ex vivo (left panel, PM/V); the same 30-day treatment group showed a significant decrease in cell counts at 48 h in *Ccm1* and *Ccm2* mice compared to WT in hormone-supplemented matrigel media ex vivo, (right panel, PM/PM). (**A-II**) There were significant increases in cell counts at 24 h in *Ccm1* and *Ccm2* mice compared to WT among all 60-day treatment groups in vehicle matrigel media ex vivo (left panel), while 60-day treatment only displayed significantly increased counts at 48 h in *Ccm2* mice compared to WT in mPR-specific PRG-supplemented matrigel media ex vivo (right panel). (**A-III**) There was a significant decrease among all 90-day treatment groups in vehicle matrigel media ex vivo at 24 h while only *Ccm2* displayed significantly decreased cell counts at 48 h compared to WT (left panel); the same 90-day treatment group only displayed significant decreases in cell counts at 48 h for *Ccm1* and *Ccm3* strains when compared to WT in mPR-specific PRG-supplemented matrigel media ex vivo (right panel). Overall angiogenic performance of nPR(−) ECs (**B**). After acquisition, analysis of cell growth and migration was measured by area of ECs (using the NIKON elements software) on images taken at 12, 24, and 48 h timepoints. (**B-I**) ECs derived from *Ccm2* mice aortae displayed significant increased growth when compared to WT among all 30-day treatment groups in vehicle matrigel media ex vivo (left panel), while 30-day treatment showed a significant decrease in angiogenesis at 48 h in *Ccm1* mice compared to WT in mPR-specific PRG-supplemented matrigel media ex vivo (right panel). (**B-II**) There were no statistically significant differences among all 60-day treatment groups in vehicle matrigel media ex vivo (left panel), while 60-day treatment showed a significant decrease in angiogenesis at 48 h in *Ccm1* mice compared to WT (same as 30 day) in mPR-specific PRG-supplemented matrigel media ex vivo (right panel). (**B-III**) There was a significant decrease among all 90-day treatment groups in vehicle matrigel media ex vivo at 24 and 48 h compared to WT (left panel), while 90-day treatment also showed significant decreases in angiogenesis at 24 and 48 h (excluding *Ccm1* at 48 h) when compared to WT in mPR-specific PRG-supplemented matrigel media ex vivo (right panel). Cell count data were normalized by dividing cell counts by the length of the aortic vessel (μM). Angiogenesis area measurements (μm^2^) were normalized by dividing growth area by the area of the aortic vessel (μm^2^). Statistical significance was performed using two-way ANOVA (*, **, and *** above graphs indicate *p* ≤ 0.05, 0.01, and 0.001, respectively) and comparisons are color-coded with each time point for clarification. Each symbol represents individual mice samples, which varies by strain (*n =* 1–8).

*Disrupted angiogenesis observed ex vivo.* Ex vivo angiogenesis of aortae isolated from all mice strains under various treatments was analyzed, based on the previous observations that *Ccm1* mutant mice strains demonstrated defective EC functions in the aortae [34]. Quantification of de novo ECs generated from angiogenesis in aortae isolated from the 30-/60-day treatment groups demonstrated that there is a significant increase in aortic ECs in *Ccm1/Ccm2* hemizygous mutant mice, in vehicle matrigel media ex vivo (Figure 4(A-I,A-II) left upper and middle panels, PM/V), demonstrating that angiogenic potential is not yet disrupted. However, a decrease in aortic ECs was observed in *Ccm1*/*Ccm2* hemizygous mutant mice in hormone-supplemented matrigel media ex vivo (Figure 4(A-I) right upper panel, PM/PM), confirming the suppressive roles of mPR-specific PRG actions on angiogenesis in aortic ECs among *Ccm1*/*Ccm2* hemizygous mutant mice. However, in the 60-day treatment group, an increase in ECs was observed at 48 h in *Ccm2* hemizygous mutant mice in hormone-supplemented matrigel media ex vivo (Figure 4(A-II) right middle panel, PM/PM), suggesting the activation of compensation mechanisms due to the essential role of CCM2 in the CSC. More importantly, in the 90-day treatment groups, a significant decrease in ECs in both vehicle (PM/V) and hormone-supplemented (PM/PM) matrigel media ex vivo was observed for all mutant strains at 24 h or CCM1/3 at 48 h, respectively (Figure 4(A-III) bottom left and right panels). In addition, no obvious differences in EC counts were detected among untreated (naïve) and vehicle-treated groups, suggesting uniform angiogenic potential from aortae of all mice strains without mPR-specific PRG pressure in vivo (Supplementary Figure S5A,B all panels). We next sought to evaluate overall angiogenic performance by dynamically measuring the expansion of migrating de novo ECs generated from aortae. In the 30-day group, *Ccm2* hemizygous mutant mice displayed significant increased angiogenic performance in vehicle matrigel media ex vivo (Figure 4(B-I) left upper panel), while a significant decrease in angiogenic performance was observed in *Ccm1* hemizygous mutant mice in hormone-supplemented matrigel media ex vivo for both 30-/60-day treatment groups (Figure 4(B-I,B-II) right upper and middle panels), re-affirming the suppressive roles of mPR-specific PRG actions on angiogenic performance. Interestingly, there were no statistically significant differences among all 60-day treatment groups in vehicle matrigel media ex vivo (Figure 4(B-II) left middle panel). Similar to our previous observations, there was a significant decrease in angiogenic performance among all *Ccm* mutant strains in both vehicle and hormone-supplemented matrigel media ex vivo in the 90-day treatment groups (Figure 4(B-III) left and right bottom panels). Additionally, no obvious differences in angiogenesis were detected among untreated (naïve) and vehicle-treated groups, suggesting uniform angiogenic performance from aortae of all mice strains without exposure to mPR-specific PRG actions in vivo (Supplementary Figure S5C,D, all panels). Finally, we evaluated ex vivo sprouting performance for *Ccm* hemizygous mutants under mPR-specific PRG actions. No obvious differences in sprouting performance were observed among untreated (naïve) and vehicle-treated groups, regardless of ex vivo media used (V/PM), reaffirming relatively uniform angiogenic potential of all mouse strains without hormone pressure in vivo (Supplementary Figure S5E,F, all panels). In the 30-day group, *Ccm1* mice displayed significantly decreased angiogenic sprouting times, regardless of ex vivo media used (Supplementary Figure S5G right and left upper panels). There were no significant differences in angiogenic sprouting times among all 60-day treatment groups (Supplementary Figure S5H right and left middle panels). In the 90-day group, there was a significant increase in sprouting performance among *Ccm3* mice, regardless of ex vivo media used (Supplementary Figure S5I right and left bottom panels). Finally, we evaluated in vitro sprouting performance in RBMVECs under mPR-specific PRG actions. Interestingly, PRG-treated cells displayed an increased trend in sprouting, compared to vehicle controls, but without any significance (Supplementary Table S5). Surprisingly, MIF-treated cells did not display any signs of branching throughout the time course, while mPR-specific PRG actions appeared to rescue MIF inhibition of sprouting, at earlier time points, but no statistical significance was obtained in these comparisons (Supplementary Table S5).

In sum, these results suggest that permanent epigenetic alterations affecting angiogenic signaling cascades, modulated by the CmP signaling network in vivo, occur after 90 days of consistent mPR-specific PRG pressure in hemizygous *Ccm* mutant mice. These altered angiogenic events have been observed to be epigenetically imprinted into microvascular ECs, as demonstrated by our ex vivo angiogenesis data (Figure 4A,B, Supplementary Figure S5A–I) illustrating that disrupted angiogenesis remains even when hormonal stress is removed ex vivo.

### 2.7. Immunosuppression Observed in Ccms Mutant Mice

Since the “gut microbiota-brain axis” is a currently dominant theory for CCM pathological progression toward the disrupted BBB integrity [35], it is highly likely that BBB disruption in *Ccm-*deficient mice (Figure 3) could be the direct results of local inflammatory response induced by prolonged steroid (or vehicle) intraperitoneal injections, through this gut microbiota–brain axis. To examine this possibility, we performed the following experiments to measure the BBB integrity, abdominal distention, and peritoneal inflammation status among different mice (WT and Ccm mutants) after mPR-specific PRG treatments.

*A local inflammatory response is induced by vehicle injections.* Peritoneal inflammation is induced by vehicle (peanut oil) injections, independent of genotype and mPR-specific PRG actions. It has been reported that steroids can influence the gut microbiota and perturbed gut microbiota can affect the brain through the gut microbiota–brain axis [35]. Furthermore, gut microbiota disturbance has been reported to lead to gastrointestinal inflammation [36]. Examination of the mice after mPR-specific PRG treatment revealed abdominal distention, suggesting that peritoneal inflammation may be present. To assess if there was local inflammation in the peritoneal cavity, leukocyte populations were analyzed from this compartment. Peritoneal cells were collected via lavage and stained with fluorescent antibodies to quantify myeloid cell populations. We identified monocytes (CD45.2^+^CD11b^+^ Ly6G^-^Ly6C^high^), neutrophils (CD45.2^+^CD11b^+^ Ly6C^int^Ly6G^high^), large peritoneal macrophages (LPM; CD45.2^+^CD11b^+^ Ly6G^-^Ly6C^-^ F4/80^hi^MHC-II^lo^), and small peritoneal macrophages (SPM; CD45.2^+^CD11b^+^ Ly6G^-^Ly6C^-^F4/80^low^MHC-II^high^) (the FACS gating pipeline is shown in Supplementary Figure S6(A-I,A-II)).

**Figure 5:**
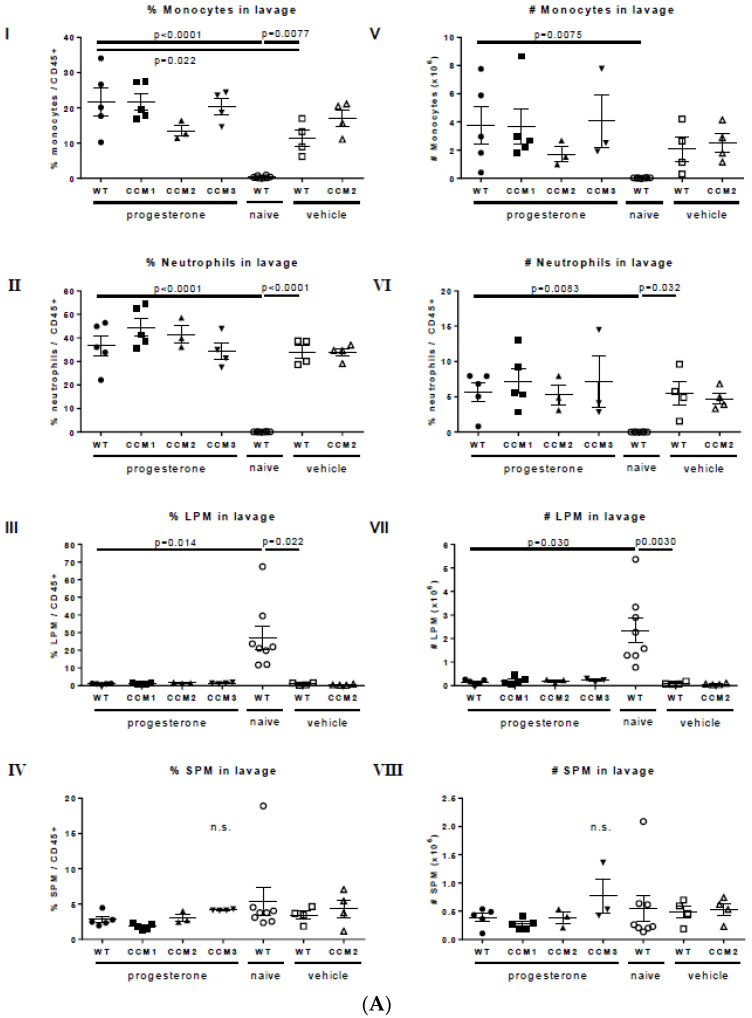

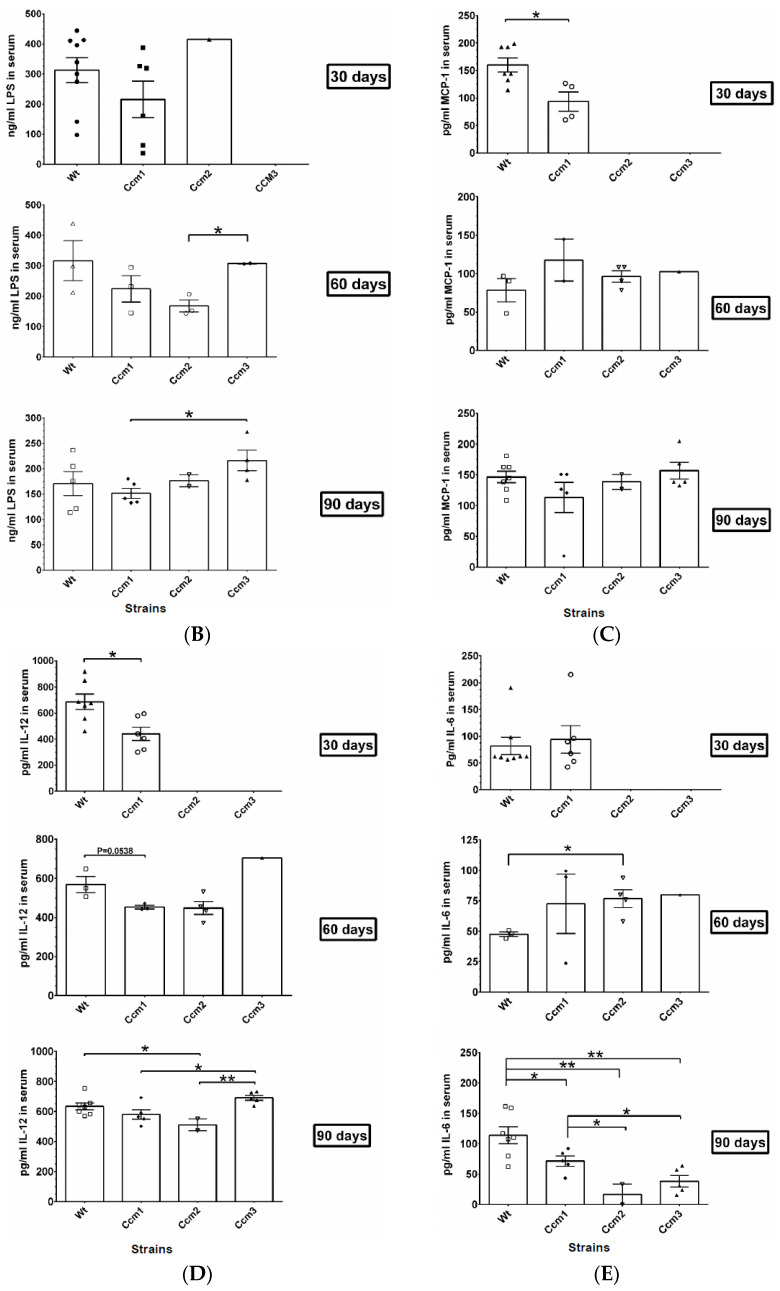
The disrupted BBB is not caused by local inflammatory reactions in *Ccms* mutant mice. (**A**) Monocytes, neutrophils, large peritoneal macrophages (LPM), and small peritoneal macrophages (SPM) within the peritoneal cell populations were collected through peritoneal lavage. Quantification of percentage of (**I**) monocytes (CD45.2 + CD11b+ Ly6G-Ly6Chigh), (**II**) neutrophils (CD45.2 + CD11b+ Ly6CintLy6Ghigh), (**III**) LPM (CD45.2 + CD11b+ Ly6G-Ly6C- F4/80hiMHC-IIlo), and (**IV**) SPM (CD45.2 + CD11b+ Ly6G-Ly6C-F4/80lowMHC-IIhigh) in peritoneal lavage of mice. The percentage of monocytes and neutrophils was calculated relative to CD45+ leukocytes. Quantification of absolute number of (**V**) monocytes (CD45.2 + CD11b+ Ly6G-Ly6Chigh), (**VI**) neutrophils (CD45.2 + CD11b+ Ly6CintLy6Ghigh), (**VII**) LPM (CD45.2 + CD11b+ Ly6G-Ly6C- F4/80hiMHC-IIlo), and (**VIII)** SPM (CD45.2 + CD11b+ Ly6G-Ly6C-F4/80lowMHC-IIhigh) in peritoneal lavage of mice was performed. Dot plots show mean ± SEM (*n =* 3–8). The only significance that was found was with naive wildtype (untreated), which did not receive any injections. This suggests that vehicle (peanut oil) causes a local inflammatory response. (**B**) Lipopolysaccharide-based enzyme-linked immunosorbent assay (LPS-ELISA) was used to measure LPS concentration in mouse serum. Nearly equal amounts of low LPS in the serum of all mouse strains were observed in 30, 60, and 90-day groups with mPR-specific PRG treatment. Relatively higher amounts of nonimmunogenic LPS in *Ccm3* mutant mice were constantly observed from naïve mice to the 90-day treatment group, which also indicates the irrelevance of quantity of nonimmunogenic LPS towards BBB integrity. (**C**) Nearly equal amounts of MCP-1 in the serum of all mice strains were observed in 30, 60, and 90-day treatment groups with mPR-specific PRG treatment but with some notable changes suggesting that MCP-1 may be influenced by either mPR-specific PRG actions or genotypes at early stages in *Ccm1* mutant mice. (**D**) Significantly different amounts of IL-12 in the serum of mice *Ccm* mutant (*Ccm1/2*) strains were observed in 30 and 60 (*Ccm1*) and 90-day groups (*Ccm2*) under mPR-specific PRG treatment, respectively. (**E**) Significantly decreased amounts of IL-6 in the serum of *Ccm* mutant mice strains were observed in the 90-day treatment group under mPR-specific PRG actions. Although higher amounts of IL-6 were observed in *Ccm2* mutants compared to WT, in the 60-day treatment groups (mPR-specific PRG actions), this trend was reversed at 90 days. Statistical significance was performed using unpaired Students *t*-test (*, ** above graphs indicate *p* ≤ 0.05, 0.01, and 0.001, respectively).

We quantified the percentage (Figure 5A(I–IV)) and absolute numbers (Figure 5A(V–VIII)) of monocytes, neutrophils, LPM, and SPM in the peritoneal lavage. Naïve mice exhibited almost no monocytes or neutrophils in their peritoneal cavity (Figure 5A((I,II,V,VI)), attributed to a lack of injections. The majority of myeloid cells present were tissue-resident macrophages, including an abundant LPM population (Figure 5A(III,IV)) and a less abundant SPM population (Figure 5A(VII,VIII)), consistent with prior reports [37]. Relative to naïve controls, WT mice injected with mPR-specific PRG treatment exhibited a significant increase in monocytes (percentage and absolute number) (Figure 5A(I,V)), a significant increase in neutrophils (percentage and absolute number) (Figure 5A(II,VI)), and a significant decrease in LPM (percentage and absolute number) (Figure 5A(III,VII)). These cellular changes are similar to those observed in prior reports of peritoneal inflammation [38]. Relative to naïve controls, WT-vehicle mice exhibited a significant increase in monocytes (percentage only) (Figure 5A(I)), a significant increase in neutrophils (percentage and absolute number) (Figure 5A(II,VI)), and a significant reduction in LPM (percentage and absolute number) (Figure 5A(III,VII)). These data suggest that there is a local inflammatory response to the vehicle. Relative to vehicle controls, mice injected with mPR-specific PRG actions exhibited an increase in the percentage of monocytes in the peritoneal lavage, which was statistically significant (Figure 5A(I)), suggesting that mPR-specific PRG actions may boost monocyte infiltration. However, the absolute number of monocytes and the percentage and number of neutrophils and LPM were similar in the mPR-specific PRG-treated and vehicle groups (Figure 5A(II,III,VI,VII)). SPM were not substantially altered by any treatment (Figure 5A(IV,VIII)).

To assess the possible effects of mouse genotype on this process, leukocyte populations were analyzed in the peritoneal cavity of all mouse strains injected with mPR-specific PRG actions (Figure 5A(I–VIII)). The percentage and number of monocytes (Figure 5A(I,V)), neutrophils (Figure 5A(II,VI)), LPM (Figure 5A(III,VII)), and SPM (Figure 5A(III,VII)) were similar among all genotypes. Vehicle-treated *Ccm* mutant mice exhibited a similar response to vehicle-treated WT mice, suggesting that peanut oil drives peritoneal inflammation, irrespective of either genotype or mPR-specific PRG actions. Overall, these data suggest that mouse CCM genotype does not influence the local inflammatory response observed.

*Disrupted BBB is not associated with nonimmunogenic LPS from H. pylori**bacteria*. Upon arriving, all our mouse strains tested positive for murine norovirus, protozoan *tritrichomonas,* and mostly several strains *of Helicobacter pylori* which usually infect the digestive tract. Possible infection of *H. pylori,* a Gram-negative bacteria, could lead to elevated LPS levels in mouse serum, but is considered to be nonimmunogenic [39]. No obvious differences in LPS levels were detected among untreated (Naïve) and vehicle-treated groups, suggesting the equal existence of low LPS levels in the serum of all mouse strains (Supplementary Figure S6B). No significant differences were observed, compared to WT, in the serum of all mouse strains among 30, 60, and 90-day treatment groups, respectively, suggesting that existing nonimmunogenic LPS in the serum is not influenced by mPR-specific PRG actions (Figure 5B). Relatively higher amounts of nonimmunogenic LPS were observed in *Ccm3* mutant mice compared to treated mutant mice in both the 60- and 90-day treatment groups (Figure 5B), suggesting *Ccm3* mutants may be more susceptible to *H. pylori* bacterial infections.

*Systemic inflammations remain low in mice injected with either peanut oil or mPR-specific PRG treatment.* Infection of murine model with norovirus and *tritrichomonas* allows us to screen for circulating cytokines to evaluate their expression upon mPR-specific PRG treatment. Four key markers of systemic inflammation (TNF-α, MCP-1, IL-12, and IL-6) were detected in the serum from all mice strains.

To assess the possible effects of mouse genotype and injection procedures on inflammatory status, four cytokines were analyzed among all mouse strains under naïve (untreated) and vehicle (peanut oil)-treated conditions. No obvious differences were detected among them (Supplementary Figure S6C–F), suggesting low existing systemic inflammation, irrespective of either genotype, but possibly related to murine norovirus, or protozoan *tritrichomonas* (or peanut oil).

*Immunosuppression was only observed in Ccm deficient mice under mPR-specific PRG actions.* Interestingly, significantly decreased expression of MCP1 was noted in the serum of *Ccm1* mutant mice only in the 30-day treatment group, suggesting possible early immunosuppression due to mPR-specific PRG treatment (Figure 5C upper panel compared to lower panels). IL-12 was also observed to be significantly decreased in the serum of *Ccm1* mutant mice among the 30-day treatment group (Figure 5D upper panel), and although there is a trend of decreased IL-12 in *Ccm1* mutant mice at 60 days (Figure 5D middle panel), the differences are nonsignificant (60-day group *p* = 0.053). There was significantly decreased expression of IL-12 in the serum of *Ccm2* mutant mice among 90-day treatment groups (Figure 5D lower panel), suggesting existing IL-12 cytokines were suppressed, due to the combination of *Ccm* mutant genotypes and mPR-specific PRG actions.

The levels of IL-6 were found to be unaffected in our 30-day treatment groups (Figure 5E upper panel), slightly elevated in our *Ccm2* mutant mice in the 60-day treatment group (Figure 5E middle panel), followed by a dramatic plunge in the serum of all three *Ccms* mutant mice among the 90-day treatment group (Figure 5E lower panel), suggesting IL-6 cytokines were also suppressed, due to the combination of *Ccm* mutant genotypes and chronic mPR-specific PRG actions. These data indicate the suppression of systemic inflammation occurred in all three *Ccms* mutant mice under mPR-specific PRG actions, and confirm that the permeability of blood vessels is not driven by a systemic inflammatory response, but rather is an effect of an interaction between genotype and chronic mPR-specific PRG actions, which also causes immunosuppression. PRG was reported to play a major role in immunomodulatory actions [40], likely through its rapid effects on human T cells [41]. More importantly, it was found that PRG-induced immunosuppression is neither mediated through nPRs [42] nor by the glucocorticoid receptor (GR) [43]. Since peripheral blood monocytes and T cells are nPR(−) [44], nonclassic PRG actions through mPRs are the only viable possibility to explain PRG-induced actions including immunosuppression [45]. Therefore, the amounts of PRG in serum should be determined to investigate whether this immunosuppression is indeed caused by excessive circulating “free form” PRG in serum.

### 2.8. Perturbation of PRG Homeostasis Leads to a Disrupted BBB in Ccm Mutant Mice

*Normal homeostasis of PRG, Serpin A6, and Albumin in the serum of naïve and vehicle-only groups*. Over 98% of PRG in blood is stored and passively transported by plasma proteins [46], mainly through two major PRG-binding proteins in serum, Serpin A6 (binds ~18% PRG) and Albumin (binds ~80% PRG), and is physiologically inactive [47]. To further investigate the underlying mechanisms of sudden elevated levels of active PRG in the serum, quantification of PRG, Serpin A6 (SERPINA), and Albumin in the serum was performed from naïve and vehicle-treated groups. No major significant differences were found between naïve (noninjection) and vehicle-treated groups for all mouse strains (Supplementary Figure S7A–C, with the exception of naïve CCM3 mice in regard to Serpin A6 levels), suggesting total amounts of PRG, Serpin A6, and albumin are normally distributed among WT and *Ccms* mutant strains without hormone pressure.

**Figure 6:**
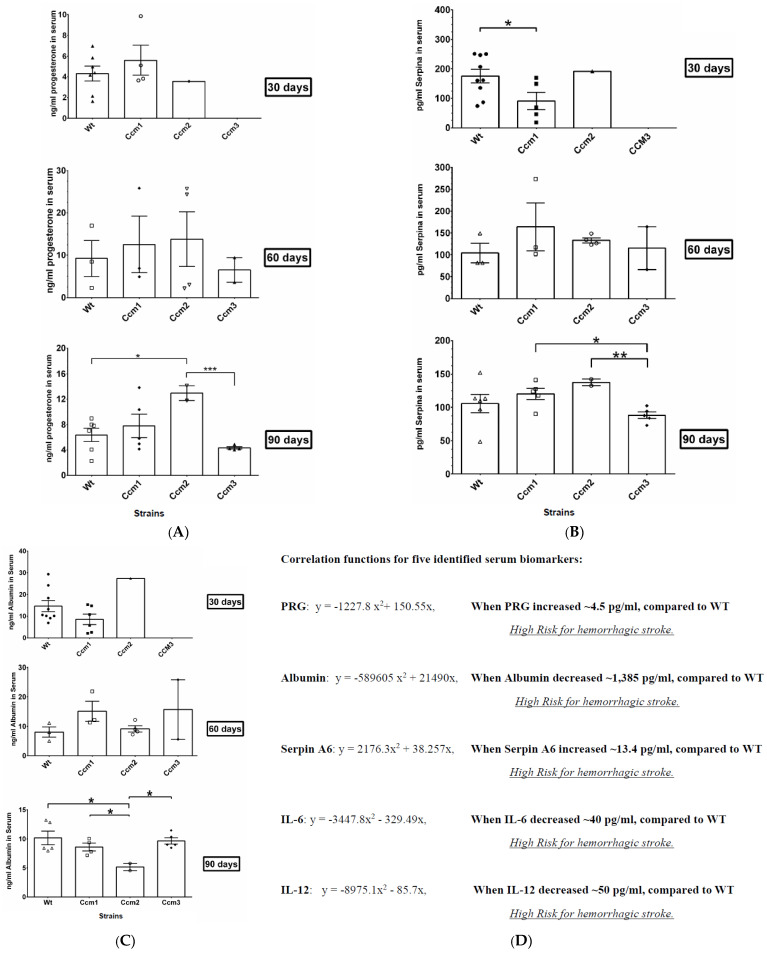
Perturbation of homeostasis of PRG is associated with BBB disruption. (**A**) No obvious differences in PRG levels in mice serum were observed in the 30-day treatment groups (upper panel). Interestingly, free-circulating PRG levels in the mice serum have an overall two-fold increase among all treated genotypes in the 60-day treatment groups (although not statistically significant, middle panel). In the 90-day treatment groups, free-circulating PRG levels regressed to normal ranges; however, free-circulating PRG levels in *Ccm2* mutant mice stayed significantly higher (there was a similar higher trend in *Ccm1* mutant mice as well). (**B**) Significantly decreased SerpinA6 levels were observed in *Ccm1* mutant mice in the 30-day treatment group only, while increased amounts of SerpinA6 were observed in *Ccm*1/2 mutants in both 60- and 90-day treatment groups (although not significant yet). (**C**) Although albumin levels fluctuated in *Ccms* mutants in the 30- and 60-day treatment groups (nonsignificant), significantly decreased amounts of albumin were found in *Ccm2* mutants in the 90-day treatment group (both *Ccm1* and *Ccm3* also shared decreased levels) compared to WT. (**D**) Serum levels of five molecules (PRG, Albumin, SerpinA6, IL-6, and IL-12) were found to correlate with vascular permeability in the BBB (leakage), which can be utilized as etiological biomarkers to predict BBB disruption. The integrated evaluation of these markers will be the key to predict and prevent hemorrhagic stroke. Predictive equations were generated for this figure using binomial regression of the serum levels of the five etiological biomarkers by integrating temporal EBD data in *Ccm* mutant mice under mPR-specific PRG actions (0–3 months exposure). *, ** and *** above graphs indicate *p* ≤ 0.05, 0.01, and 0.001.

*Excessive active PRG in serum of Ccm2 mutant mice in 90-day treatment group.* Quantification of PRG in the serum among all treatment groups was also examined. No significant differences were observed among the different mice strains in the 30- and 60-day treatment groups (Figure 6A upper and middle panels), suggesting homeostasis/biogenesis of PRG is normal among different strains of mice up to two months of mPR-specific PRG actions, irrespective of genotype. The amounts of PRG in serum were found to be elevated significantly in *Ccm2* mutant mice in the 90-day group compared to WT, and the same trend of increased amounts of PRG in serum was also found in *Ccm1* mutant mice but without significance (Figure 6A, bottom panel). These results indicate an abrupt disruption of homeostasis of PRG is correlated with initiation of a disrupted BBB (Figure 3A, bottom left panel) among *Ccm2* mutant mice (and possibly *Ccm1* mice) after chronic exposure to mPR-specific PRG actions. As aforementioned, it must be mentioned that our observed results for *Ccm2* 90-day treatment groups are from a limited sample size due to our strict husbandry standards for eliminating any mice showing abnormal phenotype/behavior, but we anticipate these differences to remain significant with an increased sample size.

### 2.9. Perturbation of Albumin Homeostasis/Biogenesis Is Observed in Ccm-Deficient Mice under mPR-Specific PRG Actions

*Changed expression levels of PRG-binding proteins in serum of Ccm2 mutant mice in 90-day treatment groups.* Significantly decreased levels of Serpin A6 in serum were found for *Ccm1* mutant mice, compared to WT, in the 30-day treatment group (Figure 6B upper panel). However, an increased trend in Serpin A6 in serum was observed in both *Ccm1* and *Ccm2* mutant mice, compared to WT, in the 60- and 90-day treatment groups (although not statistically significant when compared to WT) (Figure 6B middle and lower panels), suggesting that the PRG-binding protein, Serpin A6, follows the same trend in serum level of PRG. These data suggest that the serum level and function of Serpin A6 is normal, irrespective of genotype, and Serpin A6 is upregulated in an effort to balance increased levels of active PRG in the serum (Figure 6A).

Intriguingly, significantly decreased levels of albumin in serum were found in *Ccm2* mutant mice in the 90-day treatment groups despite no differences in either 30- or 60-day treatment groups (Figure 6C all panels), in parallel to PRG trends in the serum, suggesting reduced levels of albumin in serum might contribute to the perturbed homeostasis/biogenesis of PRG (compare Figure 6C and Figure 6A) in *Ccm2* mutant mice. These data also suggest that the CSC is likely involved in the regulation of homeostasis of PRG by modulating the expression levels of its dominant binding protein, albumin, in serum. There was a similar trend between serum levels of albumin and serpin A6 in *Ccm1*, where both binding proteins were decreased in the 30-day treatment group (significant in serpin A6, nonsignificant in albumin), but subsequently were increased in the 60-day treatment group (nonsignificant in both) (Figure 6B,C). This is correlated with the immunosuppression shown previously (Figure S5B–E), further validating the notion that the CSC modulates the biogenesis of albumin, a dominant serum PRG-binding protein (over 80%). Overall, our data indicate that the CSC not only plays a major role in modulating signaling crosstalk between classic and nonclassic PRG receptors [16], but also influences the homeostasis of PRG.

*Biomarkers associated with BBB disruption defined in Ccms mutant mice*. Our data clearly demonstrated that there is a correlation between serum levels of PRG and the initiation of BBB disruption in hemizygous *Ccms* mutant mice. Furthermore, four more biomarkers (SerpinA6, IL-12, IL-6, and Albumin) are all associated with PRG homeostasis in serum, and also correlate well with a disrupted BBB in hemizygous *Ccms* mutant mice. Therefore, we hypothesize that these serum molecules can be used as etiological biomarkers to predict the timing of BBB leakage, the initiating steps in early hemorrhagic events in CCM pathology. We performed binomial regression with the serum concentration of these five biomarkers with the corresponding EBD levels for the same treatment groups temporally (0, 30, 60, and 90 days) to generate binomial correlation functions (Figure 6D). Each biomarker has a unique cutoff value indicating an elevated risk of BBB disruption. These predictive algorithms will be tested, improved, and validated through our ongoing collaborative human CCM biomarker trials.

## 3. Discussion

### 3.1. Perturbed Homeostasis of PRG Is a Casual “Trigger” for BBB Leakage

*Female gender is a key risk factor for hemorrhagic bleeding in CCMs*. Our key findings are that nPR(−) microvascular ECs are more susceptible to the perturbation of the CmP signaling network, which is well documented in clinical appearance. CCMs are more common in women and become symptomatic during their reproductive period (30–40 age range) [48]. Although no conclusive results have been found [49], hormonal changes during pregnancy have long been suggested as significant factors for increased bleeding [14], and female gender is a key risk factor for bleeding in CCM patients [50].

*PRG is a key risk factor for hemorrhagic CCMs*. Spinal hemangiomas have been defined as a pregnancy-related vascular disorder [51], and CCMs account for half of them [52]. Hormonal changes during pregnancy are defined as determining factors in initiating and enhancing the vertebral hemangiomas [51]. Therefore, it has been long speculated that the flux of hormones during pregnancy may predispose CCMs to hemorrhage [14]. A pregnancy-associated increase in PRG levels has been indicated to enhance the progression of vertebral hemangiomas [53], likely through its ability to induce structural changes within the vessel wall [54]. Negative staining with estrogen (EST) but positive staining with PRG in all 12 orbital CCM samples suggested the role of PRG signaling in the pathogenesis of orbital CCMs [55]. Unfortunately, subsequent screening results of CCM lesions from 12 patients showed all negative staining with both EST receptor (ER) and classic nPRs, failing to link both EST- and PRG-mediated signaling to the pathogenesis of CCMs [56]. Finally, it is highly likely that polymorphisms associated with genes other than CCMs, both related with oxidative stress, inflammation, and, in particular, involved in the production and metabolism of sex steroid hormones, such as CYP450 enzymes [57], might significantly contribute in CCM disease onset and progression as additional triggers.

*mPR-specific PRG actions in the CmP signaling network.* Data in nPR(−) blood monocytes and T cells [41], nPR(+) T47D cells [6], and nPR(−) triple-negative breast cancer cells (TNBC) [7,8] indicate that mPRs are quite possibly the only target for mPR-specific PRG actions in nPR(−) cells. The CmP signaling network, which modulates the integrity of the BBB, could be disrupted during pregnancy or physiological/pathological events with excessive circulating “active” PRG in the bloodstream. In this study, we found that the disrupted CmP signaling network is greatly influenced by excessive PRG and the effects are epigenetically imprinted in microvascular ECs, which could subsequently trigger BBB disruption. In the case of hemizygous CCM mutant carriers (human familial CCM patients), there are certain increased risks of hemorrhagic events when exposed to chronically elevated PRG (or MIF) levels in the bloodstream.

Our in vitro data showed that neurosteroids (PRG derivatives) lead to the same phenotype of ECs’ permeability as the mPR-specific PRG actions generated from PRG/MIF, indicating the effect of neurosteroids on ECs is achieved through mPR-specific actions. Mood disorders are common health problems associated with women in their reproductive ages [58], with common symptoms ranging from less severe premenstrual syndrome (PMS) to the more severe premenstrual dysphoric disorder (PMDD) [59]. Although neurosteroids, such as ALLO, can be synthesized de novo in the CNS [60], the majority of neurosteroids (ALLO and P5) are generated as PRG (P4) metabolites in the reproductive system [61]. Interestingly, the correlation between increased ALLO levels and worsened symptoms in PMDD/PMS is very strong [62], although there is an apparent lag time between the ALLO peak and symptom peak [62], suggesting an underlying molecular trigger is needed for increased production of neurosteroids during stress [62]. Data from animal models clearly indicate ALLO and P5 are stress-responsive, serving as homeostatic mechanisms in restoring normal GABAergic function following stress. Acute stress leads to sharp increases in both plasma and CNS concentrations of ALLO to restore GABAergic tone and suppress the acute endocrine stress response, an essential step in homeostatic mechanisms of adaptation to stress, by limiting the extent and duration of reduction in GABAergic inhibitory transmission, and activation of the hypothalamic–pituitary–adrenal (HPA) axis. Disruption in this homeostatic mechanism may lead to the pathogenesis of psychiatric disorders and depression [63]. Intriguingly, accumulated data show that stress can induce or increase the risk of hemorrhagic stroke in both animal models and humans [64], linking high levels of neurosteroids with a high risk of hemorrhagic stroke.

*New paradigm for hemorrhagic progression events in CCMs.* In clinical diagnosis, hemorrhage is often rooted from defective EC junctions, and microvessel rupture is a result of compromised integrity of BBB in CCMs [65]. Many bleeding mechanisms have been proposed in CCMs, of which one major theory is the anticoagulant vascular domain theory (endothelial cofactors that generate anticoagulant APC to induce bleeding) [66]. Our data that enhanced PRG-mPRs signaling due to perturbed homeostasis of PRG leads to BBB disruption, in addition to evidence that long exposure to hormonal contraceptives increases the risks of cerebral venous sinus thrombosis (CVST), a phenotype opposite to hemorrhage [67], seem incongruent with this theory.

The second model is the gut microbiota theory (LPS-activated Toll-like receptor 4 to induce inflammatory-associated hemorrhage) [68] which focuses on the importance of gut microbiota inducing systemic inflammation that promotes detrimental effects on the BBB [69]. However, the main issue with this model is that LPS-induced *Ccm* hemorrhagic mice induce a large CCM burden with massive bleeding, uncharacteristic of human CCMs. Additionally, it has been well documented that immunogenic LPS will compromise vascular function and integrity in multiple organs/tissues [70] including BBB [71], and neuroinflammation [72], irrespective of either genotype or inflammatory response. Finally, this report provides strong evidence that the disrupted BBB observed in our hemizygous *Ccms* mutant mice models is unrelated to the locally induced gut inflammation observed in *Ccms* mutant mice (Figure 5A). Furthermore, our findings instead indicate that immunosuppression caused by mPR-specific PRG actions in *Ccm*-deficient mice is associated with BBB disruption (Figure S3A and Figure 5), which also provides more refuting findings that are in disagreement with the microbiota theory.

Taken together, neither of these two theories addresses a key issue of gender discrepancy in the pathogenesis of CCMs. Female dominance in CCM patients has been long suggested [73], with more severe bleeding and worse neurological outcomes observed in females [74], most likely due to endocrine influences [73]. Therefore, based on the in vitro and in vivo data presented in this report, we propose a new paradigm for the molecular mechanisms initiating BBB disruption, the first step in early hemorrhagic events. In nPR(−) ECs, excessive exposure to mPR-specific PRG actions leads to disruption of the CmP network and enhanced permeability in vitro, while long-term exposure to mPR-specific PRG actions, in vivo, results in BBB disruption in our hemizygous *Ccms* mutant mice models. These findings indicate that the feedback loops within the CmP signaling network are quite sensitive and perturbation of this intricate balance, such as hormone therapy or hormonal contraception regimens, could result in increased risks in BBB disruption, especially for human *CCM* mutant carriers (either genomic or somatic). Our new paradigm provides a theory that is in line with the clinically observed human CCM conditions [5].

### 3.2. Novel Biomarkers Shed Light for the First Time to Predict the Potential Risk of BBB Disruption

Biomarkers have been long sought for early diagnosis of strokes [75], but only with a limited success in ischemic strokes [76,77]. Efforts to define diagnostic biomarkers for hemorrhagic strokes are underway [78]. Inflammatory cytokines have been on target as risk factors or biomarkers for stroke [79]. Circulating levels of TNF-α [80], MCP-1 [81], and IL-6 [82] as biomarkers have all been investigated. Among various cytokines from candidate plasma biomarkers in two small cohorts [83,84], the IL-6 plasma level was lower in hemorrhagic patients compared to stable subjects, which is in accordance with our serum IL-6 data in *Ccm* mutant mice.

Plasma levels of Serpin A6 are nearly 20-fold higher in women relative to men, suggesting gender differences in response to PRG signaling [85]. While no differences were found in women, a significant inverse association between plasma Serpin A6 levels in men was detected, indicating a protective role of Serpin A6 in strokes [85]. Serum albumin levels have been found to be inversely correlated with stroke incidence and outcome [86], but the majority of this work only investigated ischemic strokes [77,87], but a few reports found that serum Albumin levels can predict hemorrhagic stroke with negative correlation [88]. Since the protective role is so significant, high-dose albumin has been shown to be highly neuroprotective during strokes [89], and multicenter clinical trials have been carried out [90], but the neuroprotective function of albumin remains unknown and our data shed light on these possible mechanisms. Ironically, PRG has long been sought as a potential therapeutic drug for post-stroke treatment for both ischemic and hemorrhagic strokes in animal models [91], and now there are even some on-going large clinical trials. The neuroprotective function of PRG has been well defined [92], mainly due to its ability of immunosuppression [93]; therefore, there should be caution in evaluate its potential application as treatment options when current data show the potential role of excessive PRG in inducing BBB disruption. Collectively, our findings reinforce the idea that there are gender- and sex-hormone-associated differences in stroke pathophysiology, and suggest that PRG-mediated signaling should be investigated further as a potential molecular mediator in strokes.

## 4. Materials and Methods

### 4.1. Cell Culture and Treatment, Real-Time Quantitative PCR Analysis (RT-qPCR), and Western Blots

Human brain microvascular endothelial cells (HBMVEC), human dermal microvascular endothelial cells (HDMVEC), human umbilical vein endothelial cells (HUVEC), and rat brain microvascular endothelial cells (RBMVEC) cells were cultured following manufacturers’ recommendations (ATCC) and as previously described [18,19,20,94,95]. Briefly, when cells reached 80% confluency, cells were treated with either vehicle control (ethanol/DMSO, VEH), mifepristone (MIF, 20 µM), progesterone (PRG, 20 µM), mPR-specific PRG actions (PRG + MIF; 20 µM each), or media only (untreated) for steroid treatments. For RNA knockdown experiments, 80% confluent endothelial cells (ECs) were transfected with a set of siRNAs, targeting specific genes, by RNAiMAX (Life Technologies, Carlsbad, CA, USA) as described before [3,6,16]. The expression of *CCM* genes at both the transcriptional and translational levels was confirmed through both RT-qPCR and Western blots (WB) as previously described [3] with detailed information provided (Supplementary Table S1). Briefly, after various treatments, cells were harvested, and RNA expression levels of *CCMs, nPRs* (nuclear progesterone receptors), and *mPRs* (membrane progesterone receptors) genes were determined through RT-qPCR using Power SYBR Green Master Mix with ViiA 7 Real-Time PCR System (Applied Biosystems, Waltham, MA, USA), and data were analyzed with DataAssist (ABI) and Rest 2009 software (Qiagen, Hilden, Germany). All experiments were performed with triplicates as described before [3,6]. The relative expression levels of candidate proteins were measured with WB as described before [3,6,16].

### 4.2. In Vitro EC Permeability Assays

*Growth of nPR(+/−) ECs, under mPR-specific PRG actions, to assess cellular permeability*. First, 2× 10^5^ ECs were plated onto both 6.5 mm Transwell Collagen-coated and noncoated membrane inserts (3 µm pore PTFE, Corning Costar, Cat#3415), and grown for 2 days to form mature monolayers. Permeability was assessed by the passage of FITC-conjugated dextran (relative mw 40,000, Molecular Probes, D1844), added to the media along with hormones. Vascular leakage was determined from the change in average fluorescence intensity in the lower chamber. Before treatment, the medium was replaced with minimal media without FBS (starvation) for 2 h. Then, the medium was replaced with fresh media 1 h before treatment on the top wells. An amount of 2 ul of Thrombin was added to each well (100 ul, 1 U/mL in PBS) for 20 min. An equal volume of media was then removed from the top well before treating with either vehicle control (EtOH, DMSO), or MIF (20 µM), PRG (20 µM), combined sex steroids (PRG/MIF, 20 μM each), allopregnanolone (ALLO, 20 µM), and pregnanolone (P5, 20 µM), all as mPR-specific PRG treatments. An amount of 20 ul of dextran (1 ug/ul in PBS) was then added in the top wells. Time-course (0 (Control vehicle), 2, 4, 8, 12, 24, 36, 48, 60, and 72 h) experiments of various hormone(s) stimulation in nPR(+/−) ECs was measured, respectively. Each sample from the bottom chamber was read on a fluorescent plate reader (excitation wavelength 488 nm), from five independent experiments (*n =* 5 per group). The fluorescence intensity of FITC-dextran in the samples was measured using a 96-multiwell plate reader. Statistical analysis was performed using ordinary two-way ANOVA with Holm–Sidak’s multiple comparison correction as described before [96].

### 4.3. Evaluation of Blood–Brain Barrier with Evan’s Blue Dye (EBD) from Brain Tissues

*Control and treatment groups*: Hemizygous *Ccm*1^+/−^, *Ccm2*^+/−^, *Ccm3*^+/−^ mutants with a C57 BL/6 J background (generous gift of Dr. Marchuk, DUKE) and WT (C57 BL/6 J) mice were injected with an mPR-specific PRG treatment (PRG + MIF, 100 mg/kg body weight) in peanut oil (vehicle), 5 days a week for 30, 60, and 90 days, respectively. Mice were also injected with only peanut oil or left untreated as vehicle and naïve controls, respectively. Upon completion of the last injection, mice were injected intravenously with EBD (500 µG/25 G body weight) and allowed to circulate for 3 h followed by euthanasia and transcardial perfusion with sterile ice-cold 1X PBS; organs were harvested and snap frozen in liquid nitrogen for future use. Individual points on graphs represent a mouse, and sample size, depending on genotype and treatment group, normally ranged from N = 3–20.

*Extraction of EBD from brain tissues*: Tissues were homogenized in 1.5 mL tubes using presterilized pestles in ice-cold PBS. All samples were then loaded on a bead beater machine and agitated at max speed for 1 min and quickly placed back on ice. After centrifugation at 15,000 RPM at 4 °C, supernatants were transferred to a new tube with 1/3 volume of 100% trichloroacetic acid (TCA). Samples were rocked overnight (O/N) at 4 °C and again centrifuged at 15,000 RPM at 4 °C. The supernatant was then transferred to a new tube with 1/3 volume of ethanol before measuring. Each sample was loaded in 8 wells of a 384 well black Costar plate to serve as technical replicates. The plate was then analyzed using a flex station 3 with fluorescence being read using 620 nm excitation and 680 nm emission, with 6 readings per well, auto PMT sensitivity, and column wavelength priority set during the reads.

### 4.4. Immunofluorescent (IF) Staining Preparation for Evaluation of Subcutaneous Microvasculature in the Ears

*Ear collection and preparation*: The procedure was performed as described before [97]. Briefly, whole ears were collected from WT (C57 BL/6 J) and *Ccm*1^+/−^, *Ccm2*^+/−^, and *Ccm3*^+/−^ hemizygous mutant mice after being treated for 90 days with mPR-specific PRG treatment or vehicle control or left untreated as a naïve control. Ears were kept frozen at −80 °C and thawed at 4 °C before preparation. Ears were placed in Hank’s balanced salt solution (HBSS) so that hairs and excess tissue could be removed with scissors under a dissection microscope. Posterior and anterior leaflets of the ears were separated with each leaflet being placed in a separate 24-well plate with 1 mL of 4% paraformaldehyde (PFA) at 4 °C for 15 min. Ears were washed with 1 mL PBS with 0.2% triton X-100 (washing buffer); then, remaining cartilage and hair were removed and washed again before proceeding to staining procedures.

*Preparation of gelatin slides*: A gelatin coating solution was prepared by dissolving 5 g of gelatin in 1 L of heated water; 0.5 g of chromium potassium sulfate was then dissolved in the gelatin. The solution was filtered and applied to slides in a histological dipping tank. Briefly, cleaned slides were placed in a rack and dipped in the gelatin coating solution 3 to 5 times (5 s each). The slides were allowed to dry at room temperature (RT) for 48 h and then stored at 4 °C until ready to use.

*IF staining of ears*: The procedure was performed as described before [97]. Briefly, prepared ears were placed in 24-well plates (all wash/incubation steps were performed in separate wells) with 0.05% (*w/v*) Pronase (antigen retrieval) in PBS with 0.2% triton X-100, and incubated with gentle agitation at RT for 30 min. Ears were washed with wash buffer (1X PBST), followed by blocking buffer (10%BSA + 0.2% triton X-100 (TX-100)), incubated at RT for 30 min, followed with wash buffer. Ears were then incubated with PECAM-1 conjugated antibody (FITC, 488 nm) diluted in blocking buffer to a 1/50 concentration. The plate was covered with aluminum foil and incubated for 1 h at RT. Rhodamine Phallodin conjugated antibody (TRITC, 565 nm) and DAPI (408 nm) diluted to a 1/200 concentration for each were then added into the well without removing PECAM-1 solution and incubated O/N at 4 °C in the dark.

*Mounting and imaging of ears*: Stained ear leaflets were placed in a Petri dish with PBS to prevent drying out during this procedure. Any remaining hairs, cartilage tissue, dust, and fibers remaining after IF staining were removed from the ears under a dissection microscope. The ear leaflets were transferred to a gelatin slide with the inside of the leaflet facing up. Prolong Gold was placed on the ear leaflets to mount. Ears were clamped and allowed to cure in the dark.

The slides were imaged using a Nikon Eclipse Ti confocal microscope using a 40× objective lens with the appropriate lasers for the antibodies used (Supplementary Table S1). A total of 10–20 random images (anterior and posterior) from 3 mice from each strain in the 90-day treatment groups (spanning the full surface area of the ear) were taken for a representative image of the ears depending on the size of the ear using z-stacks. Counts of lesions present for the treatment groups, along with the CCM2 naïve and vehicle controls, were also obtained during this time. The images were saved as an 8-bit, multicolor, black and white file (required for use in the ImageJ software with vessel analysis package) using NIKON Elements software. Briefly, images were first inverted then converted to a bit map with a resolution of 1024 × 1024 pixels with each pixel corresponding to 0.31 uM. The software used for analysis is a vessel analysis plugin (performed automatically in ImageJ) that provides vessel diameter, vessel length density, and vascular density using the above scaling information and produces the averaged results in uM. To summarize the data, we manually created groups (X axis in graphs) based off the measurement values that were obtained for each strain/treatment group. Statistical significance was determined using unpaired *t*-test.

### 4.5. Evaluation of Overall Angiogenic Performance of nPR(−) Aortic ECs from Ccm Mutant Mice Ex Vivo

After euthanizing *Ccm*1^+/−^, *Ccm2*^+/−^, and *Ccm3*^+/−^ hemizygous mutant and WT (C57 BL/6 J) mice treated for 30, 60, or 90 days, the thoracic aorta was carefully dissected, removed, and placed in OPTI-MEM media on ice (viable up to 4 h) [98]. The experiment was initiated by adding 500.0 μL of Matrigel matrix (BD Biosciences) supplemented with vehicle (DMSO + EtOH, V) or mPR-specific PRG treatment (20 μm each, PM) to corresponding 24-well plates; the matrigel media needed to polymerize at 37 °C for 15 min before adding the aortic rings on top. The aortae were cut into equal segments and after addition into the well, another 500.0 μL of corresponding matrigel (V or PM) was added and again allowed to polymerize at 37 °C for 15 min. Finally, 250 μL of EC media was placed, supplemented with either vehicle or mPR-specific PRG treatment (20 μm each, PM) on top of the last matrigel layer. Plates were placed into a Nikon BioStation CT cell culture system to observe ex vivo angiogenesis. Time-specific images of the aortic vessels were taken with pictures acquired every 2 h for a total of 72 h. After acquisition, analysis of cell growth was conducted on images taken at 12, 24, and 48 h. Images (4324 × 4324 pixels) were analyzed using Nikon Elements V5.02 by calibrating images using the dynamic range of the CCD camera (1000 pixels × 1000 pixels) according to specifications from the corresponding Bio station CT objective lens used for image acquisition (0.8 µM/pixel). The area of six independent sections, using Nikon Elements V5.02, was averaged from the images at each corresponding time point based on areas of cell growth that could be visualized to obtain angiogenesis measurements (area of growth-µm^2^). In addition, the area of the aortic vessel was also measured in order to normalize angiogenesis measurements. EC counts were measured using the object count function tool also provided with Nikon Elements with thresholds set to eliminate both high and low extreme values. To normalize cell count values, object count data were divided by the length of the aortic vessel (μM). Sprouting times were estimated using time interval images from the beginning of the experiment until apparent visible sprouting from aortae was observed. All normalized values for images taken at 12 h, 24 h, and 48 h were compared with controls to observe alterations to angiogenic performance and statistical analysis was performed using either *t*-test (sprouting times) or two-way ANOVA using Graphpad Prism.

### 4.6. Measurement of Cytokines and Etiological Serum Biomarkers

*Estimation of monocytes and neutrophils in the peritoneal lavage.* Peritoneal lavage was collected from *Ccms* (1, 2, and 3) mutants and WT mice as previously described [99]. Briefly, after euthanasia, 3 mL PBS was injected into the peritoneal cavity of the mouse. The abdomen was gently massaged for 2 min. Subsequently, lavage fluid was recovered using a pipette. Cells were counted on a hemocytometer slide and 1 × 10^6^ cells were resuspended in staining buffer (PBS with 2% FBS). Staining was carried out as described previously [99]. Briefly, cells were incubated with CD16/CD32 antibody (Fc shield) for 5 min on ice, to prevent nonspecific binding. Subsequently, cells were incubated for 60 min on ice with antibodies against cell surface markers (Supplementary Table S2). Cells were fixed for 5 min on ice with IC fixation buffer (eBiosciences, cat#00-8222-49). Labeled cells were analyzed on a BD FACS CantoII flow cytometer (BD Biosciences) and data were analyzed using FlowJo software (FlowJo). The sample size ranged from N = 3–8, depending on strain and treatment group.

*Measurement of cytokines and other biomarkers in mice serum with ELISA assays*. The serum levels of four serum cytokines, TNF-α, MCP-1, IL-12, and IL-6, as well as PRG and PRG binding proteins (Albumin and Serpin A6), were measured using corresponding ELISA kits (Supplementary Table S2) and quantified using the provided standard titration curve for each kit, following the manufacturer’s instructions.

## 5. Conclusions

In this report, we extended our previous finding of the intricate feedback regulation in the CmP signaling network into nPR(−) ECs. We found that the CSC controls homeostasis of PRG, in the CmP signaling network, linking the perturbed homeostasis of PRG to the center stage of vascular leakage, in vitro, using nPR(−) ECs under mPR-specific PRG actions, as well as the observed BBB disruption in our in vivo *Ccm* mice models under the same treatment conditions. This project provides new insights establishing the existence of and crosstalk within the CmP signaling network, which helps maintain the integrity of the BBB, revolutionizing the current concepts of vascular leakage and BBB disruption, leading to new therapeutic strategies.

## Data Availability

All data generated and/or analyzed during this study are included in this published article (and/or its supplementary files).

## Supplementary Materials

The following supporting information can be downloaded at: https://www.mdpi.com/article/10.3390/ijms23179684/s1.

## Abbreviations

CCMs	Cerebral cavernous malformations
CSC	CCM signaling complex
PRG	Progesterone
MIF	Mifepristone
nPRs	nuclear Progesterone Receptors
mPRs/PAQRs	membrane Progesterone Receptors or Progestin and AdipoQ Receptors
CmP	CSC-mPRs-PRG signaling network
DEGs	Differentially Expressed Genes
DEPs	Differentially Expressed Proteins
BBB	Blood–brain barrier
