# Supplementary material for: mPR-Specific Actions Influence Maintenance of the Blood–Brain Barrier (BBB)
Source: Int J Mol Sci. 2022 Aug 26;23(17):9684. doi: 10.3390/ijms23179684 (PMC9456378; doi:10.3390/ijms23179684)
Supplement: Supplementary file 1 [file ijms-23-09684-s001.zip › ijms-1851059-supplementary.pdf]

**Supplemental information**

mPR-specific actions influence maintenance of the Blood-Brain Barrier (BBB)

Johnathan Abou-Fadel[1], Xiaoting Jiang[1], Akhil Padarti[1], Dinesh Goswami[1], Mark Smith[1]
Brian Grajeda [2], Muaz Bhalli[1], Alexander Le[1], Wendy Walker[1], Jun Zhang[1]*

Department of Molecular and Translational Medicine (MTM) [1]
Texas Tech University Health Science Center El Paso, El Paso, TX 79905 USA
Department of Biological Sciences [2]
University of Texas at El Paso, El Paso, TX 79902 USA

*Running Title: CSC-progesterone signaling in CCM pathogenesis*

*All correspondence:

Jun Zhang, Sc.D., Ph.D.

Department of Molecular and Translational Medicine (MTM)

Texas Tech University Health Science Center El Paso

5001 El Paso Drive, El Paso, El Paso, TX 79905

Tel: (915) 215-4197, Email:

*Supplemental Figures/tables are numbered to closely follow along with the numbering of figures in the main text, although there might be some inconsistent gaps in the numbering of the supplemental figures/tables. We have tried our best to aid in readability.

**Abbreviations:** Cerebral cavernous malformation (CCM), CCM signaling complex (CSC), progesterone (PRG), mifepristone (MIF), progesterone receptors isoform A/B (PR1/2), membrane progesterone receptors (mPRS), progestin and adipoQ receptor (PAQRs),

**Keywords:** Cerebral cavernous malformation, CCM signaling complex (CSC), angiogenesis, CCM2 isoform, progesterone (PRG), mifepristone (MIF), classic nuclear progesterone receptors (PR1/2), non-classic membrane progesterone receptors (mPRS/PAQRs), genomic action, non-genomic actions (rapid actions), RNAseq, Proteomics.

**Suppl. Methodology**

**Immunofluorescent (IF) staining preparation for evaluation of subcellular compartmentation of key players of the CSC-mPRs-PRG (CmP) network in nPR(-) ECs.**

*Growth of RBMVECs using chamber slides for IF applications:* IF staining methods were performed as previously described [29, 163]. Briefly, cells were grown to 80% confluency, then treated with MIF+PRG (20 µM each) in glass chamber slides (Nunc-Lab-Tek II) for the selected time frame, and finally fixed using 4% (*w/v*) PFA. Slides were washed 3X with PBT (0.2% Triton X-100) before antigen retrieval.

*Antigen Retrieval*: Slides were submerged in 10mM sodium citrate buffer ($Na_3C_6H_5O_7$, pH 6.0) containing 0.01% Triton X-100 at 95-98° degrees Celsius (ºC) and were kept in citrate buffer at 95-98ºC for 30 minutes (mins).

*Blocking and Antibody incubation:* Slides were incubated in PBS containing 0.2% Triton X-100 for 10 mins at RT. Cells were blocked with Pierce fast blocking buffer (Fisher) for 2 hours at RT. CCM1-Alexafluor® 488 (1:50), CCM3-Alexafluor® 546 (1:50), and PAQR8 (1:50) antibodies were diluted into PBS buffer containing 2.0% BSA and 0.2% Triton X-100. 200 µl was placed directly in staining chamber to incubate for 2 hrs at RT in the dark. Secondary antibody was added for PAQR8 using mouse anti-rabbit IgG-CFL 555 (1:100). All antibodies used are detailed in Suppl. Table 1.

*Mounting and Imaging for nPR(-) ECs:* DAPI staining occurs during the mounting/sealing process (Suppl. Table 1). Slides should be allowed to mount O/N at 4 ºC in the dark before sealing with nail polish to cure O/N. Imaging was performed using a Nikon Eclipse Ti confocal microscope using a 60X objective lens. Quantification was done automatically using Elements Analysis software provided with the Nikon microscope. Thresholding (used for quantification) was defined and maintained throughout all images to ensure no bias was applied and to exclude low and high outliers. Localization of CCM1/CCM3/PAQR8 was performed using binary operations in Nikon elements to identify the relative ratio of expressed

proteins localizing inside the nucleus (overlaps of DAPI/GFP or DAPI/mCherry signals) compared to expressed proteins in the cytosol (unique GFP or mCherry without overlapping DAPI signal). Fluorescent images are quantified for CCM1 using wavelength channel 488nm while CCM3 and PAQR8 were quantified using wavelength channel 555nm.

**Suppl. table legends:**

**Table S1. Antibodies used in this study**. Detailed information about application, antigen, clone code, secondary antibody, manufacturer, and category number are listed. * IHC, Immunohistochemistry; IF, immunofluorescence; DAB,HRP/DAB staining, WB, Western Blots, FC, Flow Cytometry; Peritoneal Lavage, PL.

**Table S2. ELISA kits used in this study.** Detailed information about application, antigen, manufacturers, and category number are listed.

**Suppl. Table 3: Angiogenic Sprouting Assessment of Rat Brain Microvascular Endothelial Cells (RBMVECs) under steroid treatment.** RBMVECs were treated in triplicates, following manufacturer's instructions, in media containing either vehicle control (EtOH, DMSO), Mifepristone (MIF, 20 μM), progesterone (PRG, 20 μM), or combined steroids (MIF+PRG, 20 μM each) to assess angiogenic sprouting differences. PRG treated cells displayed an increased trend of sprouting, compared to vehicle controls, but without any significance. MIF treated cells did not display any signs of branching throughout the time course, while combined steroid treatment appeared to rescue MIF inhibition of sprouting, at earlier time points, but no statistical significance was obtained in these comparisons. NB= no branching observed. All experiments were performed with triplicates per experiment (n=3). Sprouting angiogenesis was assessed using a modified angiogenesis assay. Briefly, a cell scratch was pressed through the confluent RBMVEC monolayer to mark the starting line. The cells were swept away on one side of that line. Vehicle control (EtOH, DMSO), Mifepristone (MIF, 20 μM), progesterone (PRG, 20 μM), or combined steroids (MIF+PRG, 20 μM each) were added to start the experiments. Sprouting angiogenesis was monitored temporally. The sprouting vessels were visualized using a Nikon Biostation and recorded with a high-resolution digital camera. The migration area was measured from four different fields under 10× magnifications for each condition and time-point. Each experiment was repeated three times. We calculated the averaged sprouting rate (μM/Hrs) according to the equation: RS=(Wi-Wf)/t,

where $W_i$ is the average of the initial width of the angiogenic area (empty space in µM), $W_f$ is the average of the final width of the angiogenic area (initial empty space area-sprouting vessel area, both in µM), and t is the time span of the assay.

**Suppl. Figure legends:**

**Fig. S1. Relative RNA expression profiling of endogenous classic and non-classic PRG receptors and related sex steroid receptors in nPR(+) and nPR(-) cell lines by qPCR**. The expression levels of AR and PR1/2 (**A**), PAQR5/6/7 (**B**), PAQR8/9 and PGRMC1 (**C**) are presented with bar plots, with β-actin run as an inference gene. The relative expression changes of nPRs and mPRs/PAQRs were examined by RT-qPCR (Fold). Abbreviations: HBMVECs: human brain microvascular endothelial cells; HDMVECs: human dermal microvascular endothelial cells; HUVECs: human umbilical vein endothelial cells; hCMEC/D3, immortalized human brain microvascular endothelial cells.

**Fig. S2. Relationship among PAQRs and mPR-specific PRG actions in nPR(-) ECs.** The expression level of PAQR7 protein in nPR(-) EC lines, HBMVEC (human brain microvascular endothelial cells), HDMVEC (human dermal microvascular endothelial cells), HUVEC (human umbilical vein endothelial cells), and RBMVEC (rat brain microvascular endothelial cells), is not influenced by mPR-specific PRG actions. After PRG+MIF treatment (20 µM each) for 48 hrs, no change of relative expression levels of PAQR7 protein was observed among nPR(-) endothelial cell (EC) lines (left panel). The relative expression levels of PAQR7 protein were measured through quantification of band intensities of PAQR7 normalized against α-actinin (ACTN1) followed by vehicle controls (right panel).

**Fig. S3. Relationships and subcellular compartmentation of key players (CCMs and mPRs) within the CmP network in nPR(-) endothelial cells (ECs).** Sub-cellular localization of CCM1, CCM3 and PAQR8 in RBMVECs under mPR-specific PRG actions (MIF+PRG, 20μM each) for 0-96 hrs. **A).** Relative expression and sub-cellular localization of CCM1. Immunofluorescence (IF) approaches revealed modulation in the relative intensity of CCM1 staining in RBMVECs under mPR-specific PRG actions with increased expression observed at 24 hrs as well as a visual shift in localization of CCM1 proteins into the nucleus at 24 hrs but back towards cytoplasm by 72-96 hrs (left and right panels). CCM1 was quantified through ROI intensities and sub-cellular localization changes of CCM1 was confirmed using binary operations to identify the relative ratio of expressed CCM1 that was localized inside the nucleus (overlaps of DAPI/GFP signal) compared to expressed CCM1 that was localized in the cytosol (unique GFP without overlapping DAPI signal). **B).** Relative expression and sub-cellular localization of CCM3. IF approaches revealed no significant modulation in the relative intensity of CCM3 staining in RBMVECs treated with MIF+PRG (left panel) but revealed the majority of CCM3 resides in the cytoplasm initially, with a similar shift in localization of CCM3 proteins into the nucleus at 24 hrs but back towards cytoplasm by 96 hrs, as observed with CCM1 (both panels). CCM3 was quantified through ROI intensities using wavelength channels 555nm and sub-cellular localization of CCM3 was performed using binary operations to identify the relative ratio of expressed CCM3 that was localized inside the nucleus (overlaps of DAPI/mCHERRY) compared to expressed CCM3 that was localized in the cytosol (unique mCHERRY without overlapping DAPI signal). **C).** Relative expression and sub-cellular localization of PAQR8. IF approaches revealed no significant modulation in the relative intensity of PAQR8 staining in RBMVECs treated with MIF+PRG (left panel), but revealed the majority of PAQR8 resides in the nucleus initially, and remains localized primarily to the nucleus throughout the experiment (right panel). PAQR8 was quantified through ROI intensities using wavelength channels 555nm and sub-cellular localization of PAQR8 was quantified using binary operations to identify the relative ratio of expressed PAQR8 that was localized inside the nucleus (overlaps of DAPI/mCHERRY) compared to

expressed PAQR8 that was localized in the cytosol (unique mCHERRY without overlapping DAPI signal). Red line in localization graphs indicate a 1:1 ratio of proteins in nucleus/cytosol. Data were acquired from 8 sets of independent images and normalized against its respective internal controls using 408nm wavelength for DAPI (nuclear) and background staining subtraction. For each replicate, Region of Interest (*ROI*) i*ntensities* were automatically quantified (over 1000 times/per area). In all bar plots, * and ** above any bar graph indicate $P \leq 0.05$ and $P \leq 0.01$, respectively using One-way ANOVA (n=8).

Fig. S4. **Vehicle/Naïve treatment conditions in Ccm deficient mice is not sufficient for BBB disruption or formation of subcutaneous lesions *in-vivo*. A.** Increased microvascular permeability is not caused by either by vehicle (peanut oil) or genotypes. Normal microvascular function in the brain was observed, with no increased microvascular permeability detected in either vehicle control or naïve groups among Ccms (1, 2, 3) mutant and WT mice. Both Ccms (1, 2, 3) mutant and WT (C57BL/6J) mice were injected with Peanut oil 5 days a week for 30 days for vehicle controls, or without any injections set for naïve controls. **B.** EBD data generated in steroid treated groups (Fig. 4A) were further stratified by genders, demonstrating significantly increased BBB permeability was first observed in female Ccm1 mice in 30-days treatment group, while significantly increased BBB permeability was only observed in male Ccm2 mice in 90-days treatment group. **C.** Vascular density of subcutaneous vessels in posterior sections of ears. Subcutaneous vasculatures, another common location for CCM lesions, were also systematically examined. Percent averages of vessels, within the density categories on the x axis, were then pooled across replicates. Subcutaneous vessels were further classified into six subgroups based on the range of the vessel density (counts/view): group-I (7-10), group-II (10-13), group-III (13-16), group-IV (16-19), group-V (19-21), and group-VI (21-24). I). No significant difference was observed among Ccm1, Ccm2, Ccm3 and WT in either I) control or II) treatment; **D.** Vascular length density in posterior sections of ears was determined through calculation of a ratio of skeletonized vasculature area to total area. Percent averages of vessels, within the length density categories on the x axis, were then pooled across replicates. Subcutaneous vessels were further classified into five subgroups based on the range of the vessel length

density (ratio): group-I (0.5-1.0), group-II (1.0-1.5), group-III (1.5-2.0), group-IV (2.0-2.5), and group-V (2.5-3.0). Although increased numbers of Ccm1 mutant was notable in group-III (1.5-2.0), compared to WT, however in general, no significant difference was observed among Ccm1, Ccm2, Ccm3 and WT in either I) control or II) treatment; **E.** Vascular diameter in posterior sections of ears was defined with conversion of pixel color to relative thickness of the vessels in that area. Percent averages of vessels, within the diameter categories on the x axis, were then pooled across replicates. Subcutaneous vessels were further classified into four subgroups based on the range of the vessel size (diameters): group-I (8-9 µM), group-II (9-10 µM), group-III (10-11 µM), and group-IV (11-12 µM); no significant differences was observed among Ccm1, Ccm2, Ccm3 and WT controls; **F.** CCMs lesions of subcutaneous vessels in the anterior side of mouse ears were examined and counted at 40X and any lesion size was counted towards the total numbers for each slide. Ccm2 mutant was chosen to determine the baseline variation between naive (untreated) and vehicle treatment (left panel)**,** no difference was observed between two control groups (right panel) (n=3), indicating no notable influence of vehicle injection on the appearance of vessels (both panels).

**Suppl. Fig** 5. **Overall *ex vivo* angiogenic performance of nPR(-) ECs derived from dorsal aorta's of control mice.** After euthanizing, dorsal aortas were immediately removed from naïve mice (left panels), or Ccms (1, 2, 3) mutants and WT (C57BL/6J) mice treated for 30 days with vehicle (peanut oil, right panels) and were placed in OPTI-MEM media, then divided in half and placed in matrigel media supplemented with vehicle (V, DMSO+EtOH, upper panels) or hormone treatment (PM, progesterone + mifepristone, 20µM each, lower panels). Images were acquired in 2 hr intervals on a Nikon Biostation CT for 72 hrs. After acquisition, quantification of cell numbers (measured by object counts of ECs) was conducted on images taken at 12 (red), 24 (green) and 48hrs (blue) timepoints due to initiation of tube formation of ECs at 48 hrs. Quantification of *de novo* ECs generated from *ex vivo* angiogenesis (A-B). **A.** Neither naïve nor vehicle treated mice displayed any significant differences in cell counts when compared to WT in vehicle supplemented matrigel media *ex vivo* (top panels). **B.** Similarly, neither naïve nor

vehicle treated mice displayed any significant differences in cell counts when compared to WT in hormone supplemented matrigel media *ex vivo* (bottom panels). Overall angiogenic performance of PR(-) ECs (C-D). After acquisition, analysis of cell growth and migration were measured by area of ECs on images taken at 12, 24 and 48hrs timepoints. **C.** Neither naïve nor vehicle treated mice displayed any significant differences in angiogenesis when compared to WT in vehicle supplemented matrigel media *ex vivo* (top panels). **D.** Similarly, neither naïve nor vehicle treated mice displayed any significant differences in angiogenesis when compared to WT in hormone supplemented matrigel media *ex vivo* (bottom panels). Sprouting times of *de novo* ECs generated from *ex vivo* angiogenesis from naïve and vehicle treated mice (E-F). Quantification of sprouting time was performed using time interval pictures from beginning of experiment until apparent visible sprouting from aorta. **E.** Neither naïve nor vehicle treated mice displayed any significant differences in sprouting time when compared to WT in vehicle supplemented matrigel media *ex vivo* (top panels) **F.** Similarly, neither naïve nor vehicle treated mice displayed any significant differences in sprouting time when compared to WT in hormone supplemented matrigel media *ex vivo* (bottom panel). Sprouting times of *de novo* ECs generated from *ex vivo* angiogenesis from Ccms (1, 2, 3) mutants and WT (C57BL/6J) mice treated for 30, 60 or 90-days (**G-I**). **G.** Ccm1 mice displayed significant decreased angiogenic sprouting times in the 30-day treatment groups, regardless of *ex vivo* media used (right and left panels). **H.** There were no significant differences in angiogenic sprouting times among all 60-day treatment groups. **I.** There is a significant increase in angiogenic sprouting times among Ccm3 mice in the 90-day treatment groups, regardless of ex-vivo media used (right and left panels). Cell count data was normalized by dividing cell counts by the length of the aortic vessel (μM). Angiogenesis measurements ($\mu m^2$) were normalized by dividing growth area by the area of the aortic vessel ($\mu m^2$). Statistical significance for cell count and angiogenesis was performed using two-way Anova. Statistical significance for sprouting analysis was performed using unpaired T-test where (*, ** above graphs indicate $P \leq 0.05, 0.01$, respectively). Each symbol represents individual mice samples, which varies by strain (n=1-8).

**Fig. S6. The disrupted brain-blood barrier (BBB) is neither caused by local inflammatory reactions nor associated with systemic inflammations in Ccms mice. A**. Localized inflammations are not caused by either combined steroid (PRG+MIF) action or genotypes. FACS gating strategy to quantify myeloid cells in the peritoneal lavage of mice under various treatments. Monocytes ($CD45.2^{+}CD11b^{+}$ $Ly6G^{-}$ $Ly6C^{high}$), neutrophils ($CD45.2^{+}CD11b^{+}$ $Ly6C^{int}Ly6G^{high}$), large peritoneal macrophages (LPM; $CD45.2^{+}CD11b^{+}$ $Ly6G^{-}Ly6C^{-}$ $F4/80^{hi}MHC\text{-}II^{lo}$) and small peritoneal macrophages (SPM; $CD45.2^{+}CD11b^{+}$ $Ly6G^{-}Ly6C^{-}F4/80^{low}MHC\text{-}II^{high}$) were sorted and quantified. Typical data are shown for I) a naïve WT mouse, and II) a WT mouse injected with combined steroid (PRG+MIF) treatment. **B.** Systemic inflammations are not associated with non-immunogenic LPS from *H. pylori* bacteria in Ccms mutant mice**.** Systemic inflammations are low and could be suppressed with Ccms mutant genotypes. The disrupted brain-blood barrier (BBB) is not associated with non-immunogenic LPS from H*elicobacter* pylori. Lipopolysaccharide-Based Enzyme-Linked Immunosorbent Assay (LPS-ELISA) was used to measure LPS concentration in mouse serums. LPS concentrations were measured and quantified by ELISA in WT, Ccm1, Ccm2, Ccm3 mutant mice under naïve (untreated) and Vehicle [vehicle (peanut oil)-treated] conditions. No obvious difference was detected among Naïve-WT, Vehicle-WT, Naïve-Ccm1, Naïve-Ccm2, Vehicle-Ccm2, and Naïve -Ccm3, suggesting similar existence of low LPS levels in the serum of all mouse strains. **C**. The serum levels of inflammatory cytokine, TNF-α, is low and not influenced by either steroid actions or genotypes. Nearly equal amounts of TNF-α in the serum of all mouse strains were also observed in 30, 60, and 90-day treatment groups with PRG+MIF, suggesting that TNF-α is not influenced by either steroid actions or genotypes. I). No obvious differences for TNF-α were detected among Naïve-WT, Vehicle-WT, Naïve-Ccm1, Naïve-Ccm2, Vehicle-Ccm2, and Naïve-Ccm3, suggesting similar existence of low TNF-α in the serum of all mouse strains. II). Nearly equal amounts of low TNF-α in the serum of all mouse strains were also observed in 30, 60, and 90-day treatment groups of combined steroid (PRG+MIF) treatment, suggesting that low amounts of MCP-1 is not caused by either combined steroid (PRG+MIF) actions or genotypes. A possible immune suppression was observed in Ccm1 at 30-day treatment group. **D.** The serum level of inflammatory cytokine, MCP-1, is low and not

caused by either combined steroid (PRG+MIF) actions or genotypes. The concentrations of the cytokine, MCP-1, were measured and quantified by ELISA in naïve (untreated) and Vehicle [vehicle (peanut oil)-treated] conditions. No obvious differences for MCP-1 were detected among Naïve-WT, Vehicle-WT, Naïve-Ccm1, Naïve-Ccm2, Vehicle-Ccm2, and Naïve-Ccm3, suggesting equal existence of low MCP-1 in the serum of all mouse strains**. E.** The concentrations of the cytokine, IL-12, were measured and quantified by ELISA in naïve (untreated) and vehicle [vehicle (peanut oil)-treated] conditions. No obvious differences for IL-12 were detected among Naïve-WT, Vehicle-WT, Naïve-Ccm1, Naïve-Ccm2, Vehicle-Ccm2, and Naïve-Ccm3, suggesting equal existence of low IL-12 in the serum of all mouse strains. **F.** The concentrations of the cytokine, IL-6, were measured and quantified by ELISA in naïve (untreated) and vehicle [vehicle (peanut oil)-treated] conditions. There is decreased IL-6 in Naïve-Ccm2 mice compared to Naïve-WT. No other obvious differences for IL-6 were detected among Naïve-WT, Vehicle-WT, Naïve-Ccm1, Vehicle-Ccm2, and Naïve-Ccm3, suggesting mostly similar existence of low IL-6 in the serum of most mouse strains.

**Fig. S7. Perturbation of homeostasis/biogenesis of PRG associated with initial hemorrhagic events in the pathogenesis of CCMs. A.** The concentrations of progesterone (PRG) in mouse serum were measured and quantified by ELISA in naïve (untreated) and vehicle [vehicle (peanut oil)-treated] conditions. No quantitative difference of PRG in mouse serum was detected among naïve (untreated) and vehicle [vehicle (peanut oil)-treated] groups, suggesting equal existence of low PRG in the serum of all control mouse strains. **B.** The concentrations of Serpin A6 in mouse serum were measured and quantified by ELISA in naïve (untreated) and vehicle [vehicle (peanut oil)-treated] conditions. Mostly no quantitative difference of Serpin A6 in mouse serum was detected among naïve (untreated) and vehicle [vehicle (peanut oil)-treated] groups, suggesting mostly similar existence of Serpin A6 in the serum of all mouse strains. Naïve-Ccm3 showed increased levels of Serpin A6 compared to Naïve-WT, possible due to low sample size of naïve-Ccm3 (n=2) **C.** The concentrations of albumin in mouse serum were measured and quantified by ELISA in naïve (untreated) and vehicle [vehicle (peanut oil)-treated] conditions. No

quantitative difference of albumin in mouse serum was detected among naïve (untreated) and vehicle [vehicle (peanut oil)-treated] groups, suggesting equal existence of albumin in the serum of all mouse strains.

**Suppl. Table 1**

| Application | Antigen | Gene | Clone | Secondary | Vendor | Cat # |
|---|---|---|---|---|---|---|
| | | | | | | |
| IF/WB | CCM1 | Krit1 | E-8 | (anti-mouse) | Santa Cruz | sc-514371 |
| IF/WB | CCM1-AF488 | Krit1 | E-8 | (anti-mouse) | Santa Cruz | sc-514371 |
| IF/WB | CCM1 | Krit1 | 8-RY2 | (anti-mouse) | Santa Cruz | sc-134376 |
| IF/WB | CCM1 | Krit1 | | (anti-rabbit) | OriGene | AP26021PU-L |
| IF/WB | CCM3 | PDCD10 | C-8 | (anti-mouse) | Santa Cruz | sc-365586 |
| IF/WB | CCM3-AF647 | PDCD10 | C-8 | (anti-mouse) | Santa Cruz | sc-365586 |
| IF/WB | CCM3-AF546 | PDCD10 | C-8 | (anti-mouse) | Santa Cruz | sc-365586 |
| IF/WB | CCM3 | PDCD10 | F-12 | (anti-mouse) | Santa Cruz | sc-365587 |
| IF/WB | c-Myc-FITC | | 9E/10 | (anti-mouse) | Santa Cruz | sc-40 |
| IF/WB | mouse anti-rabbit IgG-CFL 488 | | | secondary | Santa Cruz | sc-516248 |
| WB | PAQR5 | mPRγ | | (Anti-Rabbit) | Aviva | OASG04642 |
| WB | PAQR5/6 | mPRδ/γ | B-8 | (anti-mouse) | Santa Cruz | sc-514273 |
| WB | PAQR7 | mPRα | | (Anti-Rabbit) | Aviva | OASG04641 |
| WB | PAQR7 | mPRα | | (Anti-Rabbit) | Aviva | ARP67727 |
| WB | GAPDH | | 411 | (anti-mouse) | Santa Cruz | sc-47724 |
| WB | c-Myc | | 9E/10 | (anti-mouse) | Santa Cruz | sc-40 |
| WB | PAQR8 | mPRβ | | (Anti-Rabbit) | Aviva | ARP66903 |
| WB | β-actin | | C-2 | (anti-mouse) | Santa Cruz | sc-8432 |
| WB | α-actinin | | H-2 | (anti-mouse) | Santa Cruz | sc-17829 |
| WB | α-actinin-AF488 | | H-2 | | Santa Cruz | sc-17829 |
| WB | Rabbit-HRP | | | | Santa Cruz | sc-2357 |
| WB | Mouse-HRP | | m-IgGκ BP | | Santa Cruz | sc-516102 |
| WB | m-IgGκ BP IgG-CFL 488 | | m-IgGκ BP | | Santa Cruz | sc-516176 |
| IHC/IF | DAPI | | | | Santa Cruz | sc-3598 |
| IHC/IF | PECAM-1 -AF488 | | MEC 13.3 | | Santa Cruz | sc-18916 |
| IHC/IF | PECAM-1 -AF594 | | MEC 13.3 | | Santa Cruz | sc-18916 |
| IHC/IF | Ep-CAM | | C-10 | | Santa Cruz | sc-25308 |
| IHC/IF | α-Actin | | 1A4 | | Santa Cruz | sc-32251 |
| IHC/IF | α-Actin | | | | ThermoFisher | MA1-06110 |
| IHC/IF | Desmin | | RD301 | | Santa Cruz | sc-23879 |
| IHC/IF | Desmin | | | | ThermoFisher | PA5-16705 |
| IF | mouse anti-rabbit IgG-CFL 555 | | | secondary | Santa Cruz | sc-516249 |
| IF | PAQR8 | mPRβ | | (Anti-Rabbit) | Aviva | OAAB11180 |
| FC/PL | CD16/CD32 | Fc Shield | 2.4G2 | | Tonbo Biosciences | 70-0161-U500 |
| FC/PL | CD45.2 -APC | | 104 | | eBiosciences | 17-0454-81 |
| FC/PL | CD11b-PerCP | | M1/70 | | Biolegend | 101229 |
| FC/PL | MHC class II (I-A/I-E)-FITC | | M5/114.15.2 | | Invitrogen | 11-5321-82 |
| FC/PL | Ly6G-PE | | 1A8 | | BD Biosciences; | 561104 |
| FC/PL | Ly6C-PB | | HK1.4 | | Biolegend | 128013 |
| FC/PL | F4/80 PE-Cy7 | | BM8 | | eBiosciences | 25-4801-82 |

**Suppl. Table 2**

| Gene | Cytokine elisa Kits | Supplier | Catalog # |
|---|---|---|---|
| IL6 | BD OptEIA Mouse IL-6 ELISA Set | BD biosciences | 555240 |
| IL12 | BD OptEIA Mouse IL-12 (p40) ELISA Set | BD biosciences | 555165 |
| MCP-1 | BD OptEIA Mouse MCP-1 ELISA Set | BD biosciences | 555260 |
| TNF-a | BD OptEIA Mouse TNF (mono/mono) ELISA Set | BD biosciences | 555268 |
| Progesterone (PRG) | Progesterone ELISA | DRG international | EIA-1561 |
| Lipopolysaccharides (LPS) | Mouse Lipopolysaccharides (LPS) ELISA Kit | MyBioSource | MBS7700668 |
| Serum Albumin | Mouse Serum albumin ELISA Kit | EIAAB Science | E1028m |
| SERPINA6 (CBG) | Mouse Corticosteroid-binding globulin (SERPINA6) ELISA Kit | abbkine | KTE70454 |

**Suppl. Table 3**

| HBMVEC Angiogenesis Branching assay | | | | |
|---|---|---|---|---|
| **Time (Hrs)** | **VEH Sprouting Rate (µM/Hrs)** | **PRG Sprouting Rate (µM/Hrs)** | **MIF Sprouting Rate (µM/Hrs)** | **PRG+MIF Sprouting Rate (µM/Hrs)** |
| **12** | 0.809 ±0.09 | 2.273 ±0.77 (P=0.055) | NB | 0.483 ±0.34 |
| **24** | 0.312 ±0.11 | 1.267 ±0.57 (p=0.08) | NB | 0.175 ±1.39 |
| **48** | NB | 0.547 ±0.27 | NB | NB |
| **72** | NB | 0.525 ±0.19 | NB | NB |
| **96** | NB | 0.476 ±0.26 | NB | NB |

Fig. S1A

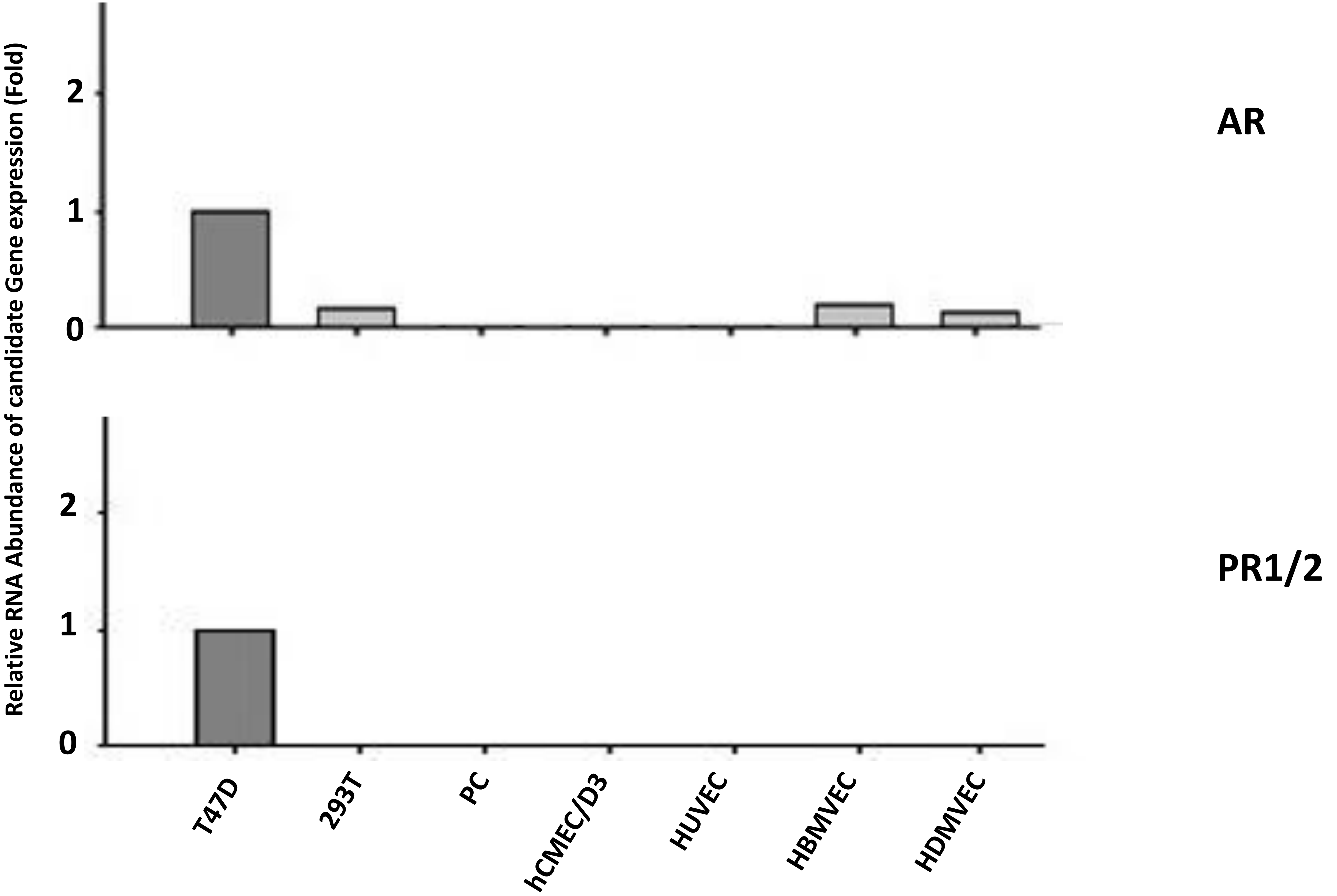

Relative RNA Abundance of candidate Gene expression (Fold)
2
1
0
AR
2
1
0
PR1/2
T47D
293T
PC
hCMEC/D3
HUVEC
HBMVEC
HDMVEC

Fig. S1B

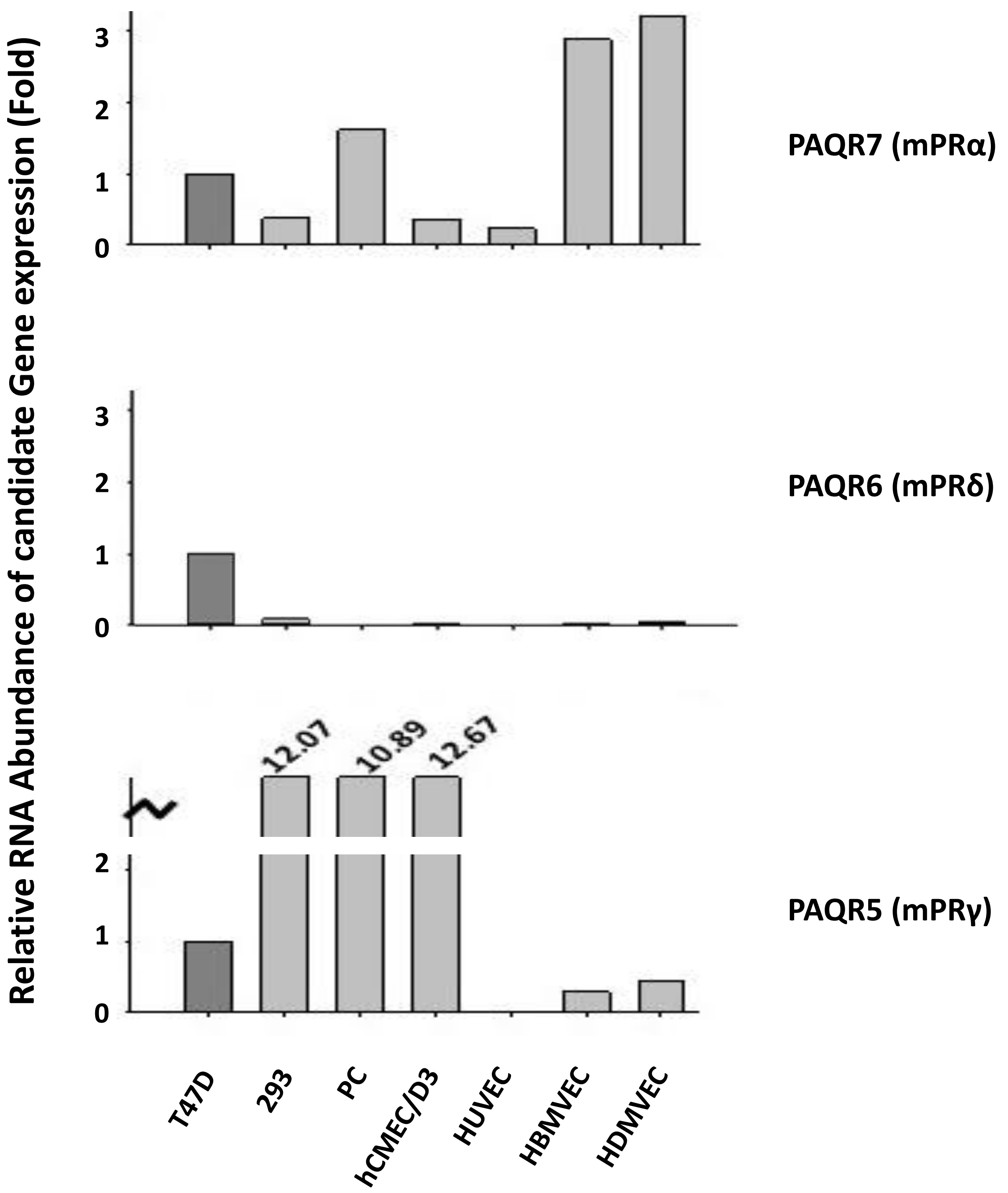

Relative RNA Abundance of candidate Gene expression (Fold)
3
2
1
0
PAQR7 (mPRα)
3
2
1
0
PAQR6 (mPRδ)
12.07
10.89
12.67
2
1
0
PAQR5 (mPRγ)
T47D
293
PC
hCMEC/D3
HUVEC
HBMVEC
HDMVEC

Fig. S1C

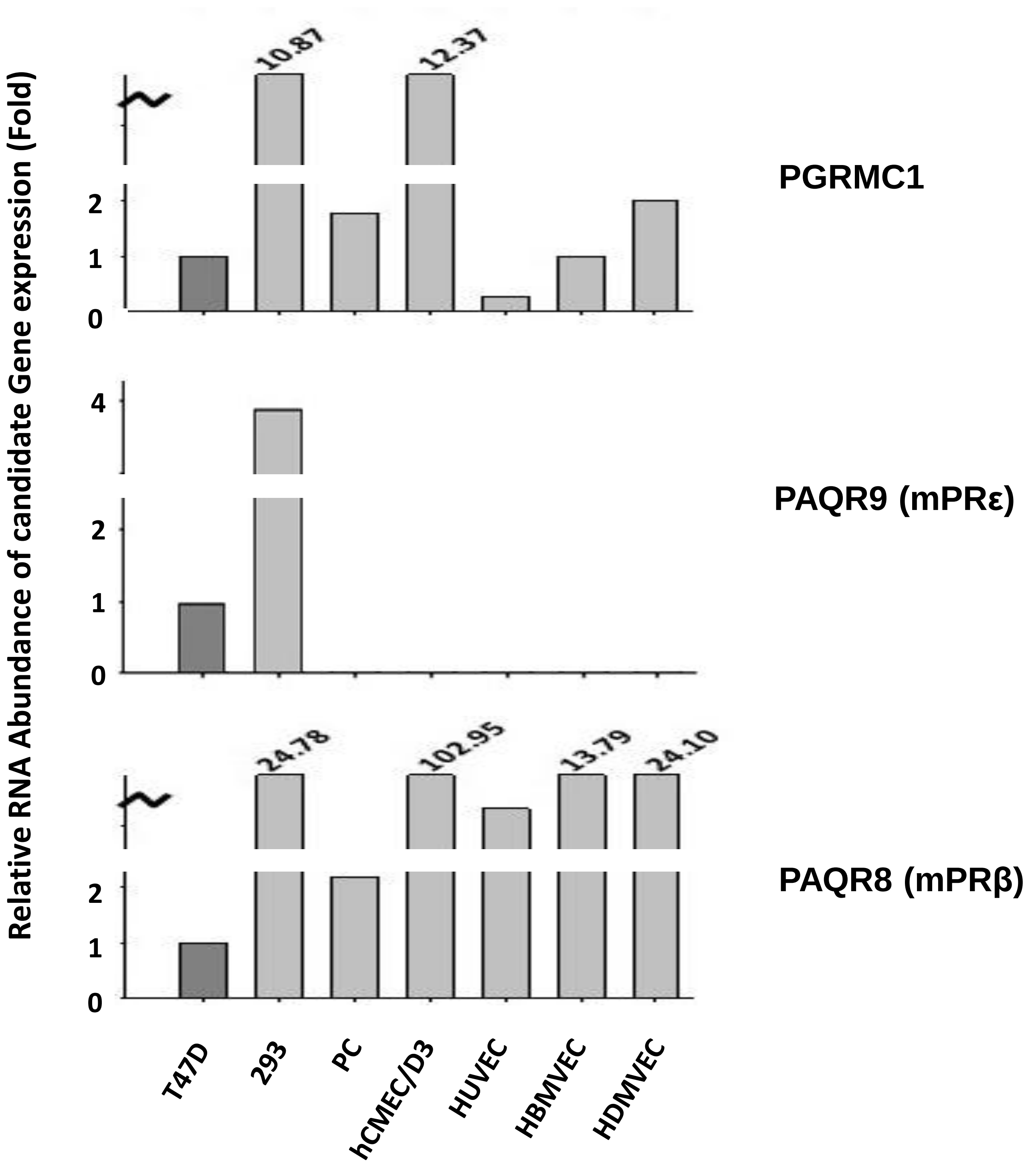

PGRMC1
PAQR9 (mPRε)
PAQR8 (mPRβ)
Relative RNA Abundance of candidate Gene expression (Fold)
10.87
12.37
24.78
102.95
13.79
24.10
0
1
2
4
T47D
293
PC
hCMEC/D3
HUVEC
HBMVEC
HDMVEC

Fig. S2

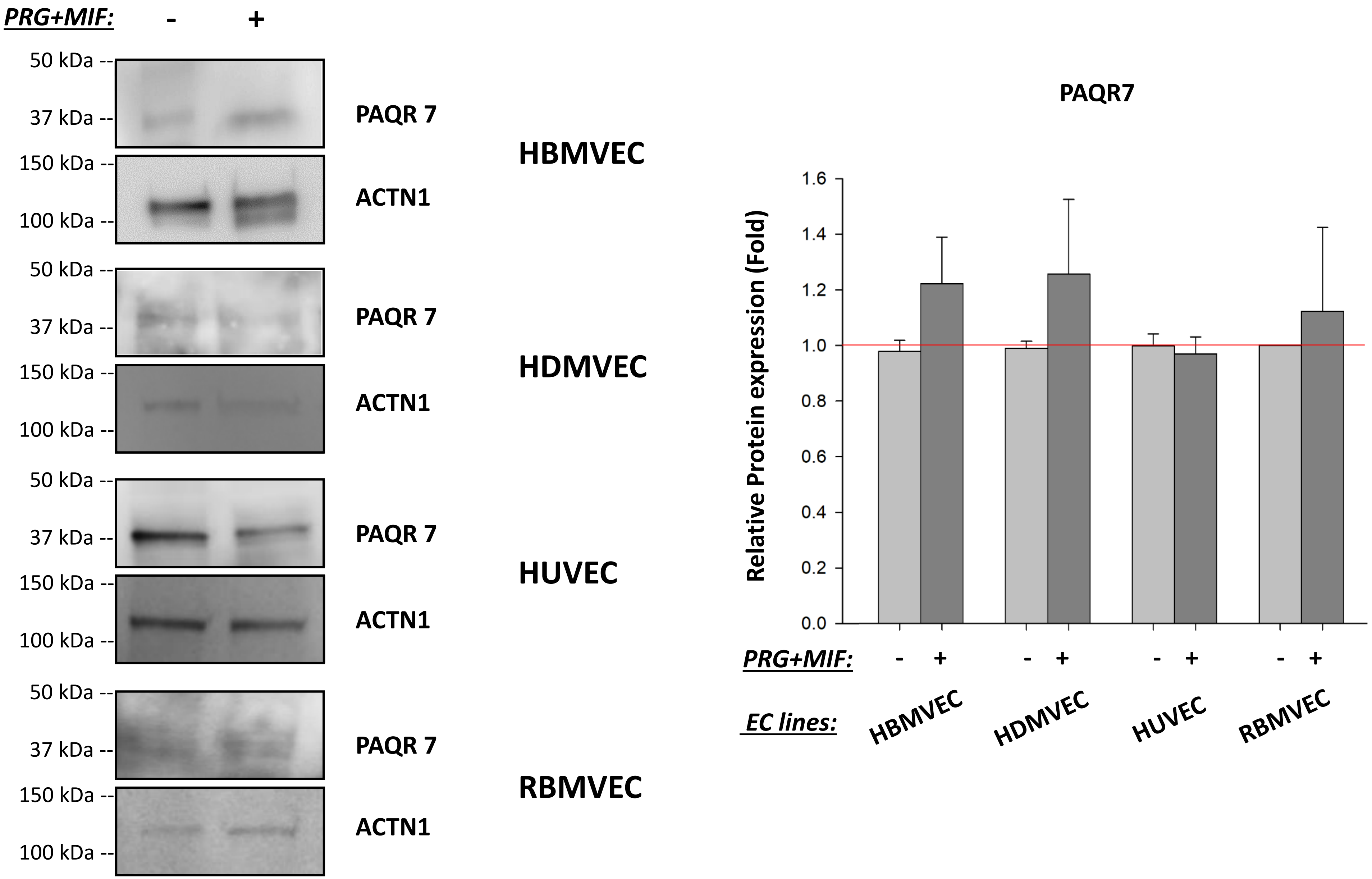
PRG+MIF: - +
50 kDa
37 kDa
PAQR 7
150 kDa
100 kDa
ACTN1
HBMVEC
50 kDa
37 kDa
PAQR 7
150 kDa
100 kDa
ACTN1
HDMVEC
50 kDa
37 kDa
PAQR 7
150 kDa
100 kDa
ACTN1
HUVEC
50 kDa
37 kDa
PAQR 7
150 kDa
100 kDa
ACTN1
RBMVEC
PAQR7
Relative Protein expression (Fold)
0.0
0.2
0.4
0.6
0.8
1.0
1.2
1.4
1.6
PRG+MIF: - + - + - + - +
EC lines: HBMVEC HDMVEC HUVEC RBMVEC

**Suppl. Fig. 3A**

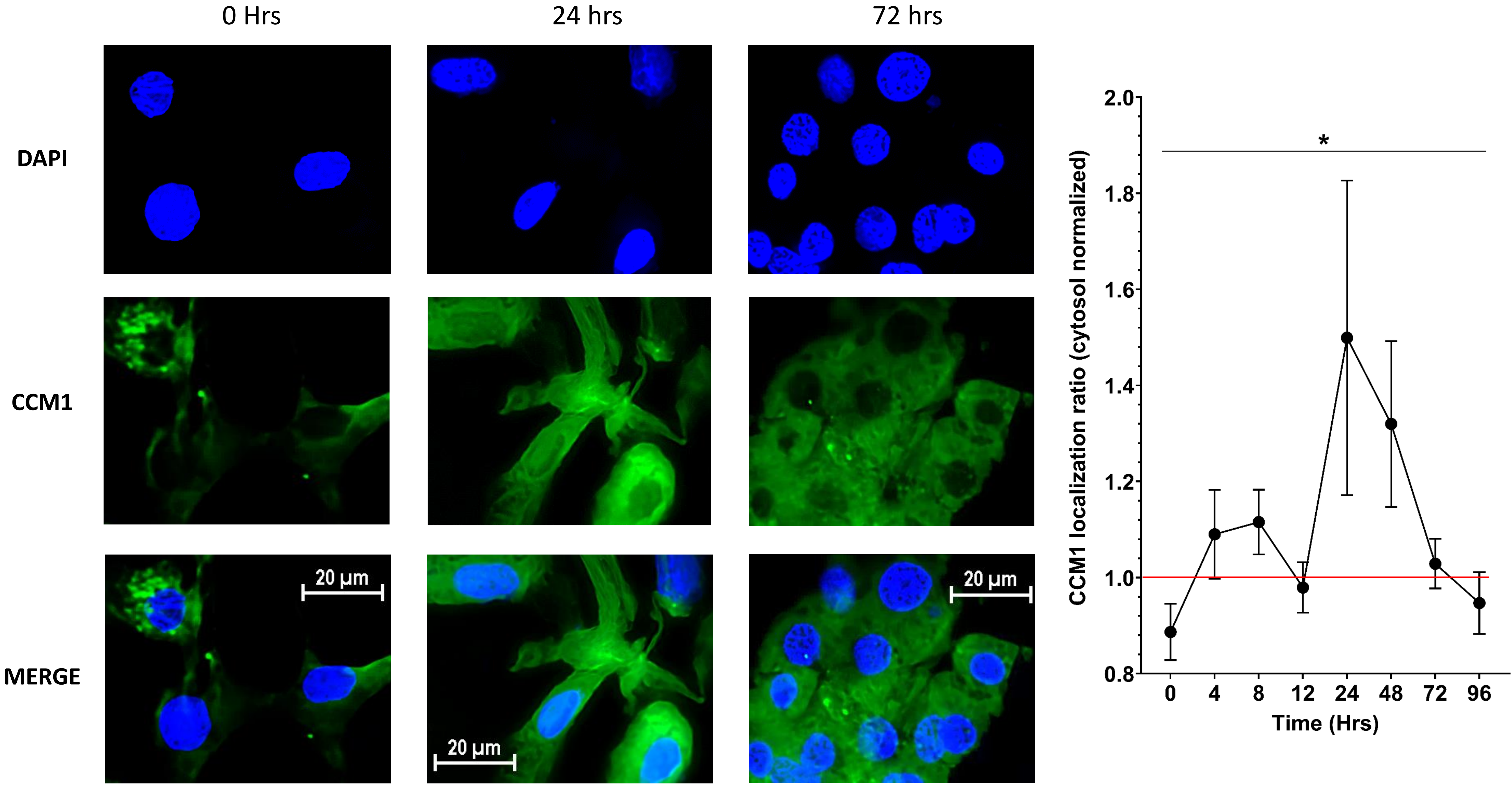
0 Hrs
24 hrs
72 hrs
DAPI
CCM1
MERGE
20 µm
20 µm
20 µm
2.0
1.8
1.6
1.4
1.2
1.0
0.8
CCM1 localization ratio (cytosol normalized)
*
0
4
8
12
24
48
72
96
Time (Hrs)

**Suppl. Fig. 3B**

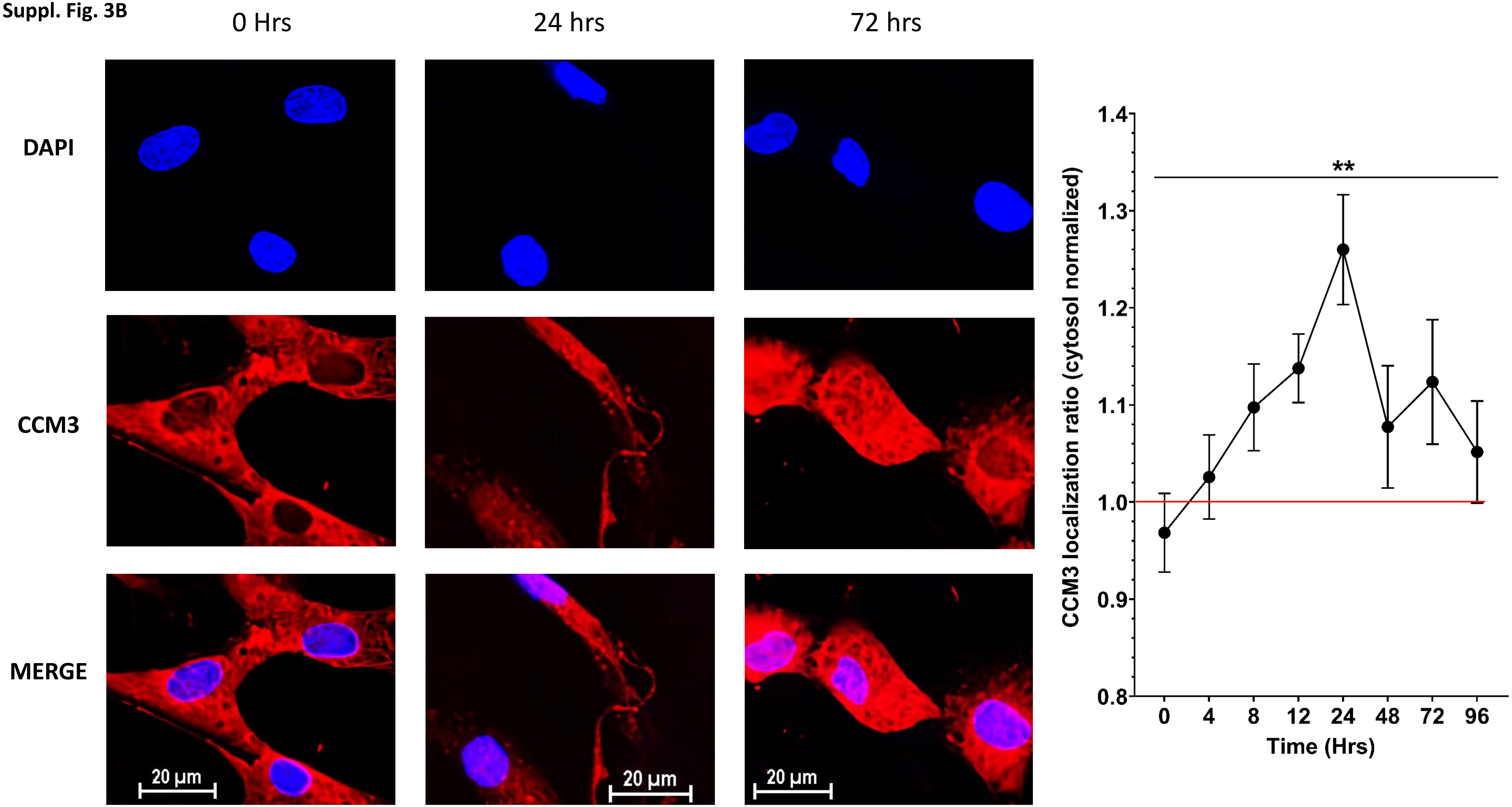

0 Hrs
24 hrs
72 hrs
DAPI
CCM3
MERGE
20 µm
20 µm
20 µm
CCM3 localization ratio (cytosol normalized)
1.4
1.3
1.2
1.1
1.0
0.9
0.8
**
0
4
8
12
24
48
72
96
Time (Hrs)

Suppl. Fig. 3C

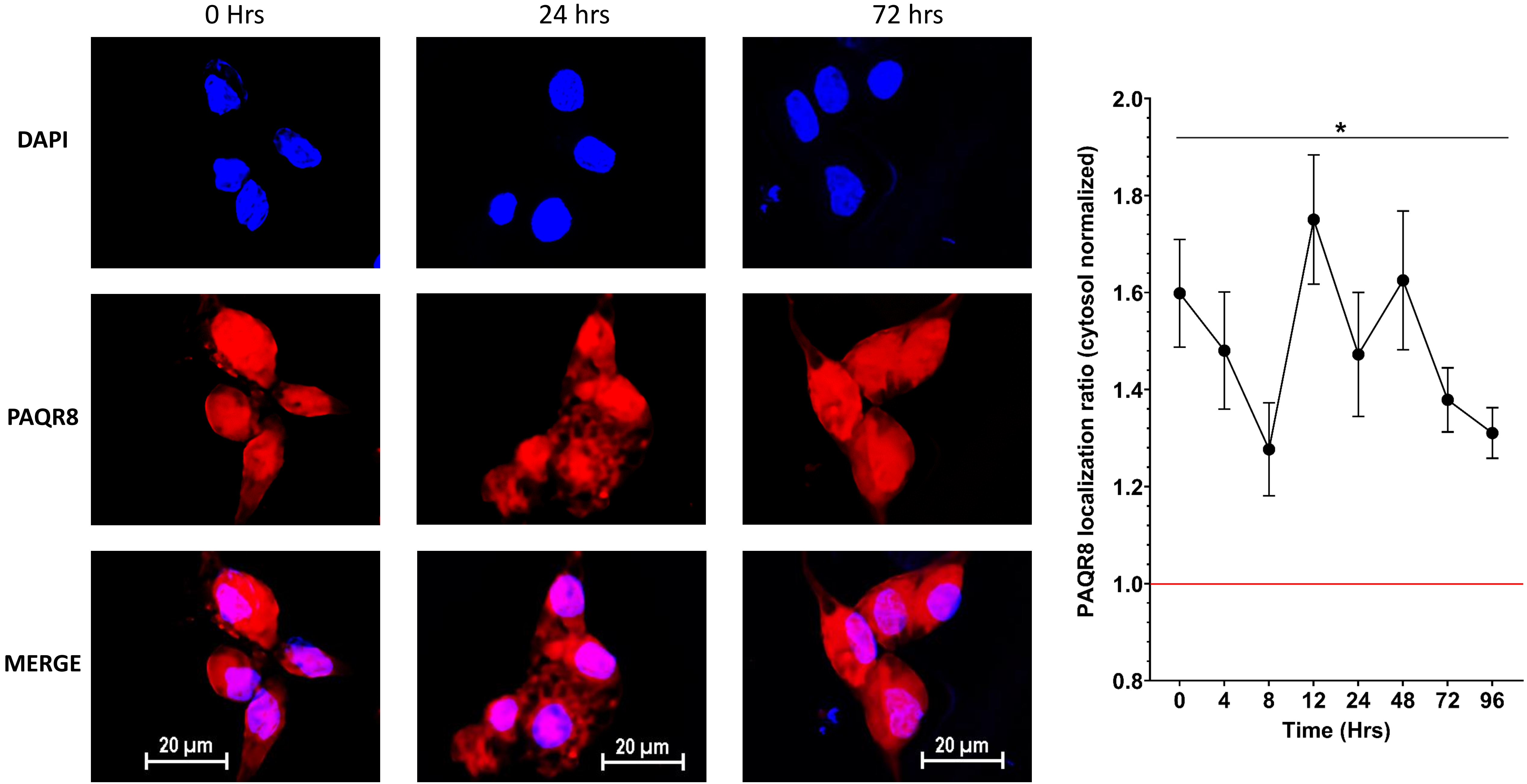

0 Hrs
24 hrs
72 hrs
DAPI
PAQR8
MERGE
20 µm
20 µm
20 µm
PAQR8 localization ratio (cytosol normalized)
2.0
1.8
1.6
1.4
1.2
1.0
0.8
*
0
4
8
12
24
48
72
96
Time (Hrs)

**Fig. S4A**

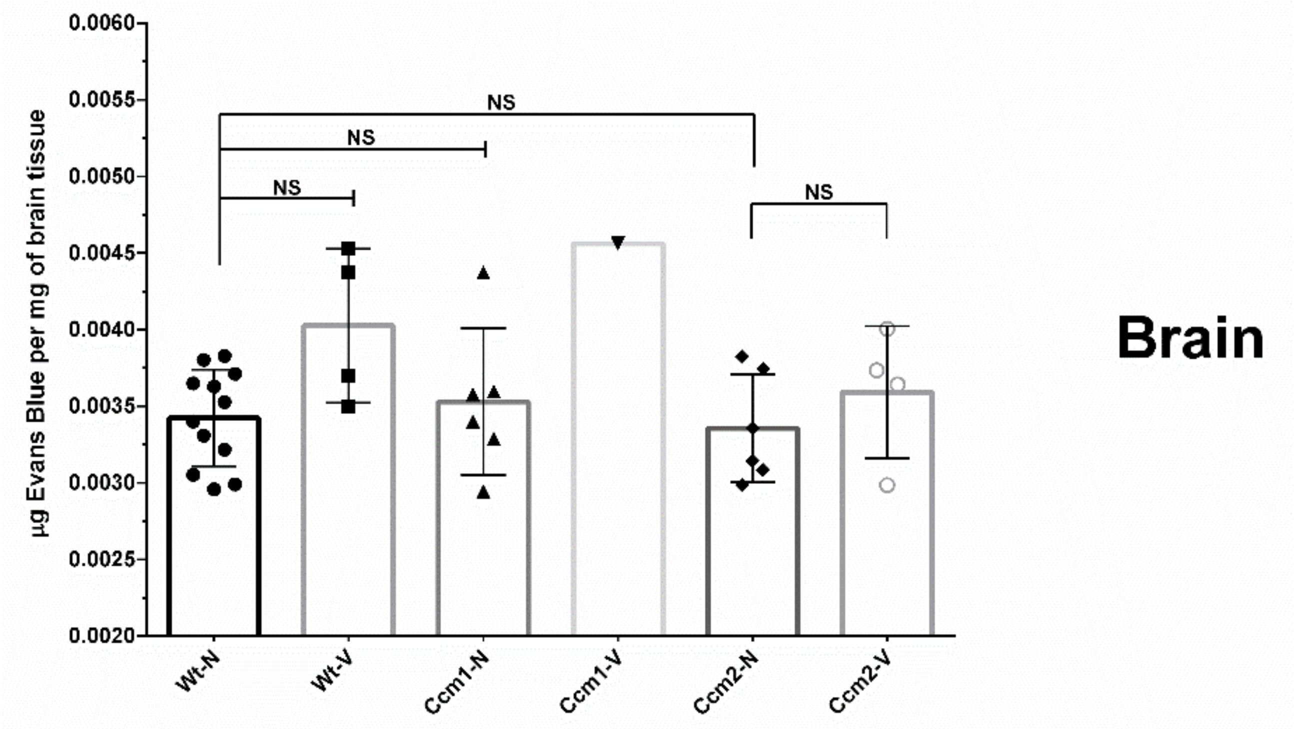

0.0060
0.0055
0.0050
0.0045
0.0040
0.0035
0.0030
0.0025
0.0020
µg Evans Blue per mg of brain tissue
NS
NS
NS
NS
Wt-N
Wt-V
Ccm1-N
Ccm1-V
Ccm2-N
Ccm2-V
Brain

**Fig. S4B**

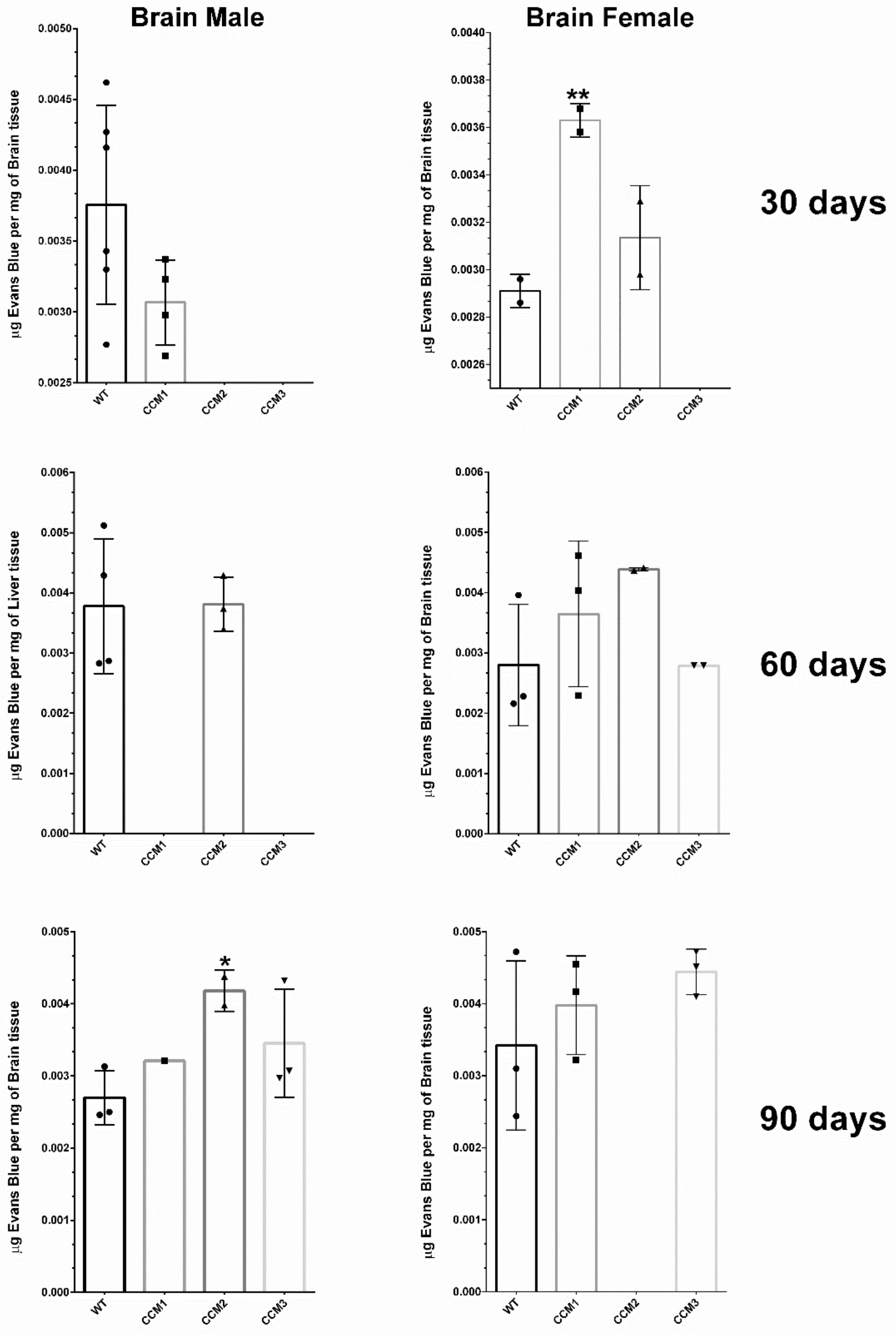
Brain Male
Brain Female
30 days
60 days
90 days
µg Evans Blue per mg of Brain tissue
µg Evans Blue per mg of Liver tissue
WT
CCM1
CCM2
CCM3
**
*
0.0050
0.0045
0.0040
0.0035
0.0030
0.0025
0.0040
0.0038
0.0036
0.0034
0.0032
0.0030
0.0028
0.0026
0.006
0.005
0.004
0.003
0.002
0.001
0.000

Fig. S4C

I. Control groups

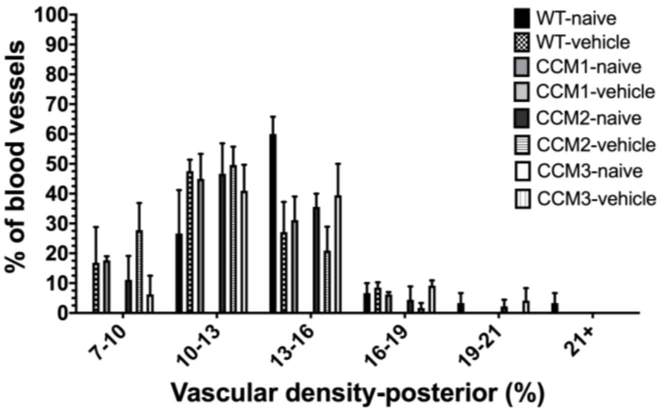


II. Treatment groups

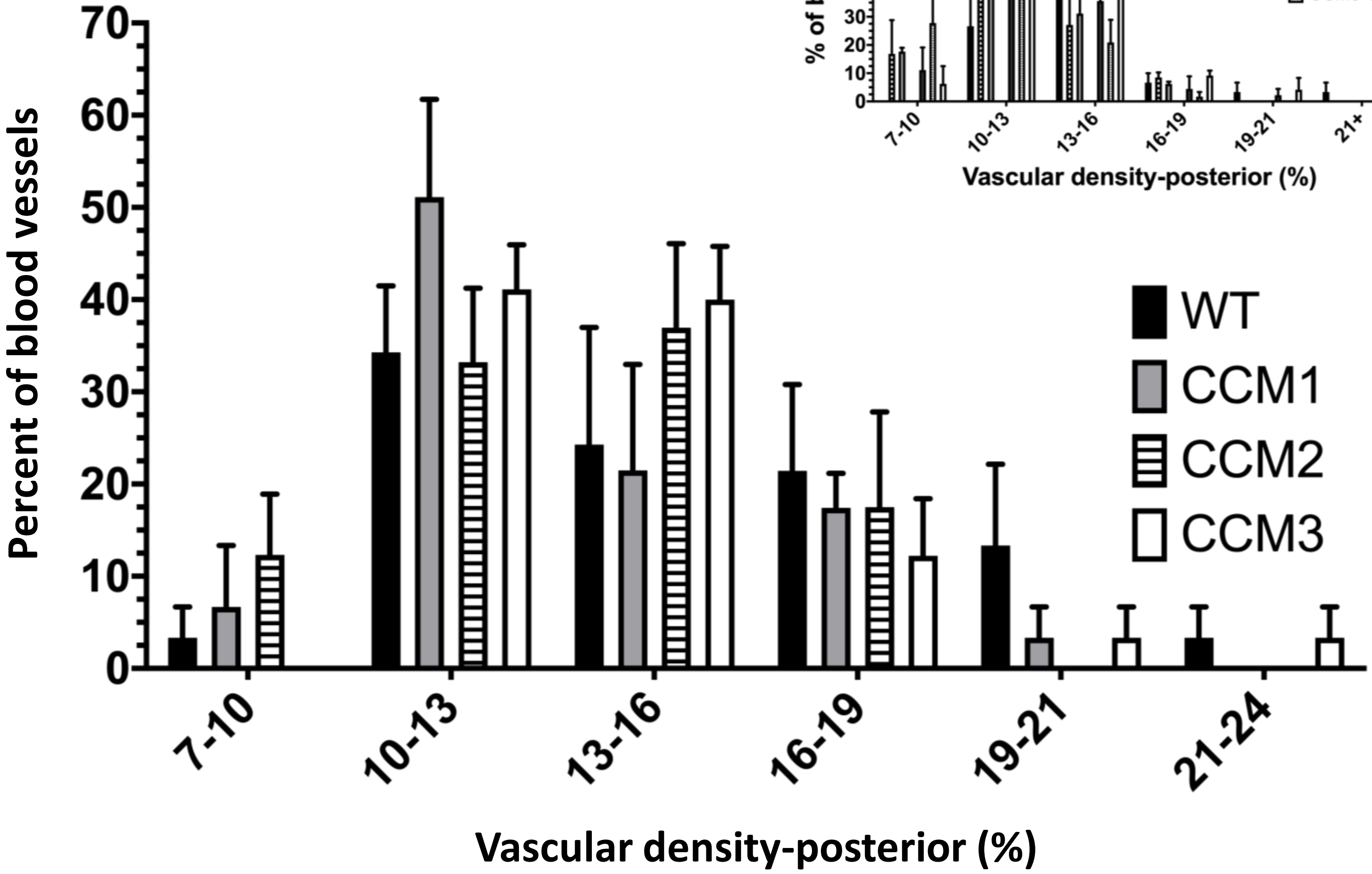

Fig. S4D

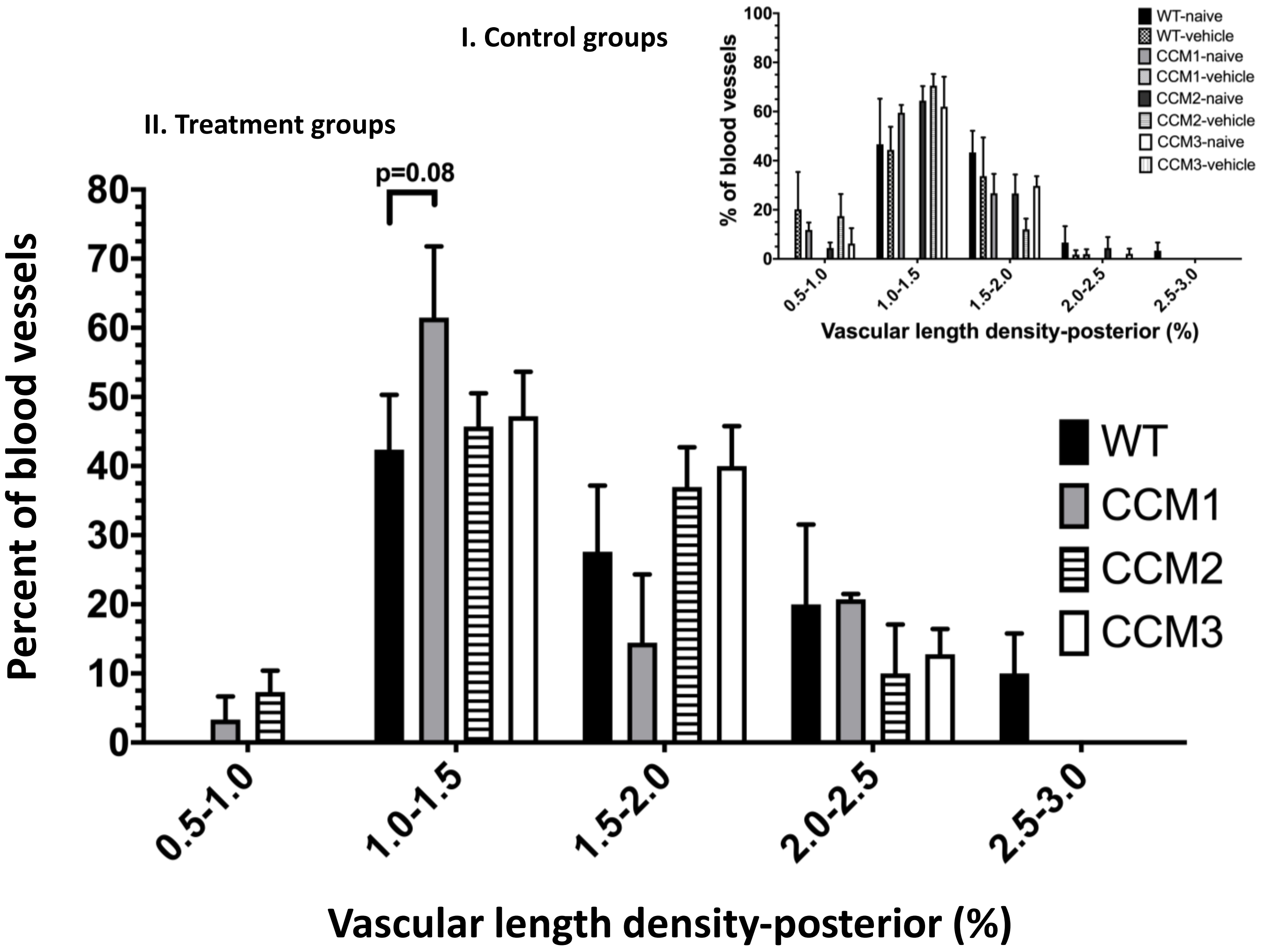
I. Control groups
100
80
60
40
20
0
% of blood vessels
0.5-1.0
1.0-1.5
1.5-2.0
2.0-2.5
2.5-3.0
Vascular length density-posterior (%)
WT-naive
WT-vehicle
CCM1-naive
CCM1-vehicle
CCM2-naive
CCM2-vehicle
CCM3-naive
CCM3-vehicle
II. Treatment groups
p=0.08
80
70
60
50
40
30
20
10
0
Percent of blood vessels
0.5-1.0
1.0-1.5
1.5-2.0
2.0-2.5
2.5-3.0
Vascular length density-posterior (%)
WT
CCM1
CCM2
CCM3

Fig. S4E

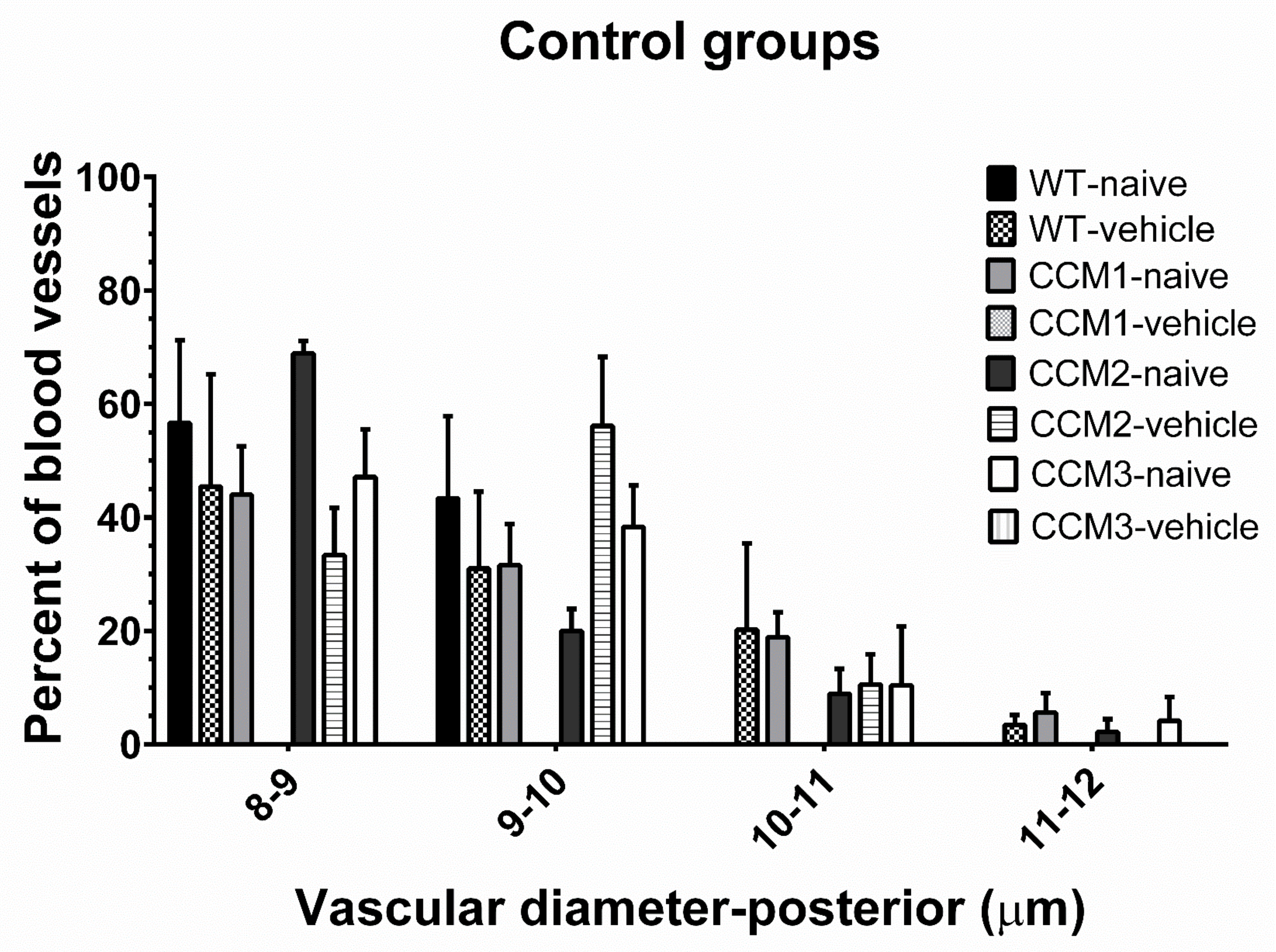

Control groups
Percent of blood vessels
100
80
60
40
20
0
8-9
9-10
10-11
11-12
Vascular diameter-posterior (μm)
WT-naive
WT-vehicle
CCM1-naive
CCM1-vehicle
CCM2-naive
CCM2-vehicle
CCM3-naive
CCM3-vehicle

Fig. S4F

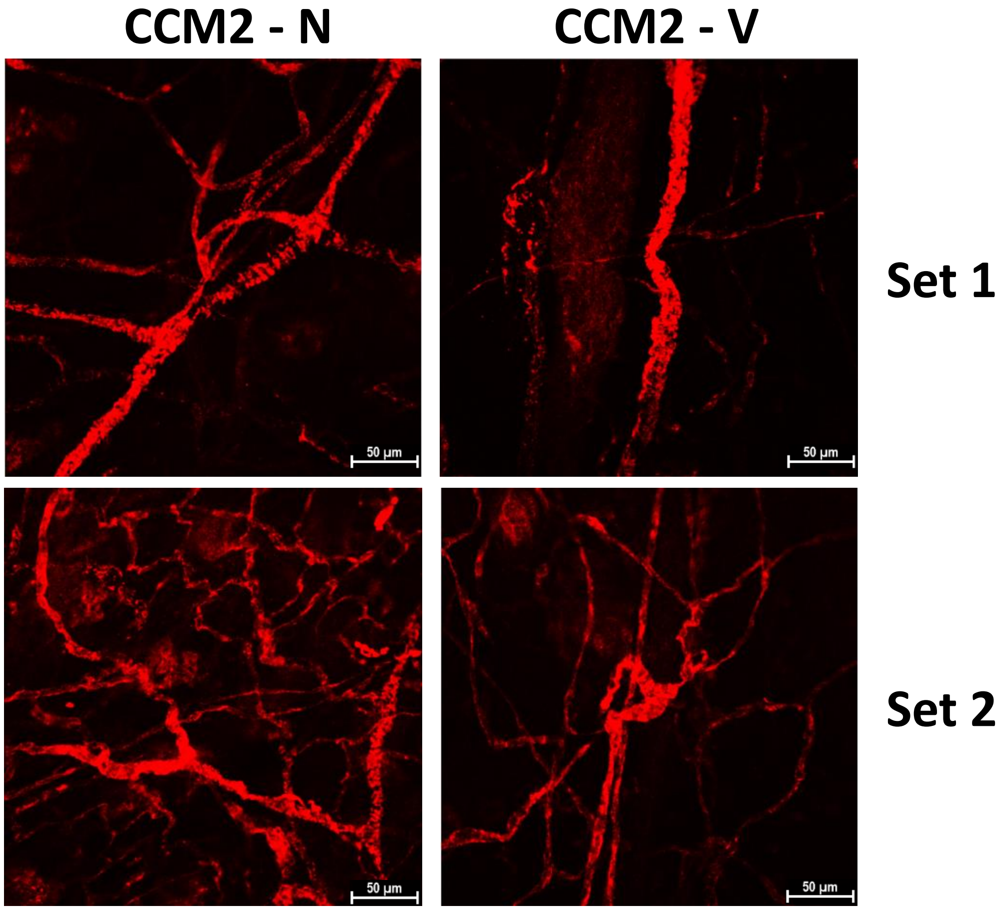
CCM2 - N
CCM2 - V
Set 1
Set 2
50 µm
50 µm
50 µm
50 µm

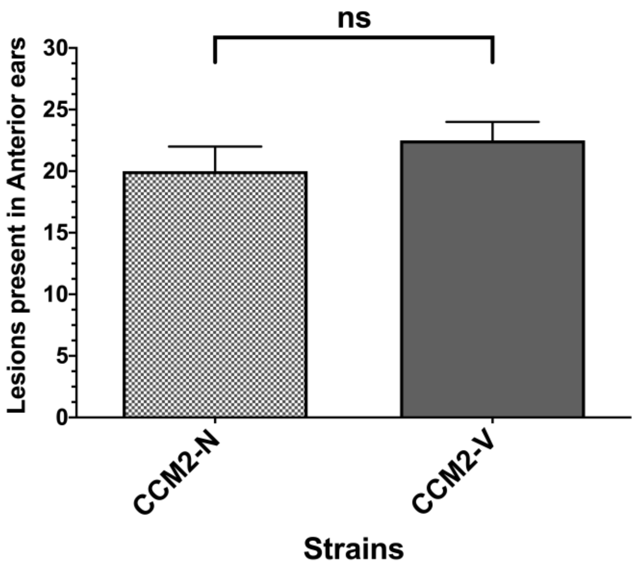
ns
Lesions present in Anterior ears
30
25
20
15
10
5
0
CCM2-N
CCM2-V
Strains

Fig. S5

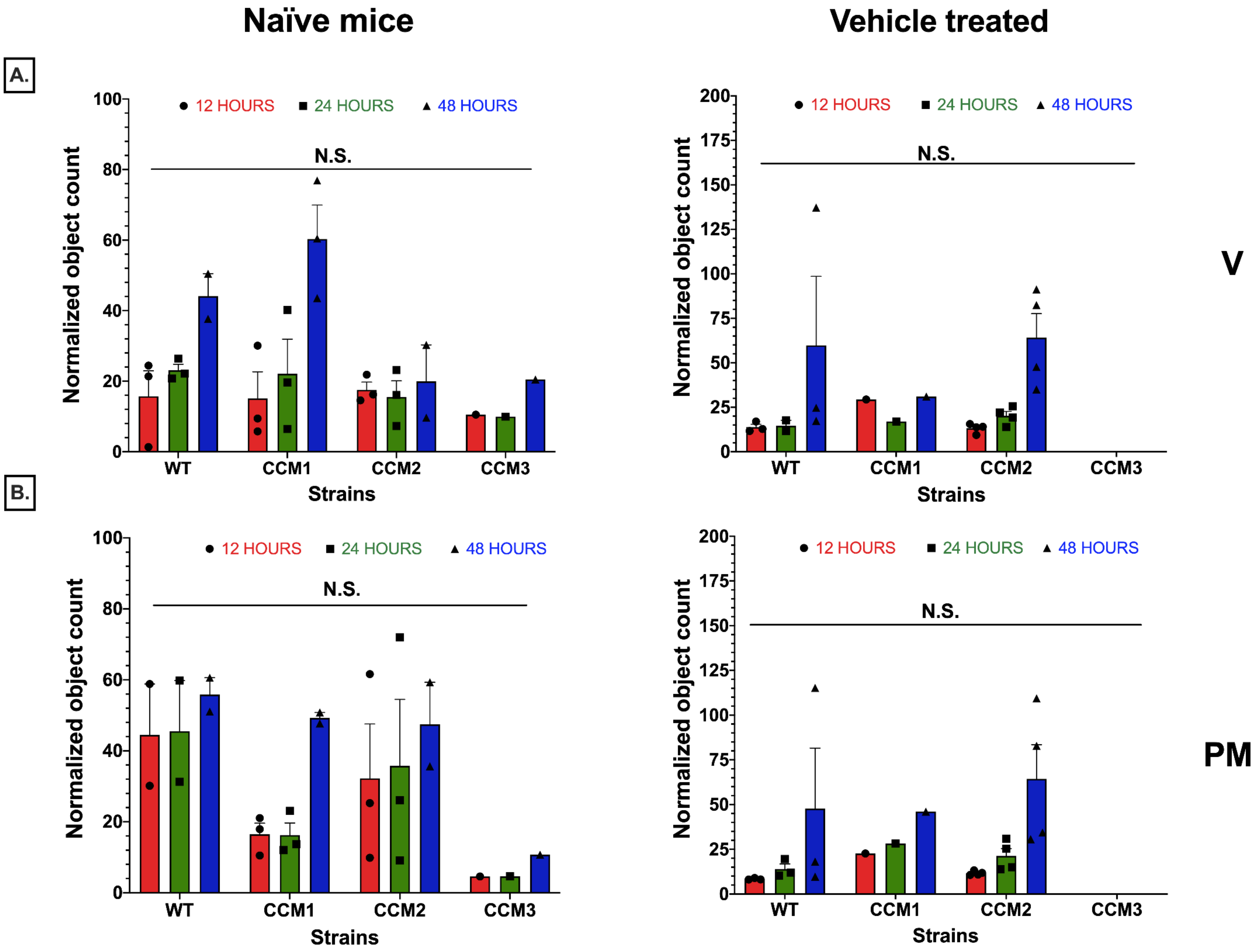
Naïve mice
Vehicle treated
A.
B.
12 HOURS
24 HOURS
48 HOURS
N.S.
Normalized object count
Strains
WT
CCM1
CCM2
CCM3
V
PM

Fig. S5

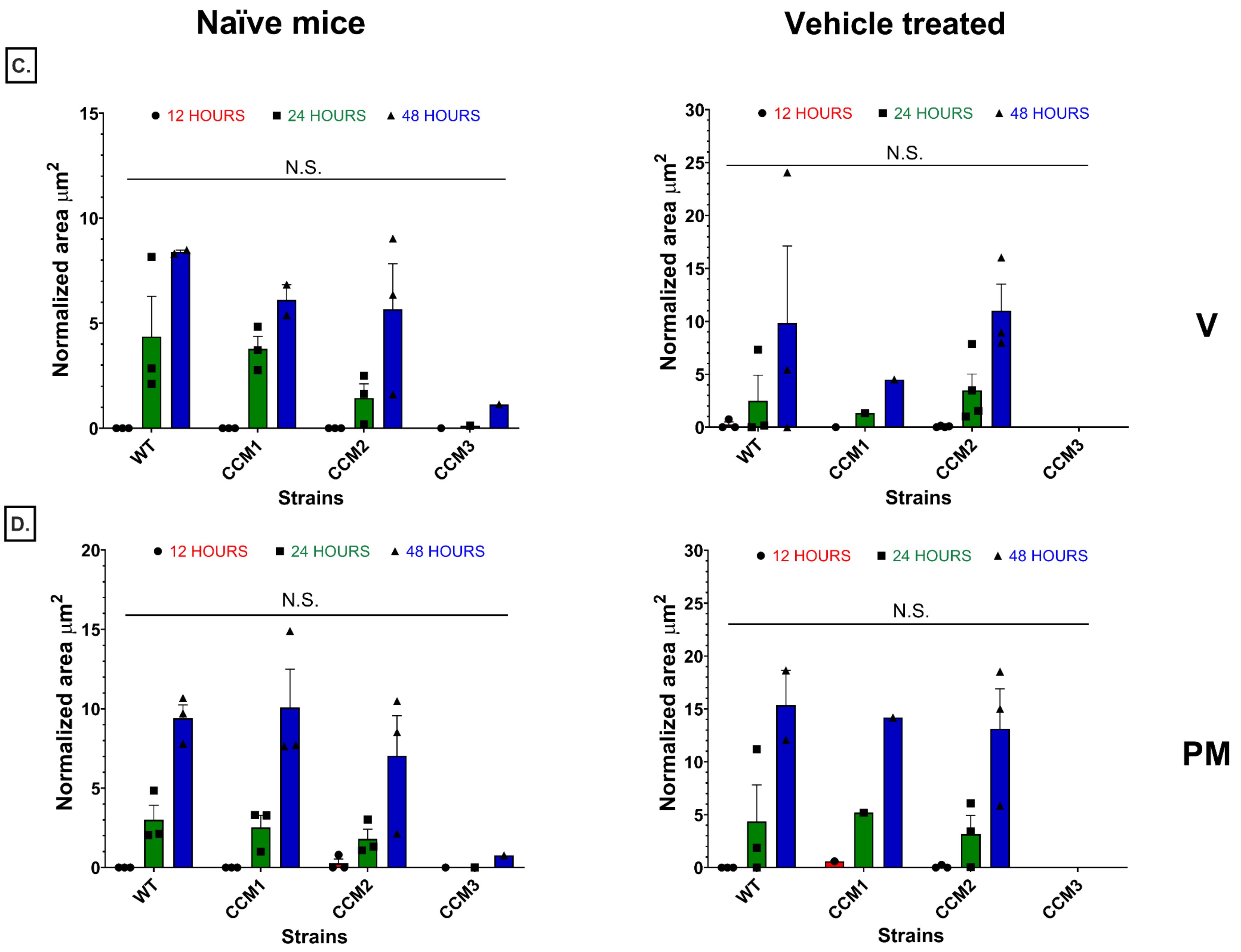

Naïve mice
Vehicle treated
C.
D.
12 HOURS
24 HOURS
48 HOURS
N.S.
Normalized area μm²
WT
CCM1
CCM2
CCM3
Strains
V
PM

Fig. S5

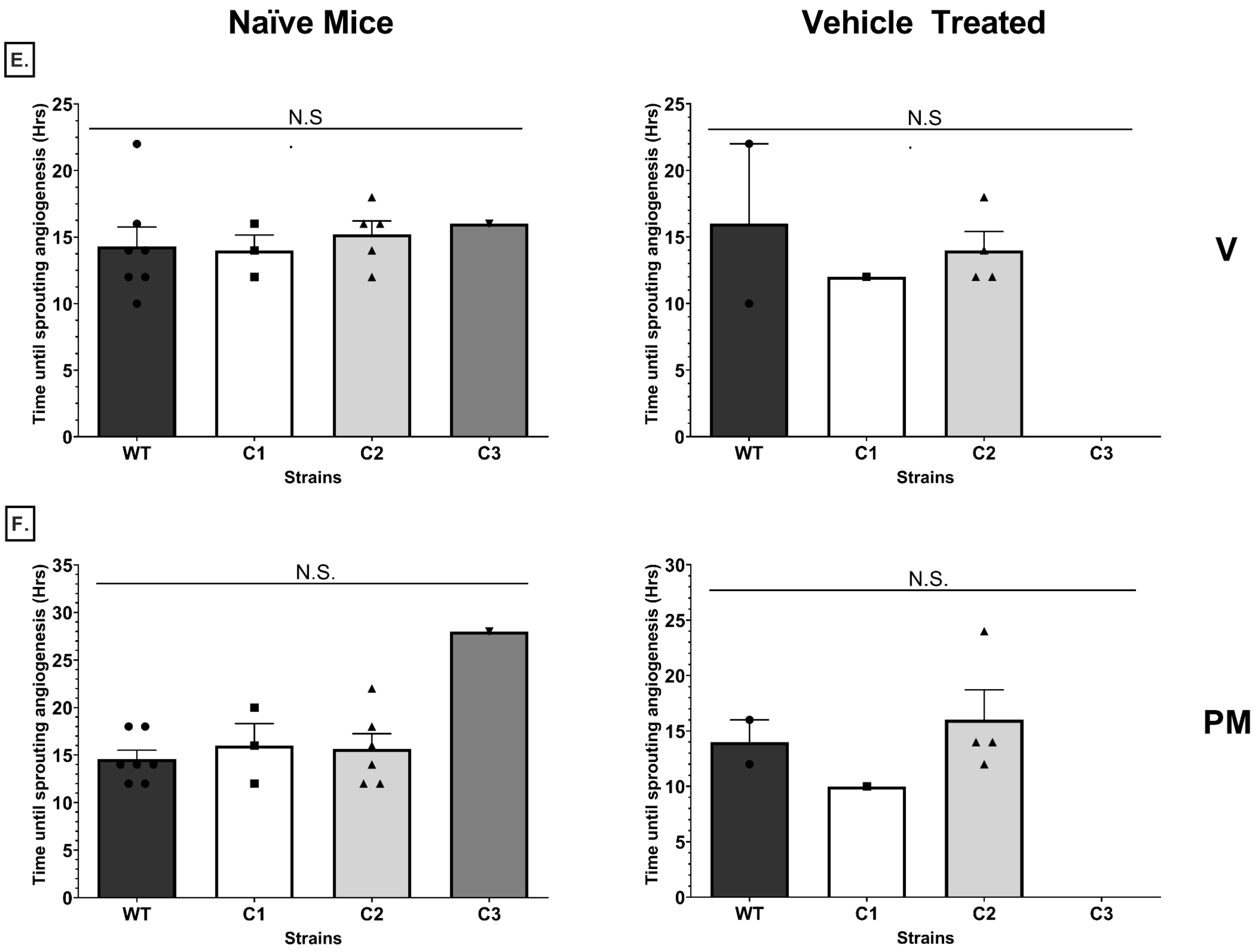
Naïve Mice
Vehicle Treated
E.
F.
N.S
N.S.
Time until sprouting angiogenesis (Hrs)
WT
C1
C2
C3
Strains
V
PM

**Fig. S5**

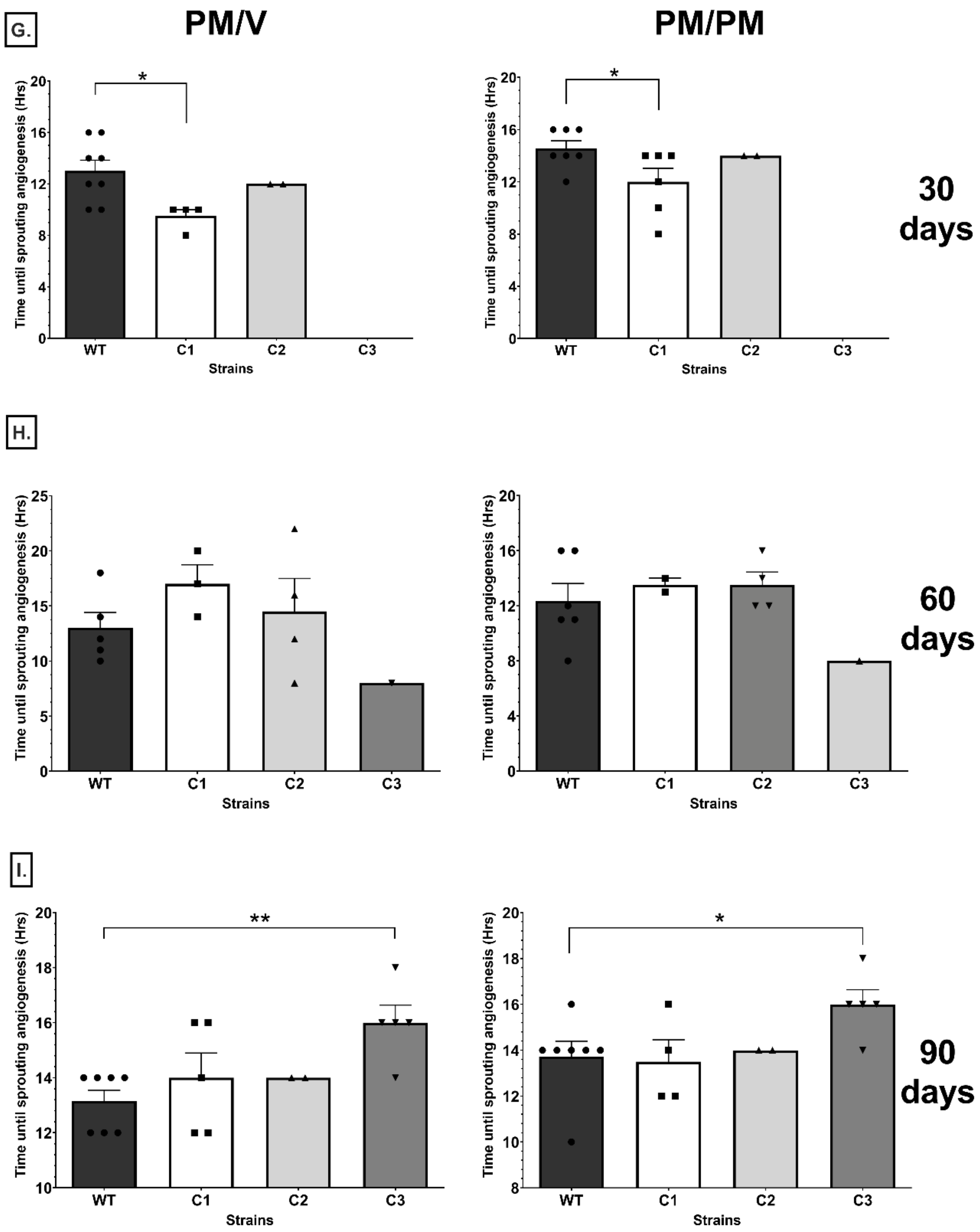
G.
PM/V
PM/PM
H.
I.
30 days
60 days
90 days
Time until sprouting angiogenesis (Hrs)
Strains
WT
C1
C2
C3
*
**

## Fig. S6A-I

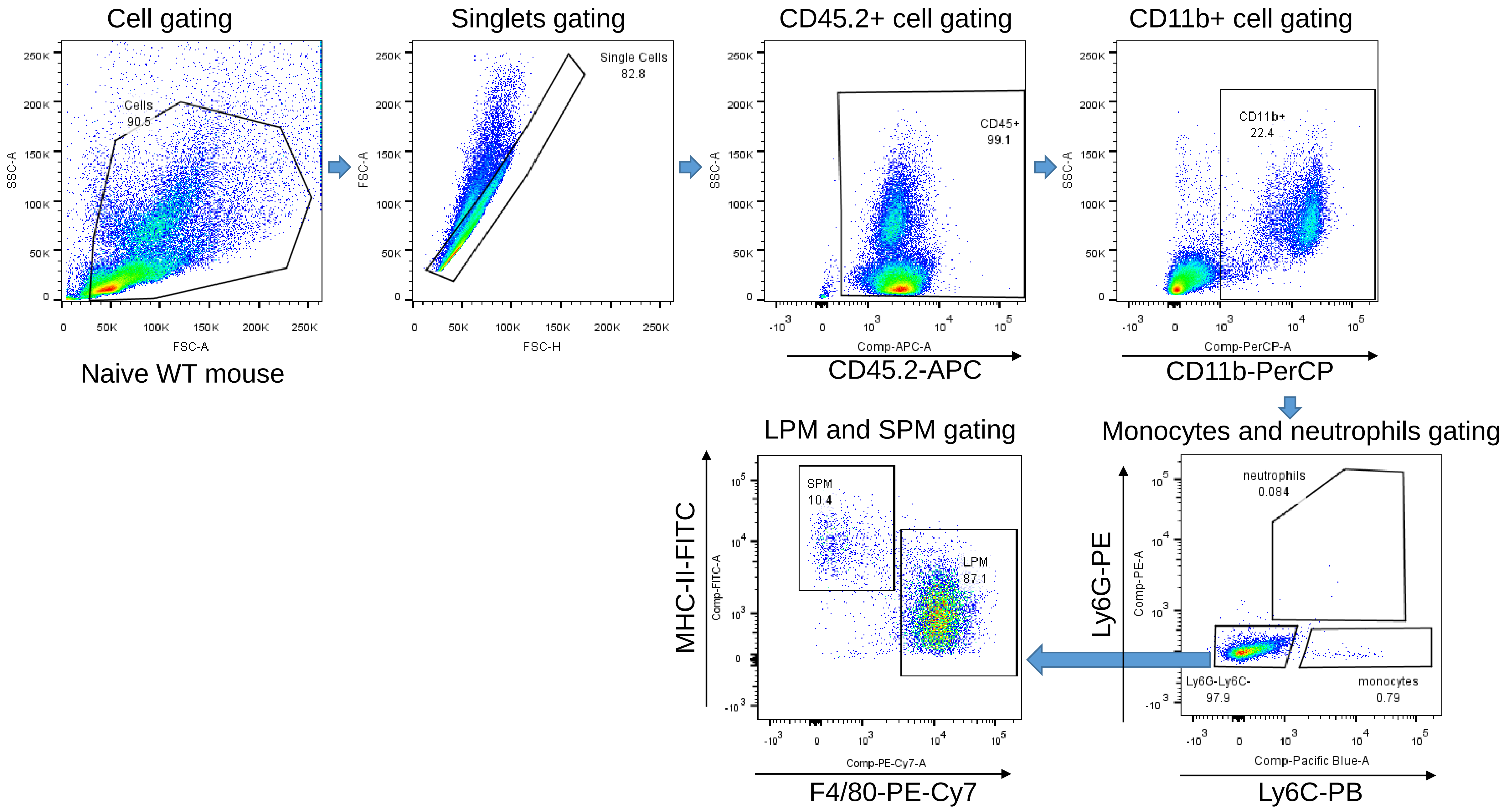

Cell gating
Singlets gating
CD45.2+ cell gating
CD11b+ cell gating
Cells 90.5
Single Cells 82.8
CD45+ 99.1
CD11b+ 22.4
SSC-A
FSC-A
FSC-H
Comp-APC-A
Comp-PerCP-A
Naive WT mouse
CD45.2-APC
CD11b-PerCP
LPM and SPM gating
Monocytes and neutrophils gating
SPM 10.4
LPM 87.1
neutrophils 0.084
Ly6G-Ly6C- 97.9
monocytes 0.79
MHC-II-FITC
Comp-FITC-A
Comp-PE-Cy7-A
F4/80-PE-Cy7
Ly6G-PE
Comp-PE-A
Comp-Pacific Blue-A
Ly6C-PB

**Fig. S6A-II**

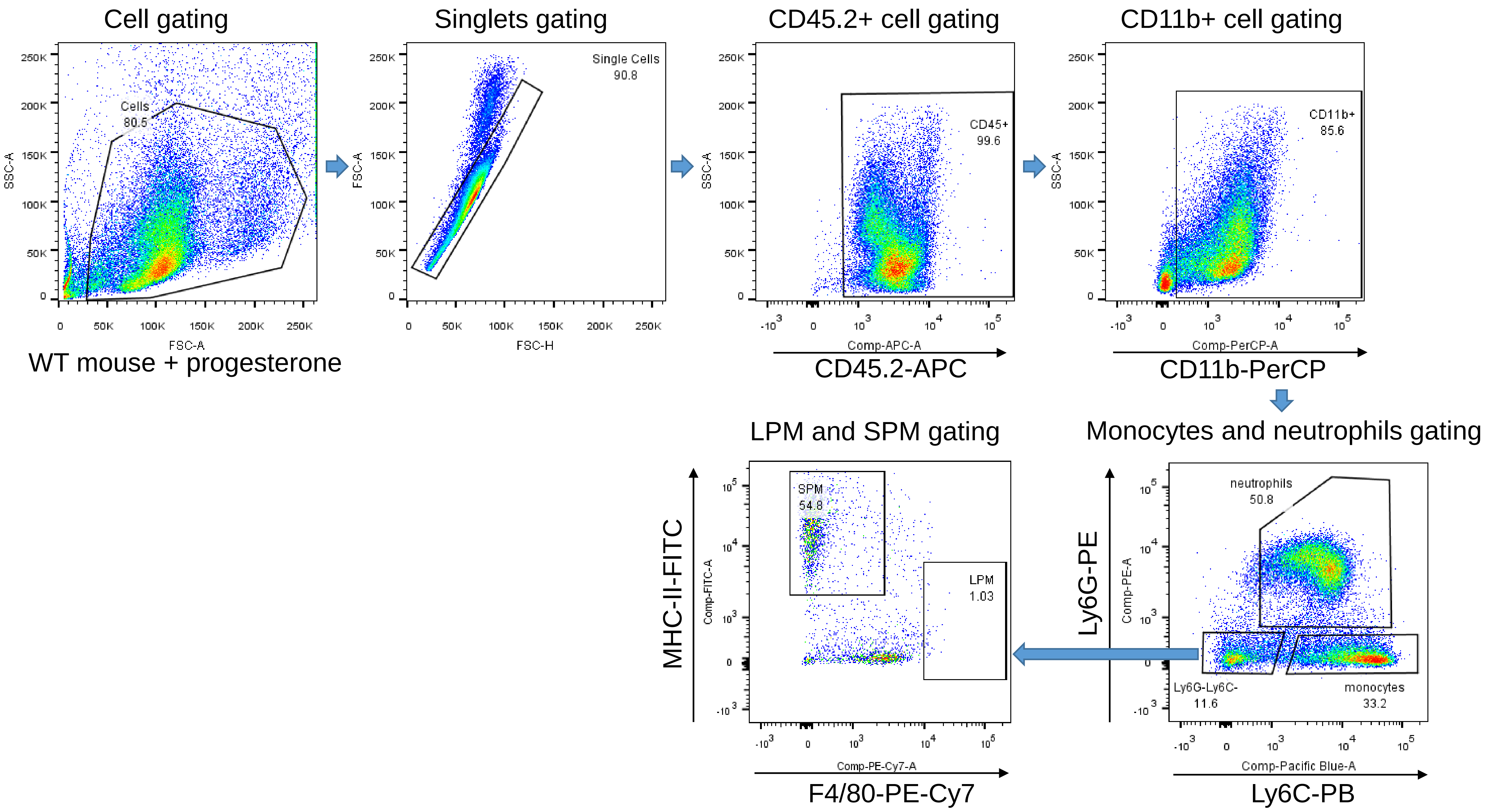
Cell gating
Singlets gating
CD45.2+ cell gating
CD11b+ cell gating
Cells
80.5
Single Cells
90.8
CD45+
99.6
CD11b+
85.6
SSC-A
FSC-A
FSC-H
Comp-APC-A
Comp-PerCP-A
WT mouse + progesterone
CD45.2-APC
CD11b-PerCP
LPM and SPM gating
Monocytes and neutrophils gating
SPM
54.8
LPM
1.03
neutrophils
50.8
Ly6G-Ly6C-
11.6
monocytes
33.2
Comp-FITC-A
Comp-PE-A
Comp-PE-Cy7-A
Comp-Pacific Blue-A
MHC-II-FITC
Ly6G-PE
F4/80-PE-Cy7
Ly6C-PB

Fig. S6B

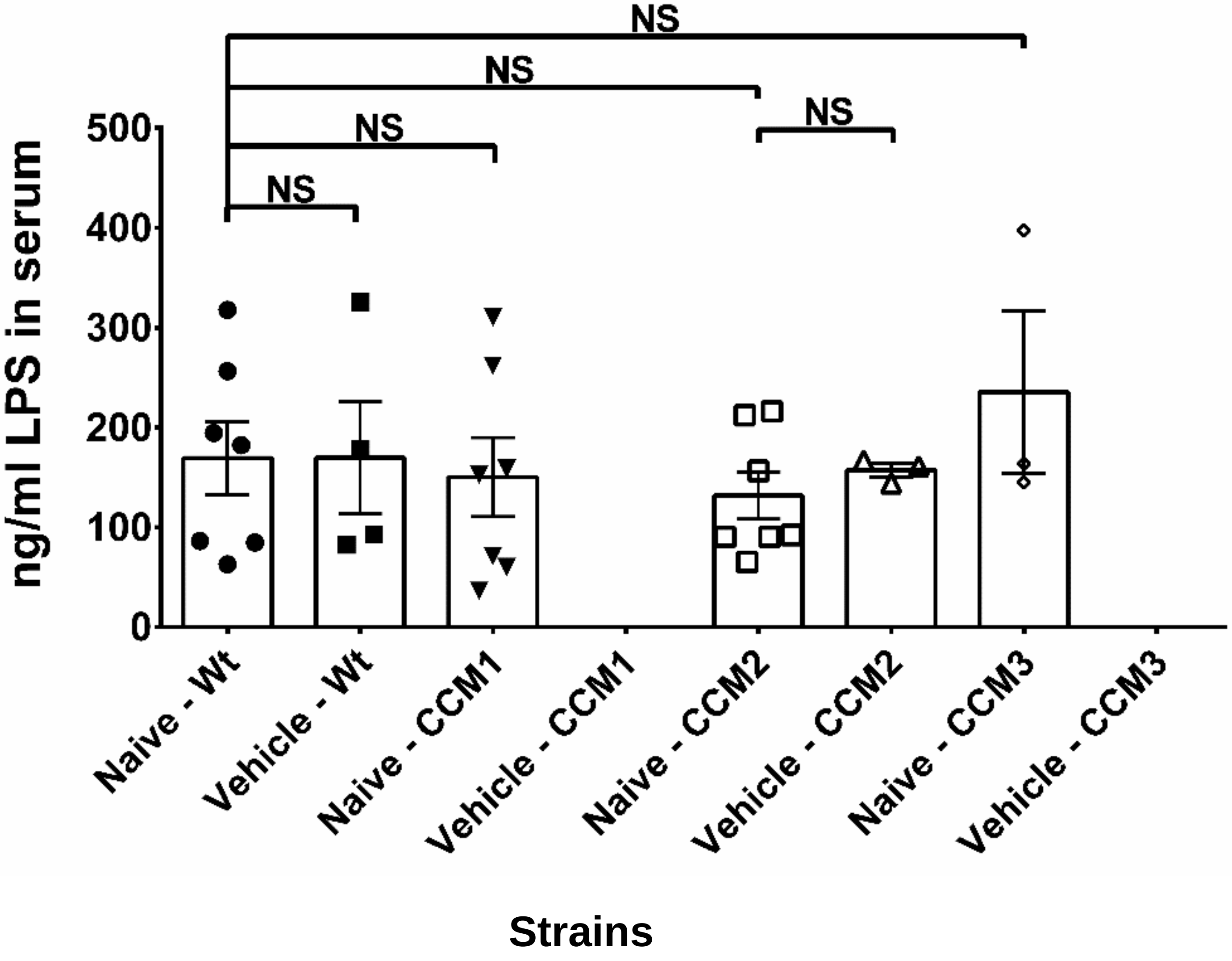

NS
NS
NS
NS
NS
500
400
300
200
100
0
ng/ml LPS in serum
Naive - Wt
Vehicle - Wt
Naive - CCM1
Vehicle - CCM1
Naive - CCM2
Vehicle - CCM2
Naive - CCM3
Vehicle - CCM3
Strains

**Fig. S6C**

**I. Naïve and Vehicle treatment**

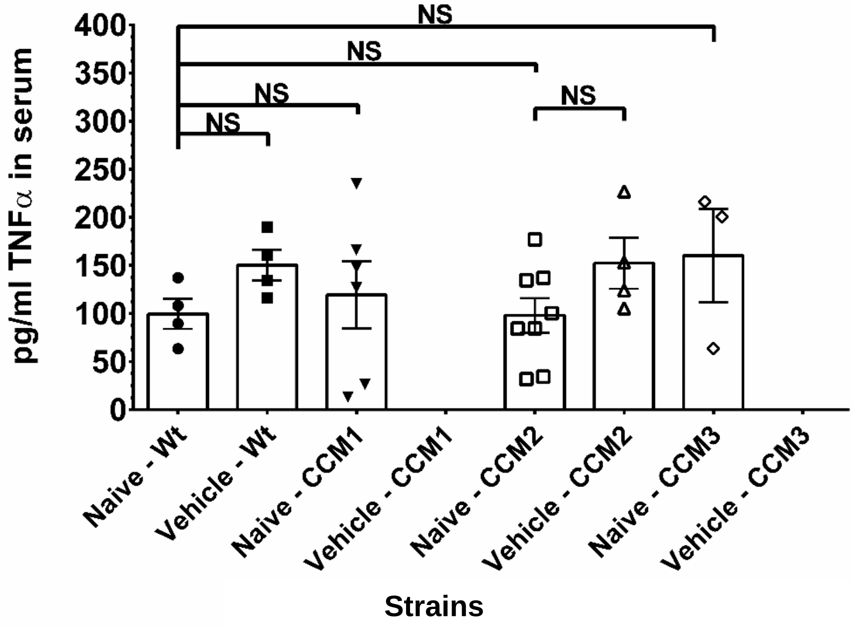


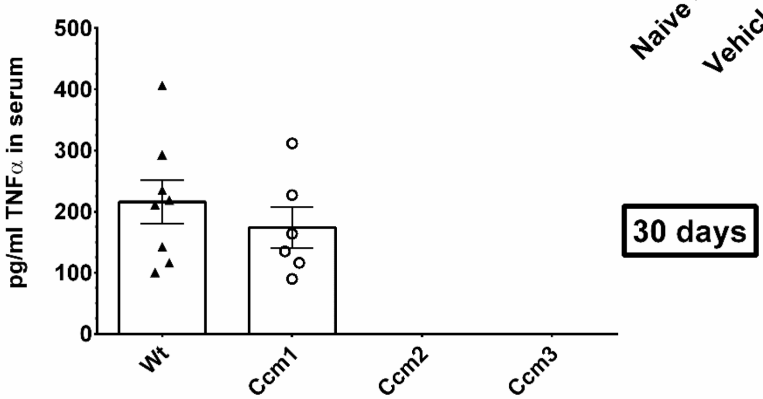


**II. combined steroid (PRG+MIF) hormone treatments**

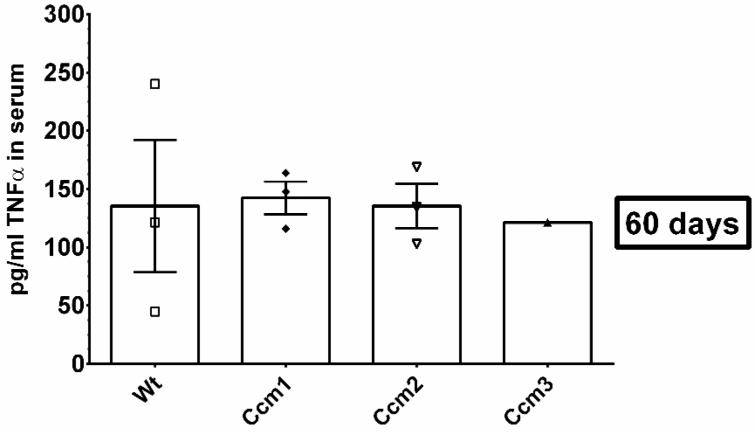


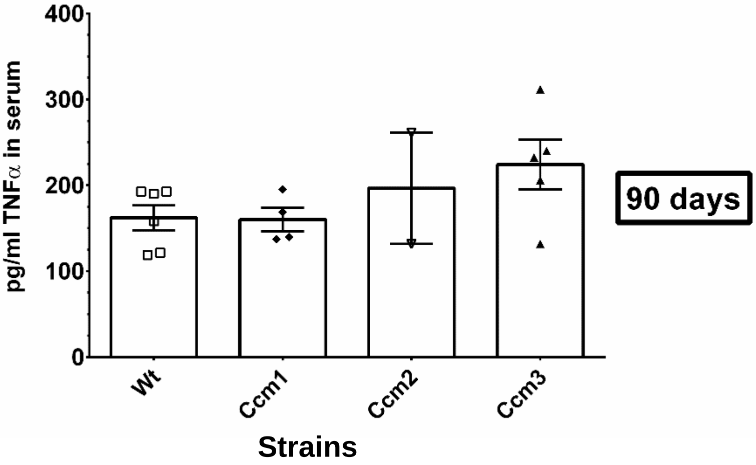

**Fig. S6D**

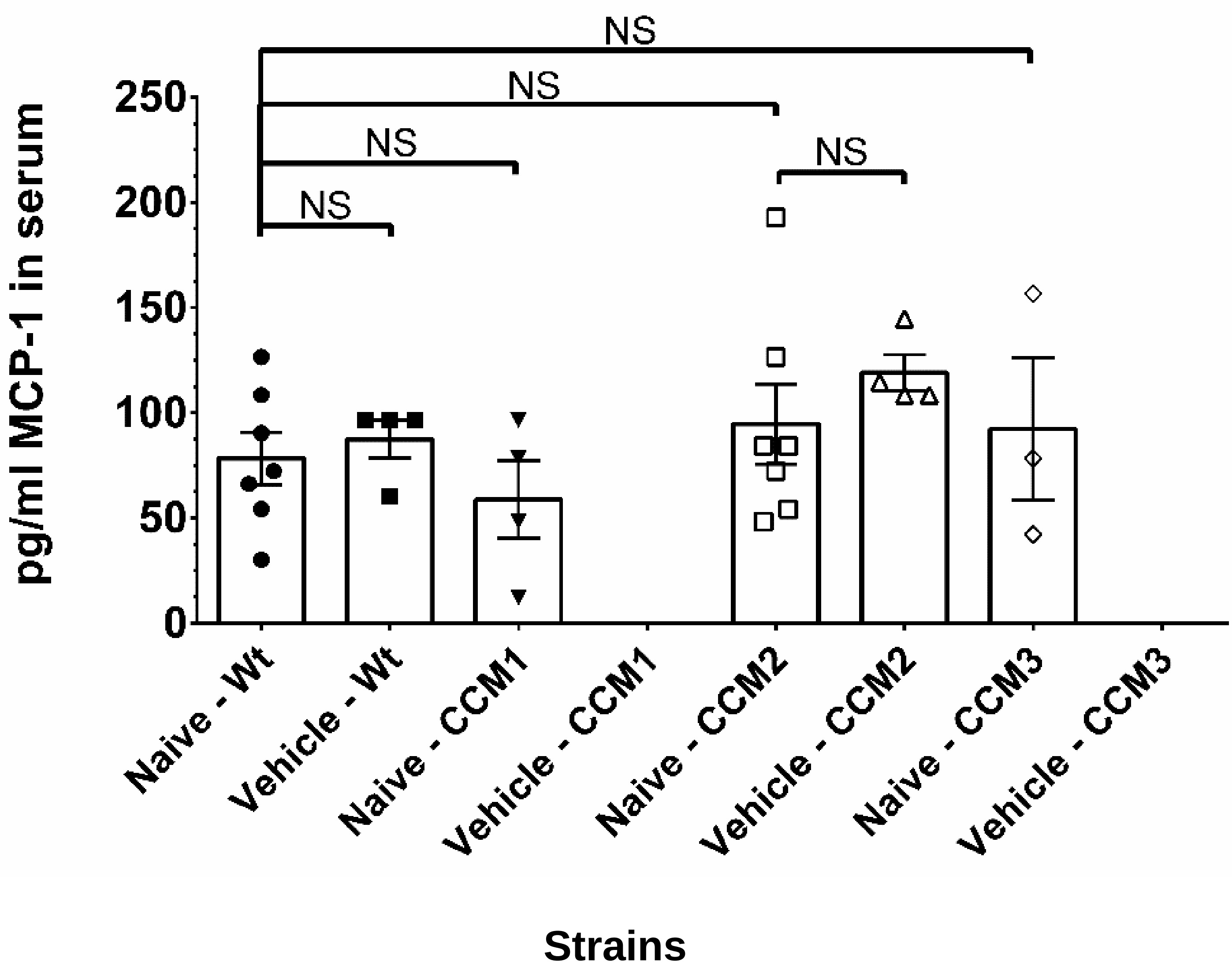
NS
NS
NS
NS
NS
pg/ml MCP-1 in serum
250
200
150
100
50
0
Naive - Wt
Vehicle - Wt
Naive - CCM1
Vehicle - CCM1
Naive - CCM2
Vehicle - CCM2
Naive - CCM3
Vehicle - CCM3
Strains

Fig. S6E

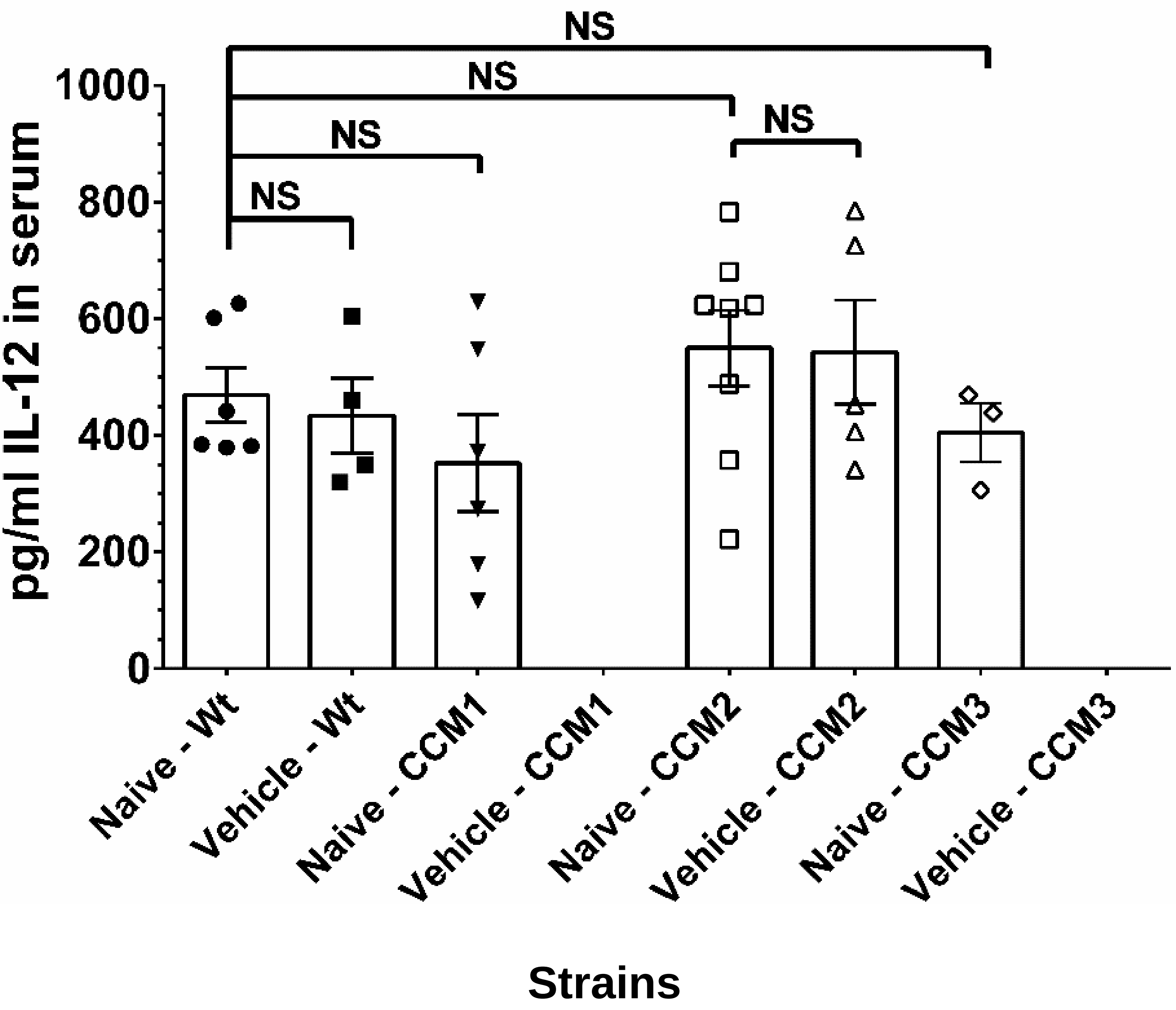

NS
NS
NS
NS
NS
pg/ml IL-12 in serum
1000
800
600
400
200
0
Naive - Wt
Vehicle - Wt
Naive - CCM1
Vehicle - CCM1
Naive - CCM2
Vehicle - CCM2
Naive - CCM3
Vehicle - CCM3
Strains

Fig. S6F

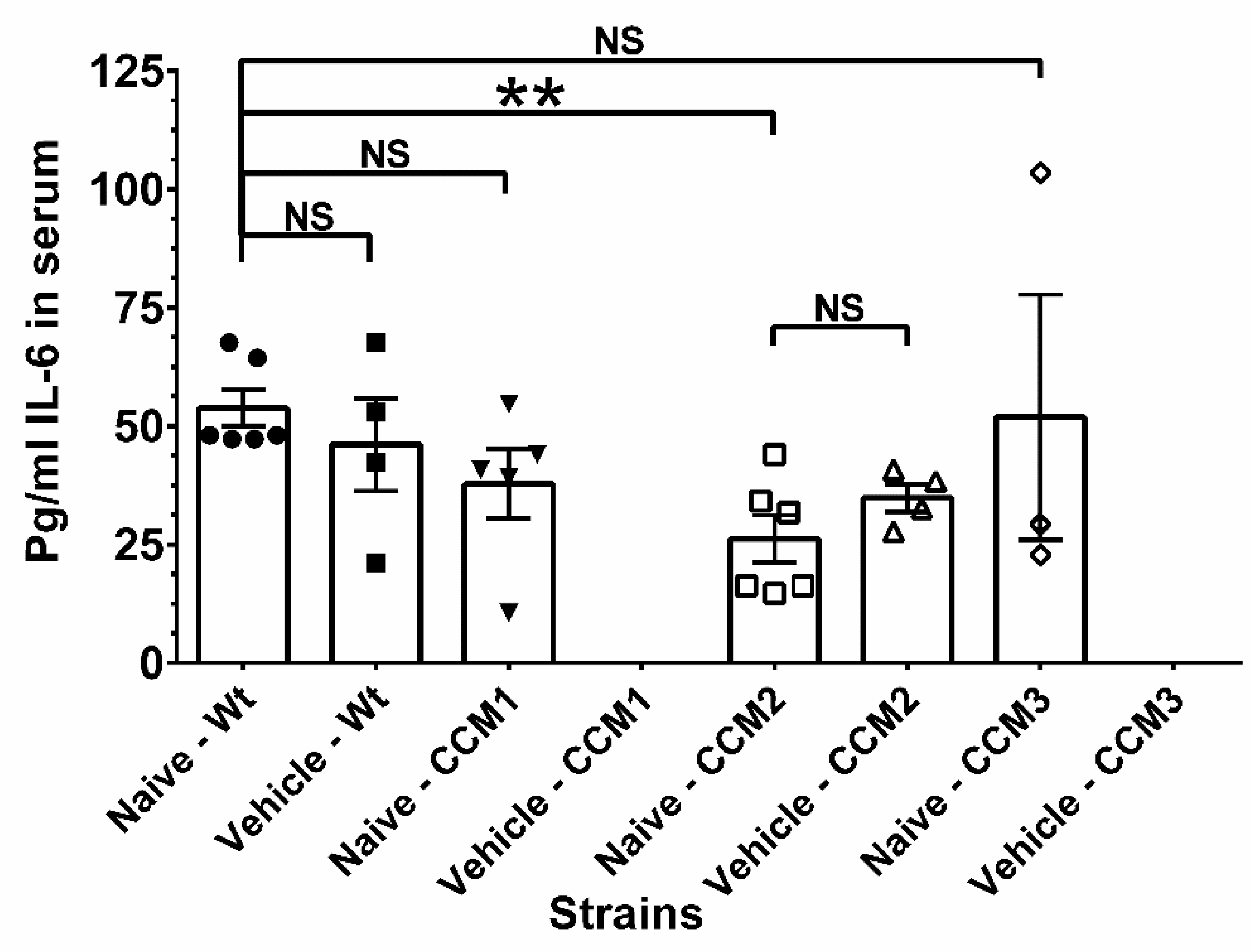
NS
**
NS
NS
NS
125
100
75
50
25
0
Pg/ml IL-6 in serum
Naive - Wt
Vehicle - Wt
Naive - CCM1
Vehicle - CCM1
Naive - CCM2
Vehicle - CCM2
Naive - CCM3
Vehicle - CCM3
Strains

Fig. S7A

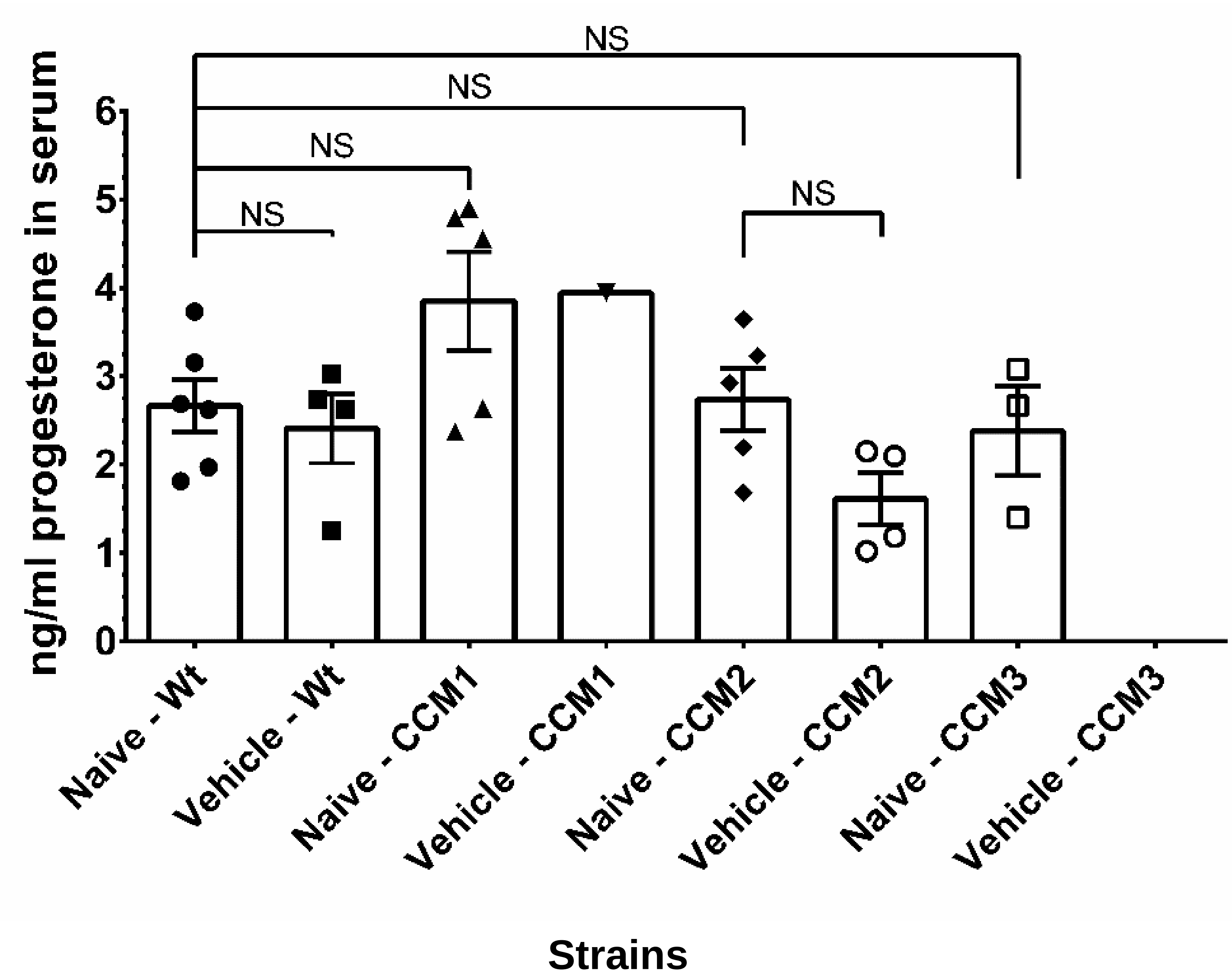
NS
NS
NS
NS
NS
ng/ml progesterone in serum
0
1
2
3
4
5
6
Naive - Wt
Vehicle - Wt
Naive - CCM1
Vehicle - CCM1
Naive - CCM2
Vehicle - CCM2
Naive - CCM3
Vehicle - CCM3
Strains

Fig. S7B

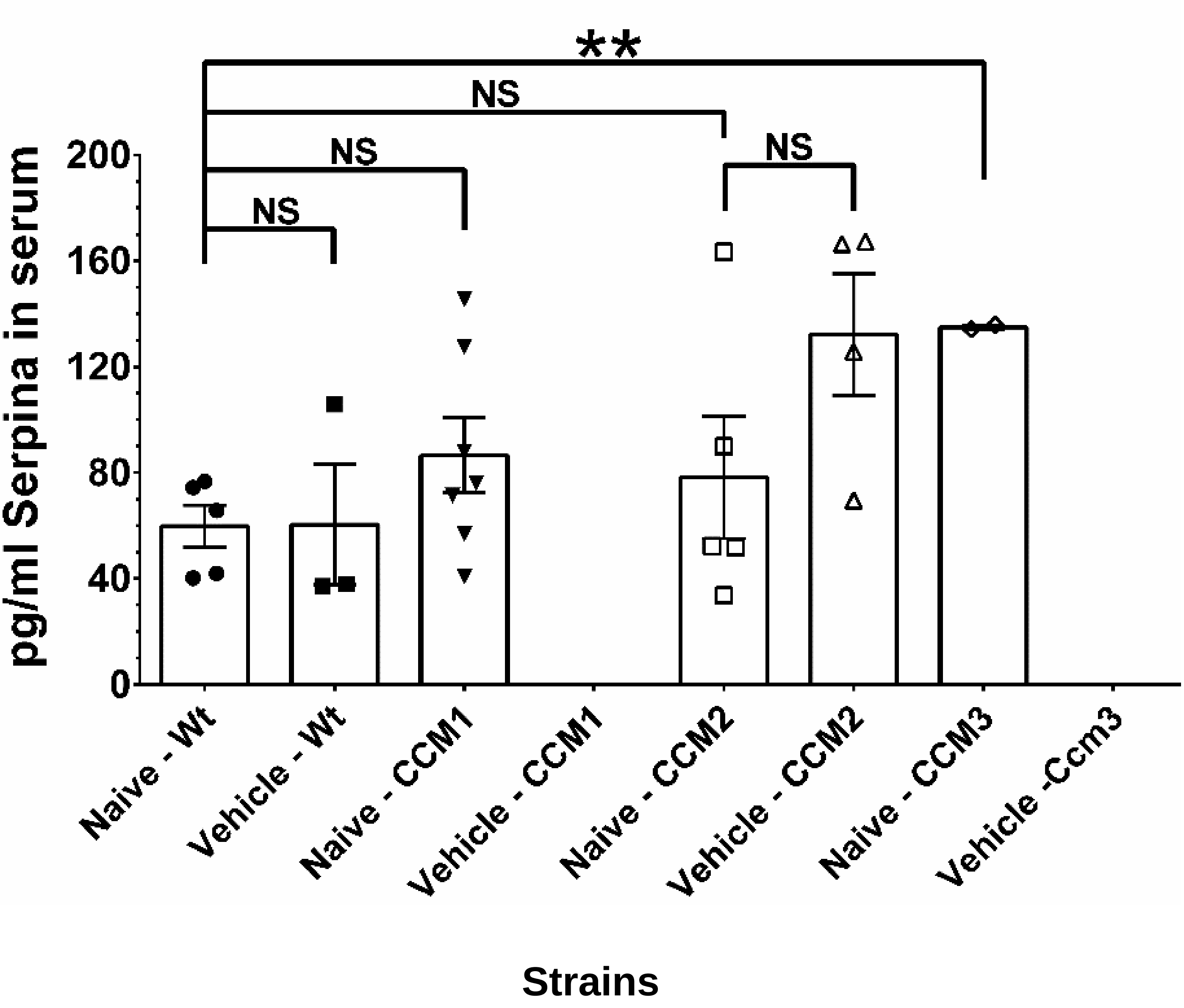

**
NS
NS
NS
NS
200
160
120
80
40
0
pg/ml Serpina in serum
Naive - Wt
Vehicle - Wt
Naive - CCM1
Vehicle - CCM1
Naive - CCM2
Vehicle - CCM2
Naive - CCM3
Vehicle -Ccm3
Strains

Fig. S7C

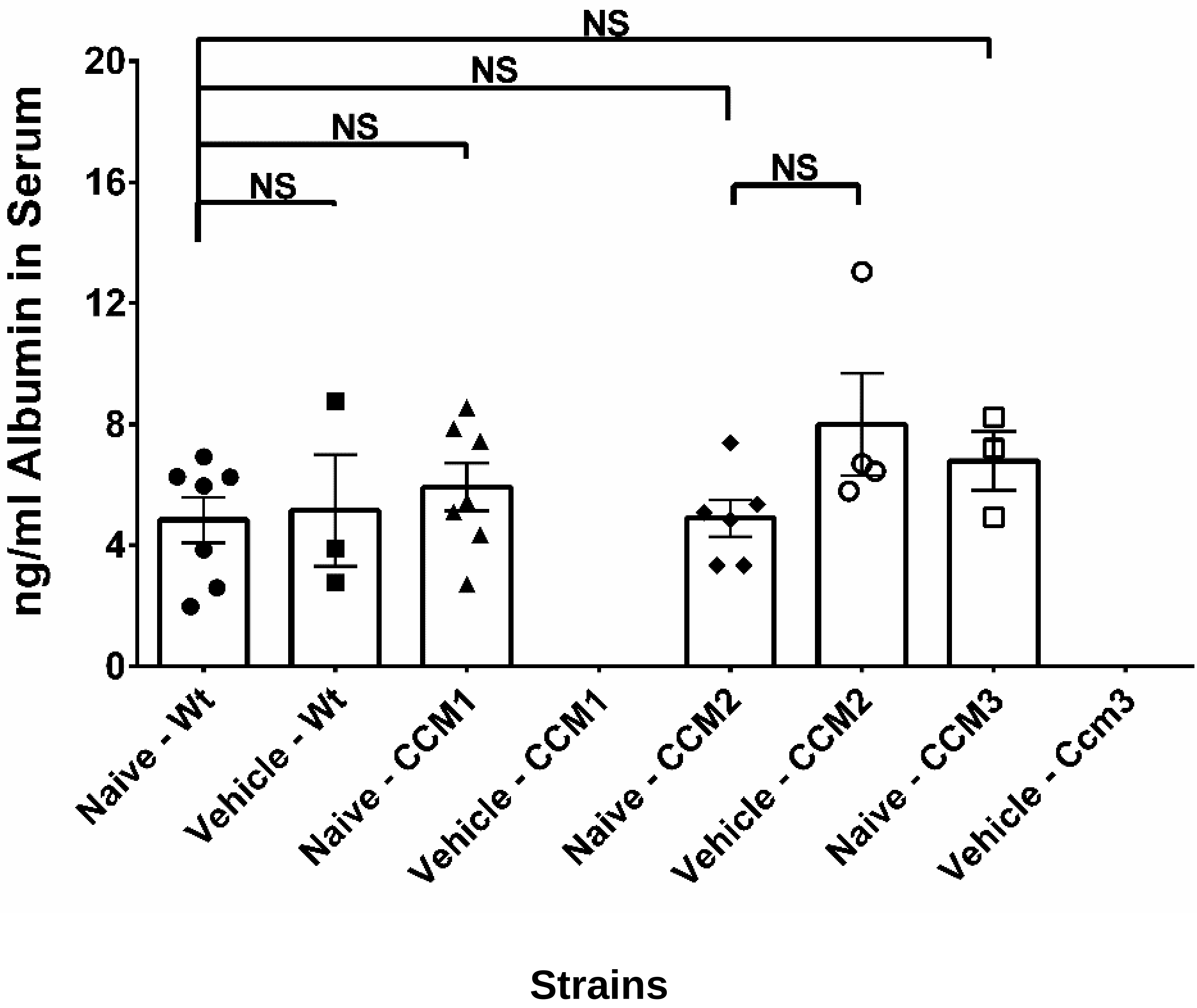

NS
NS
NS
NS
NS
ng/ml Albumin in Serum
20
16
12
8
4
0
Naive - Wt
Vehicle - Wt
Naive - CCM1
Vehicle - CCM1
Naive - CCM2
Vehicle - CCM2
Naive - CCM3
Vehicle - Ccm3
Strains
